# Optimizing Therapeutics for Intratumoral Cancer Treatments: Antiproliferative Vanadium Complexes in Glioblastoma

**DOI:** 10.3390/ijms26030994

**Published:** 2025-01-24

**Authors:** Andrew C. Bates, Kameron L. Klugh, Anna O. Galaeva, Raley A. Patch, John F. Manganaro, Skyler A. Markham, Emma Scurek, Aviva Levina, Peter A. Lay, Debbie C. Crans

**Affiliations:** 1 Department of Chemistry, Colorado State University, Fort Collins, CO 80523, USA; acbates@rams.colostate.edu (A.C.B.); kameron.klugh@colostate.edu (K.L.K.); anna.galaeva@colostate.edu (A.O.G.); raley.patch@colostate.edu (R.A.P.); john.manganaro@colostate.edu (J.F.M.); skyler.markham@colostate.edu (S.A.M.); emma.scurek@colostate.edu (E.S.); 2 School of Chemistry, The University of Sydney, Sydney, NSW 2006, Australia; aviva.levina@sydney.edu.au; 3 Cell and Molecular Biology Program, Colorado State University, Fort Collins, CO 80523, USA

**Keywords:** innovative chemotherapeutic agents, cancer, intratumoral injection, glioblastoma, optimizing drugs for intratumoral injection, vanadium, oxovanadium Schiff base catecholate complexes, sterically hindered catechol, hydrophobic Schiff base scaffold

## Abstract

Glioblastoma, an aggressive cancer, is difficult to treat due to its location, late detection, drug resistance, and poor absorption of chemotherapeutics. Intratumoral drug administration offers a promising potential treatment alternative with localized delivery and minimal systemic toxicity. Vanadium(V) coordination complexes, incorporating Schiff base and catecholate ligands, have shown effects as antiproliferative agents with tunable efficacy and reactivity, stability, steric bulk, hydrophobicity, uptake, and toxicity optimized for the intratumoral administration vehicle. A new series of oxovanadium(V) Schiff base–catecholate complexes were synthesized and characterized using nuclear magnetic resonance (NMR), UV-Vis, and infrared spectroscopy and mass spectrometry. Stability under physiological conditions was assessed via UV-Vis spectroscopy, and the antiproliferative activity was evaluated in T98G glioblastoma and SVG p12 normal glial cells using viability assays. The newly synthesized [VO(3-tBuHSHED)(TIPCAT)] complex was more stable (*t*_1/2_ ~4.5 h) and had strong antiproliferative activity (IC_50_ ~1.5 µM), comparing favorably with the current lead compound, [VO(HSHED)(DTB)]. The structural modifications enhanced stability, hydrophobicity, and steric bulk through substitution with *iso*-propyl and *tert*-butyl groups. The improved properties were attributed to steric hindrance associated with the new Schiff base and catecholato ligands, as well as the formation of non-toxic byproducts upon degradation. The [VO(3-tBuHSHED)(TIPCAT)] complex emerges as a promising candidate for glioblastoma therapy by demonstrating enhanced stability and a greater selectivity, which highlights the role of strategic ligand design in developing localized therapies for the treatment of resistant cancers. In reporting the new class of compounds effective against T98G glioblastoma cells, we describe the generally desirable properties that potential drugs being developed for intratumoral administration should have.

## 1. Introduction

The treatment of various forms of cancer remains a significant challenge in modern medicine. Specifically, glioblastoma is an aggressive form of brain cancer that currently is difficult to treat and has no cure. The average survival for glioblastoma patients after a diagnosis is typically 12–18 months, with an overall five-year survival rate of 5–10% [1,2,3,4,5,6,7,8,9]. The standard of care for glioblastoma includes maximum surgical resection of the tumor tissue followed by radiation and chemotherapy with temozolomide (TMZ), which has achieved only modest improvements in patient survival and ultimately is met with tumor recurrence and developed drug resistance [8].

The primary issues with the treatment of glioblastoma are an inability of current treatment methods to effectively reach the tumor because of its location, its late detection, and poor absorption manifested by an inability of the drug to cross the blood–brain barrier (BBB). In addition, chemotherapeutic treatments of glioblastoma with cisplatin have shown limited effects in part because the biological system develops resistance, and toxicity limits the dose being administered [10,11]. Even when placed directly into the brain within standard delivery systems, many chemotherapeutic drugs are limited by severe systemic toxicity and inefficient penetration of brain tumor tissue [12]. Thus, there is an urgent need for the development of drugs that can successfully treat aggressive cancers such as glioblastoma. We are developing new metal-based complexes that have potential properties that can be leveraged to combat aggressive and hard-to-reach tumors in the brain [13].

Currently, several transition metal-based drugs are available on the market and in clinical trials. For instance, platinum-based compounds like cisplatin, oxaliplatin, and carboplatin are widely used in clinical settings and are effective against various types of cancers [14,15,16]. However, drug development with other transition metals, including ruthenium, titanium, copper, osmium, iron, cobalt, and vanadium complexes, was reported [17,18,19,20,21,22,23,24,25,26]. The use of platinum-based drugs alone or in combination with other drugs is being developed for intratumoral injections (ITI). ITI is a drug delivery method that is used with increasing frequency in clinical trials because it delivers drugs to a specific location and can access inaccessible tumors (Table 1 and Table S1 in Supplementary Materials) [27,28,29,30,31,32,33]. At least 33 current and recent clinical trials using ITI and related techniques with platinum-based anticancer drugs are listed in the National Institute of Health’s (NIH) public database, https://clinicaltrials.gov (Accessed 19 July 2024). Although some of these clinical trials include novel formulations, they still use established chemotherapeutics with many side effects. In this work, we describe the development of a new class of potential drugs in the application for treatment of glioblastoma and discuss the ideal properties of drugs used for intratumoral administration.

**Figure 1:**
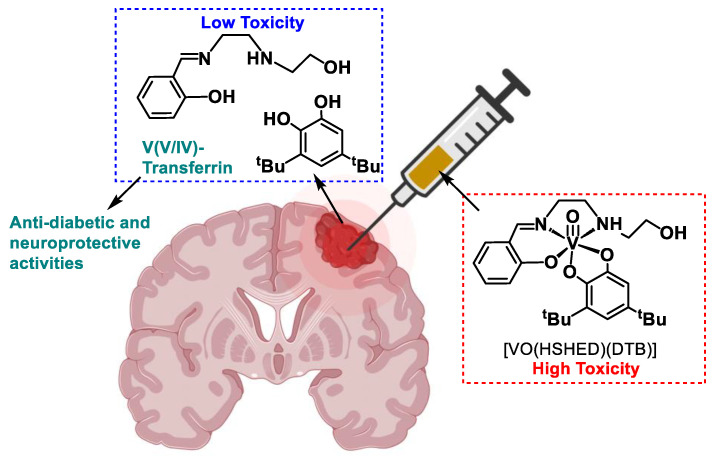
Proposed mode of action of typical V(V) Schiff base catecholate complex with [VO(HSHED)(DTB)] in intracellular injections into inoperable brain cancers, such as glioblastoma [13,28,37,75]. Figure was created using BioRender with permission (https://biorender.com/, accessed 14 January 2025).

In recent decades, vanadium compounds were studied as therapeutic agents for various human conditions, including diabetes, cardiovascular disease, and cancer [13,34,35,36,37,38,39,40,41,42,43,44,45,46,47,48,49,50,51,52,53,54,55,56]. Importantly, recent reports highlight the anticancer potential of vanadium (IV/V) coordination complexes [13,25,26,28,35,38,39,40,41,45,51,57,58,59,60,61,62,63,64,65,66,67,68]. The complexes work through a plethora of biological mechanisms, which include signal transduction, phosphatase inhibition, protein interaction, reactive oxygen species, and other mechanisms. Several classes of vanadium compounds were reported as anticancer agents, of which one class is non-innocent vanadium(V) Schiff base complexes [13,26,35,45,46,47,48,49,62,66,67,68,69,70,71,72,73,74,75]. These non-innocent vanadium complexes were designed specifically for intratumoral injections due to their ability to break down before diffusing or being transported out of the tumor into healthy tissue (see Figure 1) [13,28,66,75]. These non-innocent vanadium(V) coordination complexes feature a Schiff base backbone and a redox-active ligand. The decomposition products of these V(V) complexes, such as V(V/IV) species, can bind to proteins in the blood, such as transferrin, and can alleviate some of the side effects of chemotherapy on healthy brain tissue [13,28,37,68,75]. The stability of these complexes is strongly influenced by the ligand structure and environment [66,76]. The redox-active nature of both the metal center and the bidentate catecholate ligand contributes to the non-innocent behavior of these complexes, allowing them to participate in redox reactions. Catechols, which are known for their redox properties and ability to generate ROS, and are categorized as Pan Assay Interference Compounds (PAINS) due to frequent false positives in drug screening assays, which are often attributed to nonspecific interactions [77]. Despite this, catechols and related moieties (hydroquinones or quinones) are present in effective drugs like doxorubicin for cancer and carbidopa for Parkinsons [78,79]. Additionally, polyphenols and flavonoids containing catechols are linked to health benefits, including antioxidant, anti-inflammatory, and anticancer properties, with mechanisms such as FAS inhibition and tumor suppression [80,81]. This highlights their therapeutic potential for advanced treatments of diseases like cancer. The Schiff base moiety plays a crucial role that enhances the activity of the metal, regulates its reactivity, and does not contribute to toxicity upon dissociation of the coordination complex. Moreover, Schiff bases are increasingly recognized for their anticancer properties, making them an ideal backbone for designing metal complexes aimed at therapeutic applications, particularly in cancer treatment [82,83]. In this work, we explore if increasing steric bulk and hydrophobicity in the ligand shell improves the antiproliferative effects of the resulting V(V) Schiff base complexes.

The growing interest in the application of intratumoral therapeutics, such as ITI and related techniques listed in Table 1, suggests these approaches will soon become mainstream. However, as shown in Table 1, drugs that were demonstrated to be toxic systemically are still used, as evidenced by the many clinical trials for standard platinum-based drugs that are underway. It is important to recognize that these platinum drugs still invoke toxicity, even when locally delivered at an overall lower concentration to a tumor site since some of the intact drugs will diffuse out of the tumor. We are working on a class of compounds intended to react immediately upon administration and form less toxic side products, as illustrated in Figure 1 with our lead compound [VO(HSHED)(DTB)] [13].

**Figure 2:**
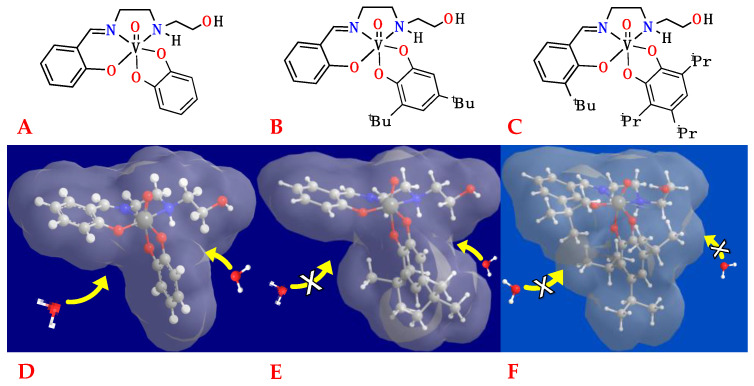
Structures of (**A**) [VO(HSHED)(CAT)], (**B**) [VO(HSHED)(DTB)], (**C**) [VO(3-tBu-HSHED)(TIPCAT)]. Compounds (**A**) and (**B**) represent [VO(HSHED)] framework [66], whereas compound C features novel [VO(3-tBuHSHED)] framework substituted with 3,4,6-tri-*iso*-propyl catechol (TIPCAT). Corresponding 3D structures (**D**–**F**) were calculated in Gaussian using B3LYP functionals and 3-21g* basis set. Calculated structures are displayed with solvent accessibility surface generated with ChemBio3D Ultra.

The current lead compound, [VO(HSHED)(DTB)] (where HSHED is *N*-(salicylideneaminato)-*N*′-(2-hydroxyethyl)-1,2-ethanediamine and DTB is 3,5-di-*tert*-butylcatechol), Figure 2B, exhibited the greatest resistance to hydrolysis in cell culture medium (*t*_1/2_ ~5 min at 25 °C) so far [66]. Furthermore, this complex exhibited an IC_50_ value of 1–2 µm, a relatively low IC_50_ value, especially when compared to treatments with cisplatin. From these studies, we gained structural insight into what is most effective in these types of complexes [13,66,67,75]. Three compounds investigated in this study are displayed in Figure 2, showing the current lead compound, a complex with an unsubstituted catechol (A), and the best complex of the new compounds designed in this study (C). The structures are shown in 2D, and a 3D representation illustrates how the structural modifications impact the solvent-accessible surface of the complex and potentially improve hydrolytic stability. We hypothesize that optimizing this class of compounds by modifying the ligand environment and structure will enhance their antiproliferative properties. By selectively introducing steric hindrance in the ligands, improved stability is observed, as well as greater antiproliferative efficacy against glioblastoma T98G cells.

## 2. Results

### 2.1. Development of V(V) HSHED Complexes

Herein, we report the comparison of the solution and anticancer properties of the known vanadium(V) HSHED Schiff base complexes [66] with a series of new oxovanadium(V) complexes featuring a 3-tBuHSHED Schiff base backbone and the new catechols of interest, DIPCAT and TIPCAT. The addition of the 3-*tert*-butyl group on the Schiff base backbone was undertaken to increase both hydrophobicity and steric bulk. The catechol substitution pattern was explored to determine the role of placements of substituents. The lipophilicity and partition coefficient (log*P*) of compounds were calculated to compare their hydrophobicity and can be found in the Supplementary Material (Tables S2 and S3). The program Chemicalize by ChemAxon was used to estimate the hydrophobicity of these complexes and confirmed the expectation of a hydrophobicity scale of [VO(HSHED)(CAT)] < [VO(HSHED)(DIPCAT)] < [VO(HSHED)(DTB)] < [VO(HSHED)(TIPCAT)]. A similar pattern was observed for the [VO(3-tBuHSHED)(CAT)] < [VO(3-tBuHSHED)(DIPCAT)] < [VO(3-tBuHSHED)(DTB)] < [VO(3-tBuHSHED)(TIPCAT)] and, as expected, the hydrophobicity for the latter systems is significantly higher than the parent series. We describe the preparation, characterization, stability, and biological properties of the new series of compounds.

#### 2.1.1. Di and Tri-Substituted Catechols

**Figure 3:**
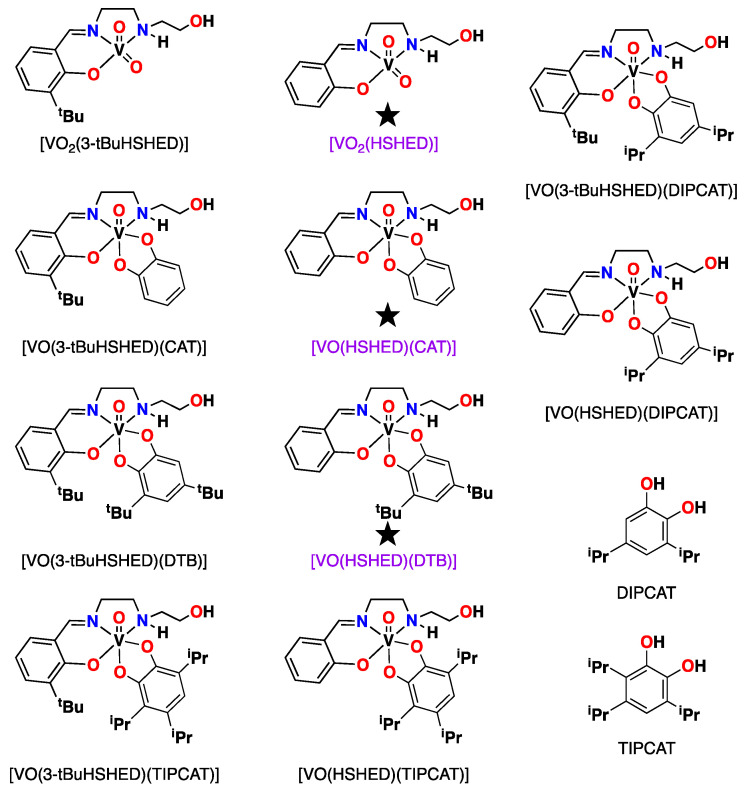
Structures of seven novel V(V) Schiff base complexes used in this work included with catechol ligands TIPCAT and DIPCAT. Complexes that are reported in previous studies are indicated with star and have purple abbreviated name.

The most stable and active oxovanadium Schiff base complexes were previously reported with 3,5-di-*tert*-butylcatechol (DTB) [66,67]. To investigate a catechol ligand with a substitution pattern similar to DTB, albeit with a smaller alkyl group, to test the possibility that such a group would cause similar effects on the stability of the complex, we synthesized 3,5-di-*iso*-propyl-catechol (DIPCAT). This synthesis also resulted in a tri-substituted ligand, 3,4,6-tri-*iso*-propyl catechol (TIPCAT), which was therefore also used in syntheses of complexes for comparison (Figure 3). The synthesis of these *iso*-propyl catechols was reported in the literature [84,85], but here, we used milder and more environmentally friendly conditions by replacing the perchloric acid with sulfuric acid, which also resulted in slightly higher yields. The ligands were characterized using routine methods and compared to the literature reports [84,85].

#### 2.1.2. New Oxovanadium Schiff Base Complexes

The oxovanadium Schiff base complexes of both parent and modified Schiff base complexes with the DIPCAT and TIPCAT ligands were prepared with the intent of modifying mainly complex stability. Most notably, the use of TIPCAT had the ability to further protect the vanadium atom in the complexes from hydrolysis through the addition of substituents on the catechol in both the 3 and 6 positions, which had the potential to further “shield” the vanadium core and increase hydrolytic stability. Thus, these complexes will allow us to compare the complexes with the 3,4,6-tri-*iso*-propyl catechol ligand (TIPCAT) to the previously reported lead compounds that contain ligands such as 3,5-di-*tert*-butyl catechol (DTB) [13,66].

### 2.2. Compound Characterization

#### Characterization of Solution Structures Using Multinuclear NMR Spectroscopy

**Figure 4:**
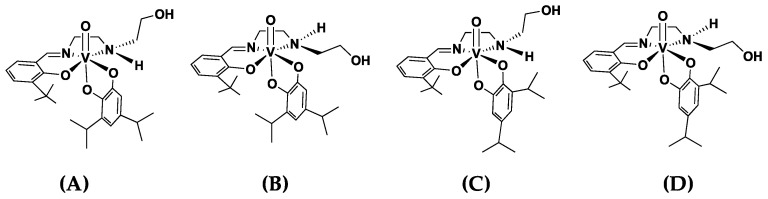
Structures of four isomeric forms of [VO(3-tBuHSHED)(DIPCAT)] are shown (only one set of enantiomeric complexes are shown of racemic mixture that forms in solution). Structure (**A**) was determined to be the major isomer structure indicated by NOESY interactions. Specific interactions used to determine isomer structure are described in text. Structures (**B**–**D**) are the minor isomers.

**Figure 5:**
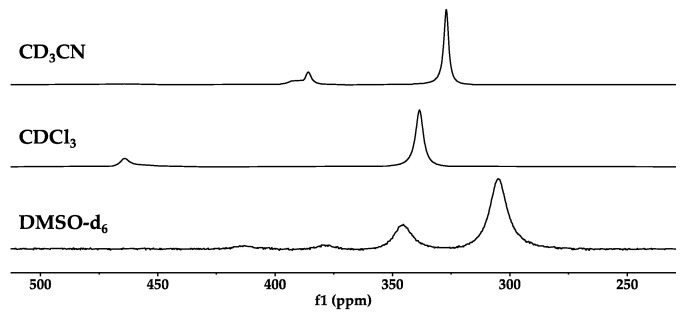
Stacked ^51^V NMR spectra of [VO(3-tBuHSHED)(DIPCAT)] in CD_3_CN, CDCl_3_, and DMSO-*d_6_*_._ Major isomer peak is associated with structures A/B in Figure 4, which indicates orientation of catechol ligand. Solvent effects strongly influence chemical shift in signals observed in ^51^V NMR. Minor signals attributed to structures in Figure 4B–D are not identified as specific isomer structures.

Both ^1^H and ^51^V NMR were collected for the compounds and can be found in Supplementary Material (Figures S1–S17). If the compounds were pure, ^51^V NMR spectra in several solvents would show different isomer signals and ratios. One enantiomeric form of the racemic isomers that potentially can form in a solution are shown in Figure 4. The ^51^V NMR spectra were measured using 10 mM solutions of [VO(3-tBuHSHED)(DIPCAT)] in CD_3_CN, CDCl_3_, and DMSO-*d*_6_ (Figure 5). In CD_3_CN and CDCl_3_, two sharp signals and one broad, overlapping signal were observed at different chemical shifts, with the major isomer representing 78.7% and 84%, respectively. In DMSO-*d*_6_, four broad signals were observed in a ratio of 1.00:0.31:0.04:0.03, making the major isomer 72.5%. None of the observed signals were due to the Schiff base precursor that has a signal near −540 ppm; therefore, the observed spectra did not indicate decomposition or impurities. Furthermore, this same isomeric distribution was observed in the ^1^H NMR spectra for the imine proton in the 8.6 to 8.9 ppm region, as described in Figure 5.

**Figure 6:**
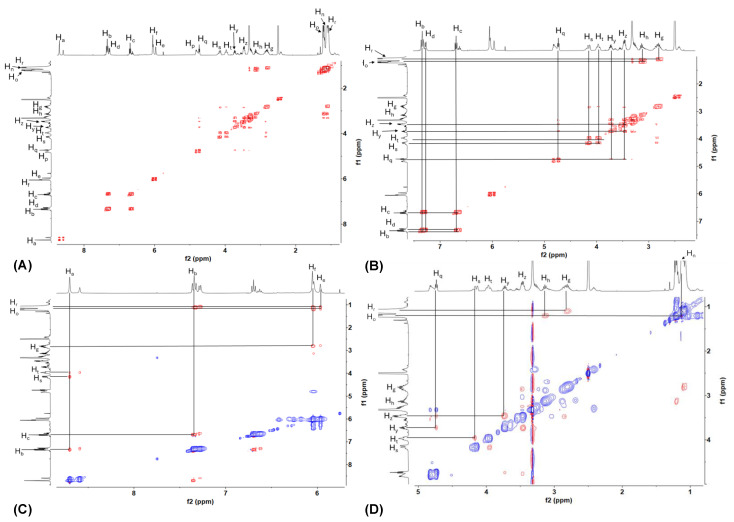
The structure of [VO(3-tBuHSHED)(DIPCAT)] is shown with proton-labeling scheme, see Figure 7. ^1^H-^1^H 2D COSY and ^1^H-^1^H 2D NOESY NMR (400 MHz) spectra were run at 10 mM in DMSO-*d_6_* at ambient temperature. (**A**) Full ^1^H-^1^H COSY spectrum of [VO(3-tBuHSHED)(DIPCAT)]. (**B**) Zoom in of ^1^H-^1^H COSY spectrum showing correlations for ethylene and aromatic spin systems. (**C**) Zoom in of aromatic region of ^1^H-^1^H NOESY spectrum, which showed interactions between aromatic protons and *iso*-propyl groups on catechol ligand. (**D**) Zoom in of ethylene region of ^1^H-^1^H NOESY spectrum of [VO(3-tBuHSHED)(DIPCAT)]. Red intensity contours represent positive NOEs and blue intensity contours represent negative NOEs.

**Figure 7:**
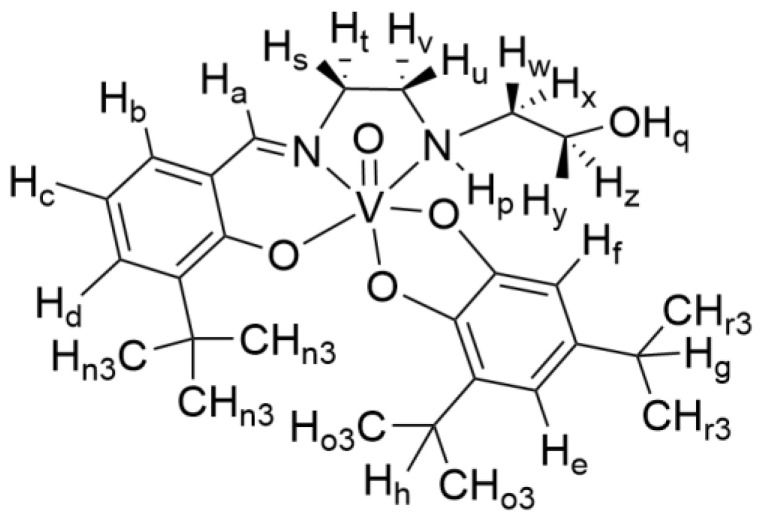
Structure of [VO(3-tBuHSHED)(DIPCAT)] with proton-labeling scheme and corresponding NMR spectra is displayed in Figure 6.

The ^1^H NMR spectra in DMSO-*d_6_* showed higher resolutions compared to the spectra in CD_3_CN and CDCl_3_. This led to the structural solution studies being carried out in DMSO-*d_6_*. To assign the protons for [VO(3-tBuHSHED)(DIPCAT)], the spectra were integrated and the protons in the aromatic region were identified first, beginning with Schiff base proton H_a_ (s, 8.70 ppm). The lack of correlation of H_a_ with other protons in the COSY spectrum (Figure 6 and Figure 7) supported the Schiff base proton assignment. Integrations in the ^1^H NMR spectra, cross peaks in the ^1^H-^1^H COSY spectrum, and coupling constants allowed for the assignments of H_b_ (d, 7.34 ppm), H_c_ (t, 6.70 ppm), and H_d_ (d, 7.28 ppm). The remaining aromatic protons H_e_ (s, 5.67 ppm) and H_f_ (s, 6.05 ppm) were assigned using the NOESY spectra, which showed crosstalk with either one (H_f_) or two (H_e_) *iso*-propyl groups.

This interaction also helped assign the methyl groups belonging to the *iso*-propyl substituents on the catechol ring, H_o_ (d, 1.21 ppm) and H_r_ (d, 1.09 ppm). Once the identity of the *iso*-propyl groups was known, the assignment of the single proton on each group was confirmed using the COSY spectrum to define protons H_h_ (sept, 3.13 ppm) and H_g_ (sept, 2.80 ppm). Additionally, COSY signals enabled the distinction of protons residing on the ethylenediamine backbone and ethanolamine arm; specifically, off-diagonal signals between H_s_ (4.16 ppm) and H_t_ (3.96 ppm) along with H_y_ (3.73 ppm) and H_z_ (3.46 ppm) corresponded to the first protons on the backbone and ethanol arm, respectively. The HOD peak, at roughly 3.32 ppm, made further proton assignments in this region of the molecule nontrivial, as overlapping and isomeric signals complicated the assignments. For the Schiff base proton assignment, H_a_ (8.70 ppm) was confirmed using NOESY cross peaks between H_a_ and H_b_, and additional interactions between H_a_ and ethylene protons H_s/t_.

Assignment of the major isomer structure of [VO(3-tBuHSHED)(DIPCAT)] in solution required the ^1^H-^1^H 2D NOESY in DMSO-*d*_6_ to view through-space interactions between protons. NOE cross peaks occur typically at distances less than 5 Å. The NOE cross peak between H_n_ and H_o_ indicated that in the major isomer solution, one *iso*-propyl group (H_o_) is positioned closer to the Schiff base and within 5 Å of the protons on the *tert*-butyl group (H_n_). The analysis of the 3D structure in the solution for [VO(3-tBuHSHED)(TIPCAT)] was conducted similarly; however, because this catechol has an *iso*-propyl group in positions 3 and 6 in the catechol, the major isomer in the solution is very similar in energy compared to the isomer with the rotated catechol.

Therefore, the orientation of the catechol, as shown in Figure S20, with the third *iso-*propyl group *trans* to the axial catechol oxygen in the complex, is likely to be very similar in energy to the corresponding complex with the third *iso-*propyl group *meta* to the axial catechol oxygen atom. These considerations are consistent with the observation of two major peaks with similar intensities in the ^51^V NMR spectrum shown in the Supplementary Materials. Information is provided for the additional compounds prepared, and details are provided in the Supplementary Materials (Figures S18–S20).

### 2.3. New Vanadium Schiff Base Catecholate Complex Stability

#### 2.3.1. Stability of Complexes in Cell Culture Media

**Figure 8:**
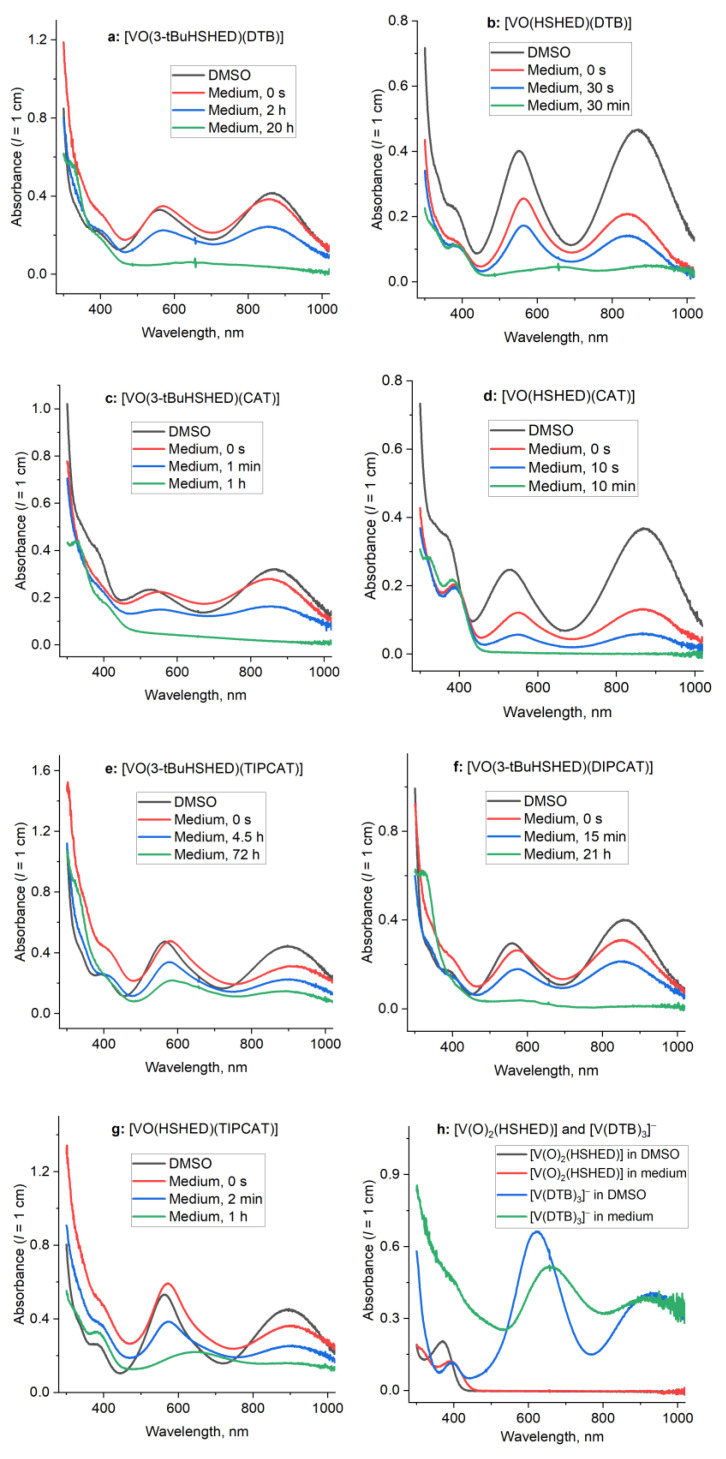
Typical time-dependent spectra of V(V) complexes used in this study (**a**–**g**) (0.10 mM; see Figure 3 for structures) in cell culture medium at 310 K in comparison with corresponding spectra in DMSO at 295 K. Time-dependent spectra showing the formation of V(V)-catecholato metabolites for DTB compounds in DMSO and medium (**h**). Kinetic analyses of time-dependent spectra are shown in Figures S36–S42 (Supplementary Materials), and calculated half-life times are listed in Table 2.

The time-dependent UV-Vis-NIR spectra of the V(V) complexes in cell culture medium at 310 K were measured at the highest concentration used in cell culture experiments (0.10 mM). Typical time-dependent spectra are shown in Figure 8 in comparison with those of freshly prepared solutions of the same complexes in DMSO (0.10 mM, 295 K). All the mixed-ligand V(V) complexes showed two intense absorbance bands at ~550 nm and ~850 nm, which demonstrated their decomposition under biologically relevant conditions [37]. As shown in Figure 8, various complexes decomposed in cell culture medium on the second (Figure 8b–d), minute (Figure 8f,g), or hour (Figure 8a,e) timescales. The spectra of the decomposition products (green lines in Figure 8a–g) were consistent with the release of catecholato ligands and the formation of [V(O)_2_(HSHED)] or [V(O)_2_(3-tBu(HSHED)] (390–400 nm band, black and red lines in Figure 8h) [37]. In some instances, the decomposition products showed prominent absorbance bands at 600–650 nm, as is consistent with the formation of V(V) tris-catecholato species (blue and green lines in Figure 8h) [37,86].

Half-life times of the mixed-ligand V(V) complexes (Figure 8) under cell culture conditions were calculated based on the results of global kinetic analyses of UV-vis-NIR spectra (see Figures S36–S42 in Supplementary Material) and the half-lives are summarized in Table 2. The presence of a bulky *tert*-butyl group on the Schiff base ligand also increased the lifetime of the complexes either ~6–7-fold ([VO(3-tBuHSHED)(CAT)] vs. [VO(HSHED)(CAT)]) or 170–190-fold ([VO(3-tBuHSHED)(DTB)] vs. [VO(HSHED)(DTB)] and [VO(3-tBuHSHED)(TIPCAT)] vs. [VO(HSHED)(TIPCAT)]). For a series of complexes with 3-tBuHSHED Schiff base ligands, the stability increased in the order of increasing steric bulk of catecholato ligands, CAT < DIPCAT < DTB < TIPCAT, which is in agreement with the earlier studies [37,66,67,75].

The increase in steric bulk on the Schiff base ligand by the introduction of a *tert*-butyl substituent on the Schiff base scaffold resulted in stabilization of the [VO(3-tBuHSHED)(DTB)] compared to [VO(HSHED)(DTB)] by 180-fold, as evidenced by the complex’s lifetime. Positioning this group in the 3-position, adjacent to the coordinated phenol, is believed to provide optimal shielding for the vanadium coordination sphere. The lifetimes of the complexes with the modified HSHED scaffold, 3-tBuHSHED, and with the novel catechol ligands, TIPCAT and DIPCAT, in cell culture medium at 310 K increased, with [VO(3-tBuHSHED)(TIPCAT)] being more stable than [VO(3-tBuHSHED)(DIPCAT)]. Both of these complexes have longer half-lives than the other complexes listed in Table 2, excluding [VO(3-tBuHSHED)(DTB)], but including the current lead compound [VO(HSHED)(DTB)]. This order indicated that the increase in steric bulk both on the Schiff base scaffold and on the catechol ligand in TIPCAT and DIPCAT complexes was essential for increased lifetimes and the stability of V(V)–Schiff base catecholate complexes under biologically relevant conditions.

#### 2.3.2. Antiproliferative Properties of Oxovanadium Schiff Base Catecholate Complexes

**Figure 9:**
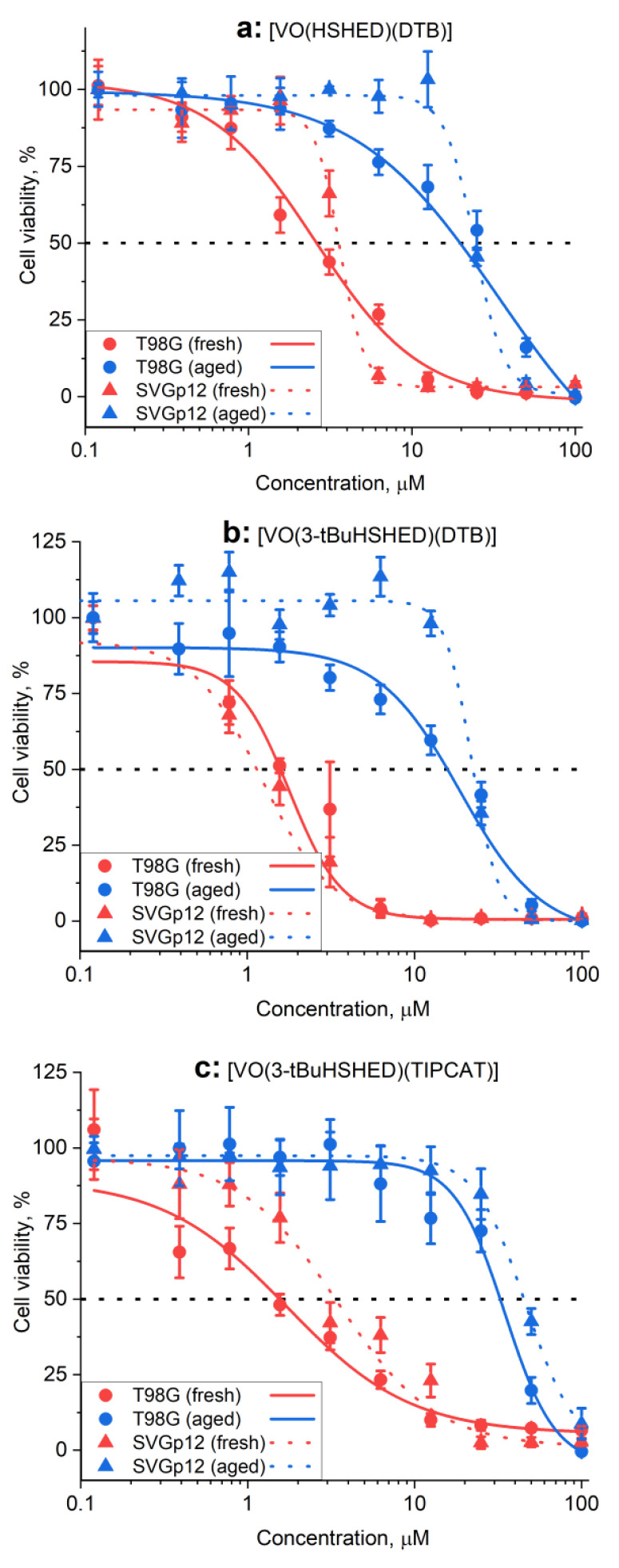
Typical concentration–viability plots for antiproliferative activities (72 h assays) of fresh (pre-incubated with medium for <1 min) and aged (pre-incubated with medium for 24 h at 310 K) V(V) complexes in T98G and SVG p12 cells: (**a**) [VO(HSHED)(DTB)]; (**b**) [VO(3-tBuHSHED)(DTB)]; (**c**) [VO(3-tBuHSHED)(TIPCAT)]. Dots and error bars represent mean values and standard deviations of six replicate wells, and lines are sigmoidal fits of experimental data used for calculations of IC_50_ values (Table 2). Corresponding data for all compounds included in Table 2 are shown in Figures S43–S45, Supplementary Materials.

The antiproliferative effects of the complexes on human glioblastoma (T98G) cells were determined for both compounds prepared immediately before administration, referred to as fresh, and after 24 h of incubation in cell assay media, referred to as aged solutions. The antiproliferative effects are summarized in Table 2, see Figure 9 and Figures S43–S45, Supplementary Materials. The antiproliferative effects of the complexes were also determined in normal human fetal glial cells from the brain, the SVG p12 cell line, of fresh compounds and aged compounds; the results are summarized in Table 2.

The effects of freshly prepared and intact complexes are compared to that of the aged complexes in cell culture medium for 24 h at 310 K, which led to the practically complete decomposition of the complexes (Figures S36–S42). While the activities of the complexes with DTB, DIPCAT, and TIPCAT ligands decreased 5–20-fold after aging, the aged solutions of the complexes with CAT ligands were more toxic than the freshly prepared solutions (Table 2). This demonstrates that for all the complexes except for the CAT complexes, the intact complexes are more potent than the decomposed complexes. This observation was made both in the cancer (T98G) cells as well as the normal glial brain (SVG p12) cells. [VO(3-tBuHSHED)(TIPCAT)], as shown in Table 2 and in Figure 9c, has activity in SVG p12 cells that is a factor of two less in T98G cells, which is the best result among all the studied complexes. This shows that changes in the ligand structure can lead to increased selectivity of the complexes for cancer cells. The results show that these complexes may not target specific pathways in cancer cells compared to SVG p12 cells as we previously found for studies with [VO(HSHED)(DTB)] when comparing the effects on T98G with the HFF-1 (normal human foreskin fibroblasts) [13].

We did not find a convincing correlation between the stability of the complexes in cell culture media and their antiproliferative activity in human glioblastoma (T98G) cells (Table 2). Although the two complexes with the longest lifetimes in media (the most stable complexes), [VO(3-tBuHSHED)(TIPCAT)] and [VO(3-tBuHSHED)(DTB)], showed the highest activity treated with fresh compound (IC_50_ ~1.5 μM in a 72 h assay), the differences are very small and only one of the new complexes showed a modest selectivity for cancer (T98G) vs. non-cancer (SVG p12) cells.

The toxicity of decomposition products to cancer and non-cancer cells was highest for the CAT complexes. Such information is important for the potential use of these complexes in intratumoral injections [13]. A comparison with the effects of aged free ligands and vanadate in T98G cells (Table 2) has shown that the toxicity of the aged solutions was in all cases due to the released catechols and V(V) species. Furthermore, in the case of unsubstituted CAT, these reactions are more likely to produce highly toxic species, such as semiquinone radicals and V(V) peroxide complexes [37].

Overall, considering the high antiproliferative activity in T98G cells as intact complexes, the slightly lower activity in non-cancer SVG p12 cells, and the significant decrease in activity on aging (~20-fold), the complex [VO(3-tBuHSHED)(TIPCAT)] can be regarded as the slightly better complex among the studied V(V) complexes for intratumoral injection applications. It may also offer a slight improvement over the previously proposed lead compound, [VO(HSHED)(DTB)]. In addition, the significantly extended lifetime of the former complex under cell culture conditions (Table 2) offers an additional advantage for the development of appropriate pharmaceutical formulations [13].

## 3. Discussion

The objective of this work was to examine how sterically hindered substituents on the Schiff base and catechol affect the stability of V(V) Schiff base catecholates, aiming to optimize the properties of this class of compounds. The addition of the *tert*-butyl group on the Schiff base scaffold provided additional steric bulk and hydrophobicity, which improved the stability of the Schiff base oxovanadium catecholate complexes, as shown in Table 2. Specifically, the half-lives of the two pairs of complexes increased by a factor of 7 for [VO(HSHED)(CAT)]/[VO(3-tBuHSHED)(CAT)]; and a factor of 180 for [VO(HSHED)(DTB)]/[VO(3-tBuHSHED)(DTB)] by the addition of the 3-*tert*-butyl group on the scaffold. This study also sought to compare the effects of *iso*-propyl-substituted catechols (TIPCAT or DIPCAT) with the *tert*-butyl substitution on the catechol (DTB)—notably, the comparison of the steric effects of *iso*-propyl groups versus *tert*-butyl groups on vanadium complex stability. Since the *iso*-propyl group is smaller than the *tert*-butyl group, the catechol with the two *iso*-propyl groups was anticipated to support a lower stability of resulting vanadium complexes. However, since *iso*-propyl groups are somewhat smaller, we were able to prepare the tri-*iso*-propyl catecholate (TIPCAT). Vanadium complexes with both the di- and tri-*iso*-propyl catecholates were thus prepared. The 3,5-di-*iso*-propyl catechol (DIPCAT) did not form complexes with improved stability compared to the complexes from the DTB catechol coordinated to either [VO_2_(HSHED)] or [VO_2_(3-tBuHSHED)]. However, the complexes formed from TIPCAT were found to be more stable than the complexes formed from the catechol with two *tert*-butyl substituents (DTB); that is, [VO(HSHED)(TIPCAT)] is more stable than [VO(HSHED)(DTB)] and [VO(3-tBuHSHED)(TIPCAT)] is more stable than [VO(3-tBuHSHED)(DTB)] (Figure 8a,e and Table 2). The observed increase in stability is attributed to the placement of two *iso*-propyl substituents at positions 3 and 6 on the catechol, which are adjacent to the coordinating catecholate oxygen atoms, and the half-lives of the pairs of complexes increased a factor of 170 for [VO(HSHED)(TIPCAT)]/[VO(3-tBuHSHED)(TIPCAT)], which demonstrated that the 3-*tert*-butyl group on the scaffold improved the stability of the complexes with DTB and TIPCAT catechols similarly. This arrangement, combined with an increased steric bulk on the scaffold, effectively shields the vanadium core from associatively activated hydrolysis and substitution reactions. These findings suggest that the position of the substituents on the catechol may have a more significant influence than their size in this context, representing a rare instance where an *iso-*propyl group provides greater stability than a *tert*-butyl group.

Steric hindrance plays a key role in controlling the lifetime of these compounds, which was also shown to be crucial for their antiproliferative activity. The two novel most active compounds in this study, [VO(3-tBuHSHED)(DTB)] and [VO(3-tBuHSHED)(TIPCAT)], had low IC_50_ values of ~2 mM in T98G glioblastoma cells and compared with our current known lead compound [VO(HSHED)(DTB)] (Table 2). The two most effective novel compounds had the longest lifetimes under cell culture conditions (1–5 h). The known [VO(HSHED)(DTB)] was only stable for ~30 s but still showed similar toxicity to the compounds with extended lifetimes. However, [VO(HSHED)(TIPCAT)] demonstrated slightly enhanced stability to [VO(HSHED)(DTB)] (~90 s) but was less effective in preventing cell proliferation. This may suggest that effective compounds require a combination of ligand scaffolds and catechols and indicate the tunability of this class of compounds. The best new compound [VO(3-tBuHSHED)(TIPCAT)] was not more potent than [VO(HSHED)(DTB)], but it was slightly more selective for cancerous cells over healthy glial cells. These stability observations highlight how structural modifications, such as scaffold modification and catechol substituent placement, can significantly influence the behavior and efficacy of vanadium complexes. This is particularly important given that these compounds are designed for intratumoral applications rather than oral administration and are not limited by the constraints of Lipinski’s “rule of five.” We have found that although these vanadium complexes are more hydrophobic than recommended by Lipinski’s constraints for drug-like molecules, they show potential for treatment of cisplatin-resistant glioblastoma. Indeed, not all successful drugs abide by Lipinski’s rules, one example being Venetoclax, which is used to treat chronic lymphocytic leukemia (CLL) or small lymphocytic lymphoma in the clinic.

For most effective intratumoral treatment, the ideal compounds must exhibit selectivity for tumor cells, demonstrate rapid cellular uptake, and initiate potent cytotoxic reactions immediately upon administration, ensuring swift and localized destruction of cancerous cells or tissue. However, once they have acted on the cancer cells, their cytotoxic effects should be reduced or completely lost so that when the drug diffuses into healthy tissue, no adverse effects are observed [13,28,66,75]. Thus, ideal compounds with sufficient reactivity and a short, effective lifetime are desirable, although the exact duration of the lifetime for optimal efficacy remains uncertain. Furthermore, the optimum lifetime would likely vary with cell and cancer type, which require tailored approaches for each drug. Additionally, we have learned that interaction with proteins such as serum albumin (HSA) can enable the compounds to slowly release and extend their lifetime to assist in the delivery of the compounds into cancer tissue [37]. This work optimized new vanadium compounds using various strategies to enable slow release, shorten lifetimes, and incorporate steric hindrance and hydrophobicity for effective administration. We have found that complexes with aqueous stability induced by hydrophobicity may be a favorable factor for intratumoral drug candidates.

To summarize, the Schiff base ligand frameworks we developed in this study significantly improved the hydrolytic stability of the V complexes through the strategic incorporation of *tert*-butyl and *iso*-propyl substituents. These aliphatic substituents enhanced stability and potentially promoted cellular uptake and membrane compatibility. We optimized the hydrophobic properties, enhanced cytotoxicity against glioblastoma cancer cells, and tailored their aqueous stability within the tumor microenvironment to mitigate adverse effects from the metabolites. [VO(3-tBuHSHED)(TIPCAT)] had an extended lifetime under conditions mimicking those within the tumor, which is consistent with its enhanced antiproliferative effects. Thus, our new compound [VO(3-tBuHSHED)(TIPCAT)] demonstrated greater stability compared to the lead compound. However, its similar efficacy with that of [VO(HSHED)(DTB)] in T98G glioblastoma cells suggests that stability is not the only factor influencing the antiproliferative effects. Nonetheless, a subtle increase in selectivity for cancer cells is observed. No great selectivity for T98G over SVG p12 cells was observed because both are rapidly dividing cell lines. Standard chemotherapies are well known to target rapidly dividing non-cancer cells, such as hair follicles. Future studies will test the toxicity of the complexes in non-dividing normal human cells, such as mature leukocytes (PMBC). One can speculate that other factors important to the antiproliferative effects include cellular uptake and bioprocessing, which will also be investigated in future work. The novel compounds reported in this work were designed for use in localized delivery to target malignant tumors, and our work provides proof-of-concept evidence on the properties of drugs for intratumoral delivery exerting such effects in T98G glioblastoma cells.

## 4. Materials and Methods

### 4.1. Experimental

#### 4.1.1. General Materials and Methods

Catechol (99%) and *N*-(2-hydroxyethyl)ethylenediamine (95%) were purchased from Oakwood Chemical (West Columbia, SC, USA, catalogue 005090, 245937) and used as received. 3,5-di-*tert*-butylcatechol (95%) and 3-*tert*-butyl salicylaldehyde (97%) were purchased from AmBeed (Arlington Heights, IL, USA, catalogue A296117, A230999) and used as received. Vanadyl sulfate hydrate (99.9%) and all organic solvents used were purchased from Thermo Fisher Scientific (Waltham, MA, USA, HPLC or ACS grade) and used without further purification. The solvents used in the synthesis were degassed prior to use with argon. Ultrapure argon (AR UHP300) from Airgas (Fort Collins, CO, USA) was used to keep inert atmosphere conditions and for degassing solutions. Pre-sterilized media and sterile plasticware used in the cell culture studies were purchased from Thermo Fisher Scientific Australia. The hydrolytic stability of the complexes was studied by UV-vis-NIR spectroscopy (AvaLight UV-vis/NIR Light Source and AvaSpex-UL S2048 Fiber-Optic Spectrophotometer). FTIR spectra were collected on a Thermo Nicolet iS-50 FTIR spectrometer with ATR crystals. NMR spectra were collected on a Bruker model AVANCE Neo400 spectrometer operating at either 105.2 or 400 MHz at ambient temperature. NMR solvents were purchased from Cambridge isotope laboratories and used as received. Electronic absorption (UV-vis-NIR) spectroscopy was performed on a Specord S600 diode-array spectrometer (Analytic Jena, Germany).

#### 4.1.2. General Synthesis

[VO_2_(HSHED)] derivatives were prepared as reported previously [66,87]. This study introduces a new Schiff base, [VO_2_(3-tBuHSHED)], and its complexes with select catecholate ligands. The new catecholate ligands tested in this study, TIPCAT and DIPCAT, were generated in the same preparation according to a modified and safer procedure than previously reported [84,85]. The synthesis of the main compound of interest, [VO(3-tBuHSHED)(TIPCAT)], which incorporates the new Schiff base and the catechol ligand (TIPCAT), is described in the main text. The related compound, [VO(HSHED)(DIPCAT)], was also synthesized but since it was significantly less stable in aqueous media, it was not evaluated further. Catecholate complexes were crystallized from a minimal amount of acetone or dichloromethane and hexanes, which varied depending on the scale and purity of the sample. Experimental details for the synthesis and characterization for the other compounds investigated in this study can be found in the Supplementary Materials.

*3,4,6-tri-iso-propyl catechol* (1) catechol (2.0 g, 20.0 mmol) and 2-propanol (4.6 mL, 60 mmol) were stirred together and heated to 60 °C. Sulfuric acid (4.3 mL, 80 mmol) was added dropwise, and the reaction mixture was refluxed for 4 h. The mixture was neutralized with ice water (100 mL) and sodium bicarbonate (solid) and then extracted with ethyl acetate (3 × 50 mL). The solvent was removed under reduced pressure and the crude product was purified via column chromatography in 10% ethyl acetate: hexanes to yield 2.36 g (50%) of 3,4,6-tri-*iso*-propyl catechol as a red solid. The product was further purified by recrystallization from hexanes to yield tan crystals. ^1^H NMR (δ, 400 MHz, CDCl_3_): 6.64 (s, 1H), 5.32 (s, 1H), 4.72 (s, 1H), 3.42–3.32 (m, 1H), 3.17 (hept, *J* = 6.5 Hz, 1H), 3.05 (hept, *J* = 6.9 Hz, 1H), 1.39 (dd, *J* = 7.1, 0.8 Hz, 6H), 1.27 (dd, *J* = 6.9, 0.8 Hz, 6H), 1.21 (dd, *J* = 6.9, 0.8 Hz, 6H. IR (cm^−1^, diamond ATR): 3369 (OH), 2959 (alkane CH), 2928 (alkane CH), 2868 (alkane CH).

*[VO_2_(3-tBuHSHED)]* 3-*tert*-butyl salicylaldehyde (3.12 g, 18.8 mmol) was dissolved in methanol (50 mL) and added to a solution of *N*-(2-hydroxyethyl)ethylenediamine (1.9 mL, 18.8 mmol) in methanol (50 mL). The yellow reaction mixture was refluxed for 2 h under an argon atmosphere. The mixture was cooled to room temperature and vanadyl sulfate hydrate (4.08 g, 22.6 mmol) dissolved in DI water (20 mL) was added to the reaction slowly over 10 min, turning the reaction mixture black. This mixture was allowed to stir for 3 h under argon. After 3 h, NaOH (1.51 g, 37.6 mmol) in DI water (30 mL) was added. At this time, any solid stuck to the sides of the reaction flask was scraped back into the solution. The resulting mixture was allowed to stir open to air for 12 h. The reaction was filtered and the resulting yellow-green solid was rinsed with cold methanol (2 × 25 mL) and dried under vacuum for 3 days to yield 4.5 g (72%). The solid was stored wrapped in foil to avoid photodegradation. ^1^H NMR (δ, 400 MHz, CDCl_3_): 8.19 (s, 1H), 7.51 (dd, *J* = 7.4, 1.6 Hz, 1H), 7.00 (dd, *J* = 7.9, 1.7 Hz, 1H), 6.76 (t, *J* = 7.6 Hz, 1H), 5.03 (s, 1H), 4.86 (t, *J* = 10.3 Hz, 1H), 4.19 (t, *J* = 13.3 Hz, 1H), 3.91 (d, *J* = 12.4 Hz, 1H), 3.77 (dd, *J* = 4.1, 4.8 Hz, 1H), 3.62 (s, 1H), 3.46 (s, 1H), 3.23 (s, 1H), 3.12 (s, 1H), 2.86 (dd, *J* = 13.3, 8.7 Hz, 1H), 1.41 (s, 9H)**.** ^51^V NMR (δ, 105 MHz, CDCl_3_): -543.7. UV-Vis (λ_max_/nm, CHCl_3_): 287. IR (cm^−1^, diamond ATR): 3379 (NH), 3156 (Ar CH), 2953 (alkane CH), 2924 (alkane CH), 2868 (alkane CH), 1622 (imine C=N), 945 (V=O).

*[VO(3-tBuHSHED)(TIPCAT)]* 3,4,6-tri-*iso*-propylcatechol (0.11 g, 0.44 mmol) was added to a solution of [VO_2_(3-tBuHSHED)] (0.15 g, 0.44 mmol) and the mixture was stirred in acetone (50 mL) for 24 h under an argon atmosphere. The reaction was wrapped in tinfoil to prevent unwanted photodegradation. After 24 h, the reaction mixture was filtered, and the filtrate was concentrated to dryness under reduced pressure. The crude purple solid was redissolved in a minimal amount of acetone and hexanes (60 mL) were added. The solution was allowed to stand at −20 °C for 3 days before being vacuum-filtered and rinsed with cold hexanes to yield 0.18 g (81%) as a purple solid. ^1^H NMR (δ, 400 MHz, DMSO-*d_6_*): 8.63 (s, 1H), 7.37–7.20 (m, 2H), 6.60 (t, *J* = 8.2 Hz, 1H), 6.44–5.86 (m, 1H), 4.84 (t, *J* = 5.1 Hz, 1H), 4.48–4.35 (m, 1H), 4.23–4.11 (m, 1H), 4.03–3.90 (m, 1H), 3.85–3.61 (m, 1H), 3.60–3.46 (m, 1H), 3.39–3.22 (m, 1H), 3.20–2.76 (m, 2H), 2.42 (d, *J* = 6.2 Hz, 1H), 1.36 (d, *J* = 6.8 Hz, 2H), 1.25–0.96 (m, 21H), 0.93–0.80 (m, 2H), 0.65 (d, *J* = 6.8 Hz, 1H). ^51^V NMR (δ, 105 MHz, DMSO-*d_6_*) 429, 404, 387. UV-Vis-NIR (λ_max_/nm, CHCl_3_): 410, 581, 903. IR (cm^−1^, diamond ATR): 3485 (NH), 3215 (Ar H), 2958 (alkane CH), 2867 (alkane CH), 1622 cm (imine C=N), 922 (V=O). HRMS (ESI) calc.: 565.28409 [M+H]; found: 565.28408 [M+H].

*[VO(HSHED)(TIPCAT)]* 3,4,6-tri-*iso*-propyl catechol (0.236 g, 1.00 mmol) was added to a solution of [VO_2_(HSHED)] (0.290 g, 1.00 mmol) in acetone (100 mL) and the mixture was allowed to stir for 24 h under an argon atmosphere. After 24 h, the mixture was filtered, and the filtrate was concentrated to dryness under reduced pressure. The purple solid was dissolved in a minimum amount of acetone and hexanes (100 mL) were added. The solution was placed in a −20 °C freezer for 1–3 days before it was filtered and rinsed with cold hexanes (25 mL). The purple crystalline product was dried under high vacuum for 1–2 days to yield 0.350 g (68.8%) of [VO(HSHED)(TIPCAT)] as a purple solid. ^1^H NMR (δ, 400 MHz, *d_6_*-DMSO): 8.74 (s, 1H), 7.49 (d, *J* = 7.5 Hz, 1H), 7.40 (t, *J* = 7.4 Hz, 1H), 6.68 (t, *J* = 8.6 Hz, 1H), 6.61 (d, *J* = 8.3 Hz, 1H), 5.97 (s, 1H), 4.86 (t, *J* = 5.1 Hz, 1H), 4.27–4.11 (m, 3H), 4.06–3.97 (m, 1H), 3.97–3.87 (m, 1H), 3.79 (s, 1H), 3.70–3.59 (m, 1H), 3.59–3.44 (m, 2H), 3.20–3.04 (m, 1H), 2.94–2.76 (m, 1H), 2.38 (d, *J* = 6.5 Hz, 1H), 1.22–1.04 (m, 18H). ^51^V NMR (δ, 105 MHz, *d_6_*-DMSO): 418.48. UV-Vis-NIR (λ_max_/nm, CHCl3): 392, 556, 909. IR (cm^−1^, diamond ATR): 3311 (NH), 3249 (NH), 2964 (alkane CH), 2950 (alkane CH), 2927 (alkane CH), 2865 (alkane CH), 1633 (imine C=N). HRMS (ESI) calc.: 509.22148 [M+H]; found: 509.22134 [M+H].

*[VO(3-tBuHSHED)(DIPCAT)]* 3,5-di-*iso*-propyl catechol (0.450 g, 2.32 mmol) was added to a solution of [VO_2_(3-tBuHSHED)] (0.79 g, 2.3 mmol) and the mixture was stirred in CH_2_Cl_2_ (230 mL) for 24 h under an argon atmosphere. The reaction was wrapped in tinfoil to prevent unwanted photodegradation. After 24 h, the reaction mixture was filtered, and the filtrate was concentrated to dryness under reduced pressure. The filtrate was concentrated to dryness under reduced pressure. The resulting purple solid was dissolved in a minimum amount of CH_2_Cl_2_ and then 200 mL of hexanes were added. The solution was allowed to stand at −20 °C for 3 days. The dark blue to purple microcrystalline product was filtered and rinsed with cold hexanes (50 mL) to yield 0.88 g (73%). ^1^H NMR (δ, 400 MHz, CDCl_3_): 8.34 (s, 1H), 7.40 (d, *J* = 7.5 Hz, 1H), 7.18 (d, *J* = 7.7 Hz, 1H), 6.69 (t, *J* = 7.9 Hz, 1H)., 6.24 (s, 1H), 6.08 (s, 1H), 4.32 (s, 1H), 4.19 (s, 1H), 4.00 (s, 2H), 3.87 (s, 1H), 3.67 (s, 1H), 3.43 (s, 1H), 3.33 (s, 1H), 3.15 (s, 1H), 2.91–2.80 (m, 1H), 1.25 (m, 6H), 1.21 (s, 9H), 1.18–1.09 (m, 6H). ^51^V NMR (δ, 105 MHz, CDCl_3_): 464, 338.4. UV-Vis-NIR (λ_max_/nm, CHCl_3_): 572, 841. IR (cm^−1^, diamond ATR): 3675 (OH), 3248 (NH), 2957 (alkane CH), 1627 (imine C=N).

*[VO(3-tBuHSHED)(DTB)]* 3,5-di-*tert*-butylcatechol (0.134 g, 0.602 mmol) was added to a solution of [VO_2_(3tBu-HSHED)] (0.208 g, 0.602 mmol) and the mixture was stirred in CHCl_3_ (25 mL) for 24 h under an argon atmosphere. The reaction was wrapped in tinfoil to prevent unwanted photodegradation and left to stir at ambient temperature under argon for 24 h. The resulting mixture was vacuum-filtered and the filtrate was concentrated to dryness under reduced pressure. The purple residue was dissolved in a minimum amount of CH_2_Cl_2_ and then hexanes (75 mL) were added. The flask was placed in a −20 °C freezer for 1–3 days before being filtered and rinsed with cold hexanes (25 mL). The resulting dark purple solid was dried under vacuum for 2 days to yield 0.158 g (49%). ^1^H NMR (δ, 400 MHz, CDCl_3_): 8.34 (s, 1H), 7.40 (d, *J* = 7.5 Hz, 1H), 7.17 (d, *J* = 7.8 Hz, 1H), 6.69 (t, *J* = 7.7 Hz, 1H), 6.39 (s, 1H), 6.31 (s, 1H), 4.27 (s, 1H), 4.14 (m, 2H), 3.96 (m, 2H), 3.65 (m, 1H), 3.42 (m, 2H), 3.09 (m, 1H), 2.65 (m, 1H), 1.44 (s, 9H), 1.24 (d, *J* = 6.7 Hz, 18H). ^51^V NMR (δ, 105 MHz, CDCl_3_): 562.35, 480.96, 354.94. UV-Vis-NIR (λ_max_/nm, CHCl_3_): 565, 869. IR (cm^−1^, diamond ATR): 3249 (NH), 2954 (alkane CH), 2904 (alkane CH), 2867 (alkane CH), 1630 (imine C=N). HRMS (ESI) calc.: 573.25038 [M+Na]; found: 573.25115 [M+Na].

### 4.2. NMR Spectroscopy

The complexes were characterized using ^51^V NMR spectroscopy recorded on a Bruker model AVANCE Neo400 spectrometer equipped with a BBFO smart probe and an automated tuning module operating at 105.2 MHz. The ^51^V NMR spectra were acquired with a spectral window commonly ranging from −600 to 800 ppm, 4096 scans, a 90° pulse, an acquisition time of 0.08 s, and a 0.01 s relaxation delay. One-dimensional ^51^V NMR studies were externally referenced against [VO_2_(HSHED)] at −529 ppm in DMSO and reported against VOCl_3_ at 0 ppm. Moreover, 1D and 2D ^1^H NMR studies were carried out in organic solvents using the same Bruker model spectrometer operating at 400 MHz at ambient temperature and routine parameters. Chemical shift values (δ) are referenced against tetramethylsilane (TMS) using the internal solvent peaks as internal standards. ^1^H-^1^H 2D COSY and NOESY NMR spectra in organic solutions were run overnight and recorded within 6 h of sample preparation. Two-dimensional NMR studies were carried out on the same Bruker model spectrometer at 400 MHz. A routine COSY pulse sequence provided by Bruker software (Topspin 4.1.3) was used. A standard NOESY pulse sequence was used with a 2.0 s relaxation delay and a 500 ms mixing time.

### 4.3. FTIR Spectroscopy

The spectra were recorded within the range of 600–4000 cm^−1^ using a Nicolet iS-50 FTIR spectrometer (Thermo Fisher Scientific, USA) with ATR crystals (diamond, ZnSe, and germanium). All samples were characterized on a diamond crystal and the spectra collected were an average of 32 scans of the sample. FTIR spectra can be found in the Supplementary Material (Figures S21–S29).

### 4.4. Mass Spectrometry

High-resolution positive-ion ESI-MS was performed on a Bruker Solarix 2XR 7T Fourier transform ion cyclotron resonance mass spectrometer (Bruker, Bremen, Germany). The solutions of the V(V) complexes (~0.1 mM) were prepared fresh in HPLC-grade acetonitrile and were injected via a syringe at 120 mL h^−1^. The transient length was 2 M and acquired in 2 ω mode with the Fourier transform collected in adsorption mode. The instrument was externally calibrated prior to analysis from 300 to 2000 *m*/*z*. Processing of the low- and high-resolution mass spectra was performed using Bruker Compass Data Analysis 5.0 software. High-resolution MS spectra can be found in the Supplementary Material (Figures S30–S35).

### 4.5. Stability Studies of V(V) Complexes Under Cell Culture Conditions

The decomposition of the V(V) complexes (0.10 mM) in cell culture medium at 310 K was followed by electronic absorption (UV-Vis-NIR) spectroscopy [37]. The medium used was Dulbecco’s modified Eagle medium (DMEM) without phenol red (Thermo Fisher Scientific Cat. No. 31053-028) that was fully supplemented according to the conditions of cell assays (see below) and also contained 10 mM HEPES 2-(4-(2-hydroxyethyl)piperazin-1-yl)-ethanesulfonic acid to maintain pH 7.4 outside of a 5% CO_2_ incubator [37,56]. Freshly prepared stock solutions of the V(V) complexes (10 μL of 10 mM solutions in DMSO) were added to 990 μL of pre-warmed medium, and time-dependent spectra (300–1020 nm with 0.5 nm interval) were collected on a Specord S600 diode-array spectrometer (Analytic Jena, Germany) that was equipped with a Peltier temperature controller. The spectra of the complexes (0.10 mM) in DMSO at 295 K were taken for comparison. Plotting and analysis of the spectra were performed using Pro-Kineticist global kinetic analysis software (2013 version, Applied Photophysics, Leatherhead, UK) and Origin Pro software (2022 version, OriginLab, Northampton, MA, USA).

### 4.6. Cell Culture and Viability Assays

The following human cell lines were purchased from the American Type Culture Collection: T98G (glioblastoma multiforme, CRL-1690) and SVG p12 (immortalized non-cancer brain astroglia, CRL-8621). The cells were cultured for up to ten passages in Advanced DMEM (Thermo Fisher Scientific, Melbourne, Australia, Cat. No. 12491-015), supplemented with L-glutamine (2.0 mM), antibiotic-antimycotic mixture (100 U mL^−1^ penicillin, 100 mg mL^−1^ streptomycin, and 0.25 mg mL^−1^ amphotericin B), and fetal calf serum (FCS, heat-inactivated; 2.0% vol) [13,37,66,75]. For viability experiments, the cells were seeded in 96-well plates at an initial density of 1.0 × 10^3^ (T98G) or 3.0 × 10^3^ (SVG p12) viable cells per well in 100 μL medium and left to attach overnight.

For all cell assays, freshly prepared stock solutions of the V(V) complexes (10 mM in DMSO) were used. The solutions were further diluted so that all cell treatments contained 1.0% (vol.) of DMSO, which was low enough to not affect cell growth during assays. The same concentration of DMSO was used in the control experiments. Stock solutions of Na_3_VO_4_ (10 mM in MilliQ H_2_O) [13] and cisplatin (1.0 mM in phosphate-buffered saline) [88] were prepared on the day of the experiments. Stock solutions of the treatment complexes were diluted with fully supplemented cell culture media to the required final concentrations, and the resultant media were added to the cells within 1 min (for the fresh solutions) or left in cell culture incubator (310 K, 5% CO_2_) for 24 h prior to cell treatments (for the aged solutions) [37]. The treatment complexes were applied in a series of nine two-fold dilutions, starting from 100 μM V, plus the vehicle control. Each treatment was composed of six replicate wells with cells and two background wells without cells but containing the same compounds. After the addition of the treatment complexes, the plates were incubated for 72 h at 310 K and 5% CO_2_, then the treatment medium was removed and replaced with freshly prepared solution of MTT reagent [1-(4,5-dimethylthiazol-2-yl)-3,5-diphenylformazan, Sigma M5655] (1.0 mg/mL in complete medium), and incubation was continued for 4–6 h. The medium was then removed, the blue formazan crystals were dissolved in 0.10 mL per well of DMSO, and the absorbance at 600 nm was measured with a Victor V3 plate reader. Origin Pro software (2022 version) was used for fitting the experimental data and calculations and IC_50_ value determination. For all assays, consistent results were obtained in at least two independent experiments using passages of cells and varying stock solutions of the treatment complexes [13,37,66,75].

## 5. Conclusions

In summary, we explored the desirable properties of drugs designed for intratumoral administration for glioblastoma T98G cells, which included the ability to rapidly react with tumor tissue while forming non-toxic products that pose minimal risk to normal tissue upon diffusion. Among the newly synthesized V(V) complexes reported in this study, [VO(3-tBuHSHED)(TIPCAT)] emerged as a promising candidate for glioblastoma treatment via intratumoral injections. The stability of this compound was superior to our current lead, [VO(HSHED)(DTB)]. The higher stability of [VO(3-tBuHSHED)(TIPCAT)] was likely to due to a combination of steric hindrance and hydrophobicity, which was inferred by the TIPCAT ligand and a more hydrophobic Schiff base scaffold and, thus, verified the hypothesis that increasing the stability of the compounds would lead to compounds with desirable properties. These findings highlight the potential of tailored ligand design in advancing intratumoral therapeutic strategies for glioblastoma.

## 6. Patents

Debbie C. Crans, Peter A. Lay, Heide A. Murakami, Aviva Levina, Kateryna Kostenkova, Andrew C. Bates, John F. Manganaro, Josef Grundy, Skyler A. Markham, Kameron L. Klugh. Vanadium Compounds and Methods of Making and Use Thereof, 10975-043US1, 2023.

## Figures and Tables

**Table 1:** Current and recent clinical trials of ITI and related techniques using metal-based drugs ^a,b^.

Trial Number ^c^	Drug (and Treatment Method)	Cancer Type	Phase	Participants
NCT04311762 ^d^ NCT04809103 ^d^ NCT00379665 ^d^	Cisplatin (ITI)	stage IV lung non-small cell lung carcinoma, non-small cell lung	I I II	9 10 25
NCT05644249	Cisplatin (PIPAC)	gastric	n/s	37
NCT05200650 ^d^	Cisplatin-loaded gel (ITI)	head and neck	II	20
NCT04811703 NCT03875144 NCT02735928 ^d^ NCT04065139 NCT04047004 ^d^ NCT06295094 NCT05303714 NCT04779385 ^d^ NCT01809379 ^d^ NCT01854255 ^d^	Cisplatin + doxorubicin (PIPAC)	ovarian peritoneal mesothelioma ovarian gastric adenocarcinoma gastric adenocarcinoma gastric peritoneal carcinomatosis peritoneal carcinomatosis recurrent ovarian peritoneal and gastric	I II n/s II I II III n/s II II	15 66 40 66 20 264 98 50 69 35
NCT04000906	Cisplatin + nab-paclitaxel (PIPAC)	peritoneal carcinomatosis	I	18
NCT06430515	Cisplatin, oxaliplatin	advanced solid	n/s	200
NCT02604784 ^d^	Cisplatin, oxaliplatin, doxorubicin (PIPAC)	peritoneal carcinomatosis	I, II	105
NCT04781725 ^d^ NCT06358573 NCT03058289 ^d^	INT230-6 (cisplatin + vinblastine) (ITI)	breast cancer triple-negative breast cancer skin tumors and body tumors	II II I, II	90 54 110
NCT01644955 ^d^	Carboplatin (CED)	glioblastoma recurrent high-grade gliomas	I	10
NCT04391049	Carboplatin and paclitaxel (ITI)	esophageal and gastroesophageal	I	16
NCT04541108	Carboplatin (various formulations) (ITI)	intratumoral microdosing master protocol	n/s	36
NCT04913662 ^d^ NCT06091683 NCT03280511 NCT03172416 NCT02604784 ^d^ NCT04122885 ^d^ NCT03246321 ^d^	Oxaliplatin (PIPAC)	peritoneal metastases peritoneal metastases from colorectal colon gastric peritoneal, ovarian, gastric, and colorectal ovarian, gastric, and colorectal colorectal	I II I I, II n/s II	18 10 60 21 105 60 20
NCT03294252 ^d^	Oxaliplatin + L-Folinic acid (PIPAC)	nonresectable peritoneal metastases of digestive	II	50

^a^ Information was obtained from www.clinicaltrials.gov, accessed 19 July 2024. ^b^ Abbreviations: ITI is intratumoral injection; PIPAC is pressurized intraperitoneal aerosolized chemotherapy (technique of direct delivery of cytotoxic drugs into tumors of digestive systems); and CED is convention-enhanced delivery (injections into skull to overcome blood–brain barrier for treatment of brain tumors). ^c^ NCT Number: National Clinical Trial number is identifier assigned to registered studies by ClinicalTrials.gov. ^d^ Trial completed.

**Table 2:** Complex lifetimes and antiproliferative activities of V(V) complexes, ligands, vanadate, and cisplatin.

Compound	*t*_1/2_^ a^	IC_50_, (T98G) ^b^	IC_50_ (SVG p12) ^b^
[VO(3-tBuHSHED)(DTB)]	1.6 h	1.5 ± 0.3 (A); 16 ± 2 (B)	1.2 ± 0.3 (A); 22 ± 1 (B)
[VO(HSHED)(DTB)]	31.5 s	2.3 ± 0.2 (A); 20 ± 2 (B)	3.6 ± 0.2 (A); 24 ± 1 (B)
[VO(3-tBuHSHED)(CAT)]	53 s	12 ± 1.5 (A); 5.2 ± 0.6 (B)	ND
[VO(HSHED)(CAT)]	8.0 s	11 ± 1 (A); 8.0 ± 0.6 (B)	ND
[VO(3-tBuHSHED)(TIPCAT)]	4.5 h	1.6 ± 0.1 (A); 33 ± 3 (B)	3.4 ± 0.7 (A); 46 ± 5 (B)
[VO(3-tBuHSHED)(DIPCAT)]	14.8 min	9 ± 1 (A); 47 ± 4 (B)	7 ± 2 (A); 40 ± 4 (B)
[VO(HSHED)(TIPCAT)]	93 s	14 ± 1 (A); 55 ± 4 (B)	17 ± 1 (A); 55 ± 5 (B)
DTBH_2_	ND ^c^	26 ± 3 (B)	ND
TIPCATH_2_	ND	54 ± 7 (B)	ND
DIPCATH_2_	ND	54 ± 8 (B)	ND
H_2_SHED	ND	>100 (B)	ND
3-tBuH_2_SHED	ND	70 ± 5 (B)	ND
Na_3_VO_4_	ND	40 ± 3 (B)	48 ± 4 (B)
cisplatin	ND	31 ± 1 (A)	6.1 ± 0.4 (A)

^a^ Half-life times of complexes in fully supplemented cell culture medium at 310 K, determined by global kinetic analysis (Figures S36–S42 in Supplementary Materials). ^b^ Concentrations of compounds (μM) that cause 50% decrease in cell viability after 72 h incubation. Freshly prepared solutions of compounds (0–100 μM) were added to cells within 1 min (A, fresh solutions), or pre-incubated with medium for 24 h at 310 K before addition to cells (B, aged solutions). Typical concentration–viability curves are shown in Figures S43–S45, Supplementary Materials. ^c^ Values not determined.

## Data Availability

The information is provided in the Supplementary Materials.

## Supplementary Materials

The following supporting information can be downloaded at: https://www.mdpi.com/article/10.3390/ijms26030994/s1.
