# Supplementary material for: Optimizing Therapeutics for Intratumoral Cancer Treatments: Antiproliferative Vanadium Complexes in Glioblastoma
Source: Int J Mol Sci. 2025 Jan 24;26(3):994. doi: 10.3390/ijms26030994 (PMC11817060; doi:10.3390/ijms26030994)
Supplement: Supplementary file 1 [file ijms-26-00994-s001.zip › ijms-3393661-supplementary.pdf]

Supplementary Materials for:

Optimizing Therapeutics for Intratumoral Cancer Treatments: Antiproliferative Vanadium Complexes in Glioblastoma

Andrew C. Bates[1], Kameron L. Klugh[1], Anna O. Galaeva[1], Raley A. Patch[1], John F. Manganaro[1], Skyler A. Markham[1], Emma Scurek[1], Aviva Levina[2], Peter A. Lay[2,*], Debbie C. Crans[1,3,*]

(1)Department of Chemistry, Colorado State University, Fort Collins, Colorado, 80526,
(2) School of Chemistry, The University of Sydney, Sydney 2006 NSW, Australia
(3) Cell and Molecular Biology Program, Colorado State University, Fort Collins, Colorado.

Corresponding authors: Debbie C. Crans (Debbie.Crans@colostate.edu); Peter A. Lay (peter.lay@sydney.edu.au)

International Journal of Molecular Sciences

I. **Experimental details**
3,4,6-tri-*iso*-propyl catechol
3,5-di-*iso*-propyl catechol
[VO_2(3-tBuHSHED)]
[VO(3-tBuHSHED)(CAT)]
[VO(3-tBuHSHED)(DIPCAT)]
[VO(3-tBuHSHED)(TIPCAT)]
[VO(3-tBuHSHED)(DTB)]
[VO(HSHED)(TIPCAT)]
[VO(HSHED)(DIPCAT)]

II. **List of clinical trials** of intratumoral injections and related techniques with Pt-based drugs (**Table S1**)

III. **Lipophilicity calculations (Log P)**
Table S2 Calculated log P values for catechol ligands
Table S3 Estimated log P values for V-catechol complexes

IV. **1D NMR Characterization**
Figure S1 ^{1}H NMR of 3,4,6-tri-i*so*-propyl catechol
Figure S2 ^{1}H NMR of 3,5-di-*iso*-propyl catechol
Figure S3 ^{1}H NMR of [VO_2(3-tBuHSHED)]
Figure S4 ^{51}V NMR of [VO_2(3-tBuHSHED)]
Figure S5 ^{1}H NMR of [VO(3-tBuHSHED)(CAT)]
Figure S6 ^{51}V NMR of [VO(3-tBuHSHED)(CAT)]
Figure S7 ^{1}H NMR of [VO(3-tBuHSHED)(DIPCAT)]
Figure S8 ^{51}V NMR of [VO(3-tBuHSHED)(DIPCAT)]
Figure S9 ^{1}H NMR of [VO(3-tBuHSHED)(TIPCAT)]
Figure S10 ^{51}V NMR of [VO(3-tBuHSHED)(TIPCAT)]
Figure S11 ^{1}H NMR of [VO(HSHED)(TIPCAT)]
Figure S12 ^{51}V NMR of [VO(HSHED)(TIPCAT)]
Figure S13 ^{1}H NMR of [VO(HSHED)(DIPCAT)]
Figure S14 ^{51}V NMR of [VO(HSHED)(DIPCAT)]
Figure S15 ^{1}H NMR of [VO(3-tBuHSHED)(DTB)]
Figure S16 ^{51}V NMR of [VO(3-tBuHSHED)(DTB)]
Figure S17 Stacked ^{51}V NMR of the [VO(3-tBuHSHED)(X)] series

V. **2D NMR Characterization**
Figure S18 [VO(HSHED)(TIPCAT)]
Figure S19 [VO(3-tBuHSHED)(DTB)]

Figure S20 [VO(3-tBuHSHED)(TIPCAT)]

VI. FTIR Characterization

Figure S21 FTIR of 3,4,6-tri-*iso*-propyl catechol
Figure S22 FTIR of 3,5-di-*iso*-propyl catechol
Figure S23 FTIR of [VO_2(3-tBuHSHED)]
Figure S24 FTIR of [VO(3-tBuHSHED)(CAT)]
Figure S25 FTIR of [VO(3-tBuHSHED)(DIPCAT)]
Figure S26 FTIR of [VO(3-tBuHSHED)(TIPCAT)]
Figure S27 FTIR of [VO(HSHED)(TIPCAT)]
Figure S28 FTIR of [VO(HSHED)(DIPCAT)]
Figure S29 FTIR of [VO(3-tBuHSHED)(DTB)]

VII. Mass Spectrometry

Figure S30 Experimental (top) and simulated (bottom) HRMS spectra of [VO(3tBuHSHED)(CAT)]

Figure S31 Experimental (top) and simulated (bottom) HRMS spectra of [VO(3-tBuHSHED)(DTB)]

Figure S32 Experimental (top) and simulated (bottom) HRMS spectra of [VO(3-tBuHSHED)(TIPCAT)]

Figure S3 Experimental (top) and simulated (bottom) HRMS spectra of [VO(HSHED)(CAT)]

Figure S34 Experimental (top) and simulated (bottom) HRMS spectra of [VO(HSHED)(DTB)]

Figure S35 Experimental (top) and simulated (bottom) HRMS spectra of [VO(HSHED)(TIPCAT)]

VIII. Global Kinetic Analysis

Figure S36 (A-D) [VO(3-tBuHSHED)(DTB)]
Figure S37 (A-D) [VO(HSHED)(DTB)]
Figure S38 (A-D) [VO(3-tBuHSHED)(CAT)]
Figure S39 (A-D) [VO(HSHED)(CAT)]

Figure S40 (A-D) [VO(3-tBuHSHED)(TIPCAT)]
Figure S41 (A-D) [VO(3-tBuHSHED)(DIPCAT)]
Figure S42 (A-D) [VO(HSHED)(TIPCAT)]

II. Cell Viability

Figure S43 (A-F)
A [VO(3-tBuHSHED)(DTB)] in T98G cells
B [VO(3-tBuHSHED)(DTB)] in SVG p12 cells
C [VO(HSHED)(DTB)] in T98G cells
D [VO(HSHED)(DTB)] in SVG p12 cells
E [VO(3-tBuHSHED)(CAT)] in T98G cells
F [VO(HSHED)(CAT)] in T98G cells

Figure S44 (A-F)
A [VO(3-tBuHSHED)(TIPCAT)] in T98G cells
B [VO(3-tBuHSHED)(TIPCAT)] in SVG p12 cells
C [VO(3-tBuHSHED)(DIPCAT)] in T98G cells
D [VO(3-tBuHSHED)(DIPCAT)] in SVG p12 cells
E [VO(HSHED)(TIPCAT)] in T98G cells
F [VO(HSHED)(TIPCAT)] in SVG p12 cells

Figure S45 (A-D)
A Catechol ligands in T98G cells
B Schiff base ligands in T98G cells
C Na_3VO_4 in T98G or SVG p12 cells
D Cisplatin in T98G or SVG p12 cells

I. Experimental details

General considerations. Solvents used in synthesis were degassed prior to use with argon for 15 minutes and all reactions were conducted under argon atmospheres. [VO_2(HSHED)] was prepared as previously reported [1] and [VO_2(3-tBuHSHED)] was prepared using a slightly modified procedure. Catechol ligands **1** and **2** were prepared with slight modifications to the previous report [2,3]. Complexes containing catechol ligands exhibit multiple geometric isomers in solution, with their distribution being

influenced by the solvent used. Only the major isomers are reported in the ^{51}V NMR chemical shift data.

3,4,6-tri-*iso*-propyl catechol (1) preparation and characterization data found in main text.

3,5-di-*iso*-propyl catechol (2) was isolated as a byproduct of the synthesis of **1** to yield 0.58 g (15%) of 3,5-di-*iso*-propyl catechol as a pale orange solid. The product was further purified by recrystallization from petroleum ether to yield colorless crystals. ^{1}H NMR (δ, 400 MHz, $CDCl_3$): 6.63 (d, *J* = 2.1 Hz, 1H), 6.60 (d, *J* = 2.1 Hz, 1H), 4.97 (s, 1H), 4.88 (s, 1H), 3.17 (sept, *J* = 6.9 Hz, 1H), 2.79 (sept, *J* = 7.0 Hz, 1H), 1.26 (d, *J* = 6.8 Hz, 6H), 1.20 (d, *J* = 6.9 Hz, 6H). IR (cm^{-1}, diamond ATR): 3282 (OH), 2957 (alkane CH), 2924 (alkane CH), 2868 (alkane CH).

VO_2(3-tBuHSHED)] preparation and characterization data found in main text.

[VO(3-tBuHSHED)(CAT)] Catechol (0.078 g, 0.722 mmol) was added to a solution of [VO_2(3-tBu-HSHED)] (0.250 g, 0.722 mmol) and stirred in acetone (70 mL) for 24 hours under an argon atmosphere. The reaction was wrapped in tinfoil to prevent unwanted photodegradation. After 24 hours, the reaction mixture was filtered and the filtrate was concentrated to dryness under reduced pressure. The purple residue was dissolved in a minimum amount of acetone and then hexanes (75 mL) was added. The flask was placed in a -20 °C freezer for 1-3 days before being filtered and rinsed with cold hexanes (25 mL). The resulting dark purple solid was dried under vacuum for 2 days to yield 0.18 g (57%). ^{1}H NMR (δ, 400 MHz, $CDCl_3$): 8.42 (s, 1H), 7.46 (d, *J* = 7.5 Hz, 1H), 7.23 (d, *J* = 7.5 Hz, 1H), 6.79 (dt, *J* = 15.1, 7.6 Hz, 2H), 6.65 (d, *J* = 8.1 Hz, 1H), 6.51 (d, *J* = 8.3 Hz, 1H), 6.46 (t, *J* = 7.4 Hz, 1H), 4.48 (s, 1H), 4.27 – 4.13 (m, 1H), 4.06 – 3.90 (m, 2H), 3.90 – 3.80 (m, 1H), 3.77 – 3.65 (m, 1H), 3.50 – 3.42 (m, 1H), 3.38 (s, 1H), 3.15 – 2.99 (m, 1H), 1.25 (s, 9H). ^{51}V NMR (δ, 105 MHz, $CDCl_3$): 250.78, 124.22. UV-Vis-NIR (λ_{max}/nm, $CHCl_3$): 546, 864. IR (cm^{-1}, diamond ATR): 3251 (NH), 3051(Ar CH), 2952 (alkane CH), 2866 (alkane CH), 1628 (imine C=N), 945 (V=O). HRMS (ESI) calc.: 461.12517 [M+Na]; found: 461.12481 [M+Na].

[VO(3-tBuHSHED)(DIPCAT)] 3,5-di-*iso*-propyl catechol (0.450 g, 2.32 mmol) was added to a solution of [VO_2(3-tBuHSHED)] (0.79 g, 2.3 mmol) and stirred in CH_2Cl_2 (230 mL) for 24 hours under an argon atmosphere. The reaction was wrapped in tinfoil to prevent unwanted photodegradation. After 24 hours, the reaction mixture was filtered and the filtrate was concentrated to dryness under reduced pressure. The filtrate was concentrated to dryness under reduced pressure. The resulting purple solid was dissolved in a minimum amount of CH_2Cl_2 and then 200 mL of hexanes was added. The solution was allowed to stand at -20 °C for 3 days. The dark blue to purple

microcrystalline product was filtered and rinsed with cold hexanes (50 mL) to yield 0.88 g (73%). ^{1}H NMR (δ, 400 MHz, $CDCl_3$): 8.34 (s, 1H), 7.40 (d, *J* = 7.5 Hz, 1H), 7.18 (d, *J* = 7.7 Hz, 1H), 6.69 (t, *J* = 7.9 Hz, 1H). 6.24 (s, 1H), 6.08 (s, 1H), 4.32 (s, 1H), 4.19 (s, 1H), 4.00 (s, 2H), 3.87 (s, 1H), 3.67 (s, 1H), 3.43 (s, 1H), 3.33 (s, 1H), 3.15 (s, 1H), 2.91 – 2.80 (m, 1H), 1.25 (m, 6H), 1.21 (s, 9H), 1.18 – 1.09 (m, 6H). ^{51}V NMR (δ, 105 MHz, $CDCl_3$): 464, 338.4. UV-Vis-NIR (λ_{max}/nm, $CHCl_3$): 572, 841. IR (cm^{-1}, diamond ATR): 3675 (OH), 3248 (NH), 2957 (alkane CH), 1627 (imine C=N).

[VO(3-tBuHSHED)(TIPCAT)] preparation and characterization data found in main text.

[VO(3-tBuHSHED)(DTB)] 3,5-di-*tert*-butylcatechol (0.134 g, 0.602 mmol) was added to a solution of [VO_2(3tBu-HSHED)] (0.208 g, 0.602 mmol) and stirred in $CHCl_3$ (25 mL) for 24 hours under an argon atmosphere. The reaction was wrapped in tinfoil to prevent unwanted photodegradation and left to stir at ambient temperature under argon for 24 hours. The resulting mixture was vacuum filtered and the filtrate was concentrated to dryness under reduced pressure. The purple residue was dissolved in a minimum amount of CH_2Cl_2 and then hexanes (75 mL) was added. The flask was placed in a -20 °C freezer for 1-3 days before being filtered and rinsed with cold hexanes (25 mL). The resulting dark purple solid was dried under vacuum for 2 days to yield 0.158 g (49%). ^{1}H NMR (δ, 400 MHz, $CDCl_3$): 8.34 (s, 1H), 7.40 (d, J = 7.5 Hz, 1H), 7.17 (d, J = 7.8 Hz, 1H), 6.69 (t, J = 7.7 Hz, 1H), 6.39 (s, 1H), 6.31 (s, 1H), 4.27 (s, 1H), 4.14 (m, 2H), 3.96 (m, 2H), 3.65 (m, 1H), 3.42 (m, 2H), 3.09 (m, 1H), 2.65 (m, 1H), 1.44 (s, 9H), 1.24 (d, J = 6.7 Hz, 18H). ^{51}V NMR (δ, 105 MHz, $CDCl_3$): 562.35, 480.96, 354.94. UV-Vis-NIR (λ_{max}/nm, $CHCl_3$): 565, 869. IR (cm^{-1}, diamond ATR): 3249 (NH), 2954 (alkane CH), 2904 (alkane CH), 2867 (alkane CH), 1630 (imine C=N). HRMS (ESI) calc.: 573.25038 [M+Na]; found: 573.25115 [M+Na].

[VO(HSHED)(TIPCAT)] 3,4,6-tri-*iso*-propyl catechol (0.236 g, 1.00 mmol) was added to a solution of [VO_2(HSHED)] (0.290 g, 1.00 mmol) in acetone (100 mL) and allowed to stir for 24 hours under an argon atmosphere. After 24 hours the mixture was filtered and the filtrate was concentrated to dryness under reduced pressure. The purple solid was dissolved in a minimum amount of acetone and hexanes (100 mL) was added. The solution was placed in a -20 °C freezer for 1-3 days before it was filtered and rinsed with cold hexanes (25 mL). The purple crystalline product was dried under high vacuum for 1-2 days to yield 0.350 g (68.8%) of [VO(HSHED)(TIPCAT)] as a purple solid. ^{1}H NMR (δ, 400 MHz, *d*$_6$-DMSO): 8.74 (s, 1H), 7.49 (d, *J* = 7.5 Hz, 1H), 7.40 (t, *J* = 7.4 Hz, 1H), 6.68 (t, *J* = 8.6 Hz, 1H), 6.61 (d, *J* = 8.3 Hz, 1H), 5.97 (s, 1H), 4.86 (t, *J* = 5.1 Hz, 1H), 4.27 – 4.11 (m, 3H), 4.06 – 3.97 (m, 1H), 3.97 – 3.87 (m, 1H), 3.79 (s, 1H), 3.70 – 3.59 (m, 1H), 3.59 – 3.44 (m, 2H), 3.20 – 3.04 (m, 1H), 2.94 – 2.76 (m, 1H), 2.38 (d, *J* = 6.5 Hz, 1H), 1.22 – 1.04 (m, 18H). ^{51}V NMR (δ, 105 MHz, *d*$_6$-DMSO): 418.48. UV-Vis-NIR (λ_{max}/nm, $CHCl_3$): 392, 556, 909. IR (cm^{-1}, diamond ATR): 3311 (NH), 3249 (NH), 2964 (alkane CH), 2950 (alkane CH),

2927 (alkane CH), 2865 (alkane CH), 1633 (imine C=N). HRMS (ESI) calc.: 509.22148 [M+H]; found: 509.22134 [M+H].

[VO(HSHED)(DIPCAT)] 3,5-di-*iso*-propyl catechol (0.194 g, 1.00 mol) was added to a solution of [VO_2(HSHED)] (0.290 g, 1.00 mol) in degassed acetone (100 mL) and allowed to stir for 24 hours under an argon atmosphere. After 24 hours the reaction mixture was filtered, and the filtrate was concentrated under reduced pressure. The purple solid was dissolved in a minimum amount of acetone and hexanes (100 mL) was added. The flask was placed in a -20 °C freezer for 1-3 days before being filtered and rinsed with cold hexanes (25 mL). The resulting purple solid was collected and dried under high vacuum for 2 days to yield 0.125 g (27%). ^{1}H NMR (δ, 400 MHz, $CDCl_3$): 8.36 (s, 1H), 7.40 (t, *J* = 7.8 Hz, 1H), 7.30 (d, *J* = 7.7 Hz, 1H), 6.92 (d, *J* = 8.5 Hz, 1H), 6.74 (t, *J* = 7.6 Hz, 1H), 6.24 (s, 1H), 6.08 (s, 1H), 4.24 – 4.10 (m, 1H), 4.03 – 3.95 (m, 3H), 3.89 (s, 1H), 3.63 (s, 1H), 3.46 (m, 1H), 3.41 – 3.29 (m, 2H), 3.04 (m, 1H), 2.95 – 2.84 (m, 1H), 2.74 – 2.49 (m, 1H), 1.32 – 1.12 (m, 12H). ^{51}V NMR (δ, 105 MHz, $CDCl_3$): 512.65, 389.26. UV-Vis-NIR (λ_{max}/nm, $CHCl_3$): 391, 571, 871. IR (cm^{-1}, diamond ATR): 3390 (OH), 3251 (NH), 2956 (alkane CH), 2867 (alkane CH), 1627 (imine C=N).

II. List of Clinical Trials

Table S1. Current and recent clinical trials of ITI and related techniques using transition metals-based drugs.

Entry	Identifier No.[d]	Treatment [a]	Drug	Disease	Phase	Participants	Institution	Dates
1	NCT04311762	ITI	cisplatin	stage IV lung cancer	I	9	U. of Vermont, Burlington, VT, USA	02-2020–03-2022 [b]
2	NCT04809103	ITI	cisplatin	non-small cell lung cancer	I	10	U. of Vermont, Burlington, VT, USA	03-2021–09-2023 [b]
3	NCT04781725	ITI	INT230-6 (cisplatin + vinblastine)	breast cancer	II	90	The Ottawa Hosp. Res. Inst. and Canc. Cntr., Ontario, Canada	03-2021–03-2023 [b]
4	NCT05200650	ITI	cisplatin loaded gel	head and neck cancer	II	20	Hadassah Med. Cntr, Jerusalem, Israel	03-2022–11-2022 [b]
5	NCT06430515	ITI	cisplatin, oxaliplatin	advanced solid cancers	n/s	200	Wuxi People's Hosp., China	09-2023–12-2028 [c]
6	NCT00379665	ITI	cisplatin	carcinoma, non-small cell lung cancer	II	25	SW Regional Med. Center	10-2005–09-2012 [b]
7	NCT06358573	ITI	INT230-6 (cisplatin + vinblastine)	triple-negative breast cancer	II	54	Swiss Group for Clinical Cancer Research	06-2024–12-2029 [c]
8	NCT03058289	ITI	INT230-6 (cisplatin + vinblastine)	skin tumors and body tumors	I, II	110	Intensity Therapeutics	09-2017–02-2023 [b]
9	NCT05644249	PIPAC	cisplatin	gastric cancer	n/s	37	Natl. Cancer Inst., Lithuania	12-2022–10-2027 [c]
10	NCT04811703	PIPAC	cisplatin + doxorubicin	ovarian cancer	I	15	Hospices Civils de Lyon, Lille, France	07-2021–01-2025 [c]
11	NCT03875144	PIPAC	cisplatin + doxorubicin	peritoneal mesothelioma	II	66	Inst. du Cancer de Montpellier - Val d'Aurelle, France	10-2022–09-2028 [c]
12	NCT02735928	PIPAC	cisplatin + doxorubicin	ovarian cancer	n/s	40	Catholic U. of the Sacred Heart, Rome, Italy	01-2016–06-2022 [b]

13	NCT04065139	PIPAC	cisplatin + doxorubicin	gastric adenocarcinoma	II	66	Hosp. de Paris, France	06-2020–12-2024 [c]
14	NCT04047004	PIPAC	cisplatin + doxorubicin	gastric adenocarcinoma	I	20	Odense U. Hosp., Denmark	03-2003–10-2022 [b]
15	NCT02604784	PIPAC	cisplatin, oxaliplatin, doxorubicin	peritoneal carcinomatosis	I, II	105	Fondazione del Piemonte per l'Oncologia, Turin, Italy	10-2015–05-2020 [b]
16	NCT04000906	PIPAC	cisplatin + nab-paclitaxel	peritoneal carcinomatosis	I	18	U. Hosp., Geneva, Switzerland	11-2020–12-2024 [c]
17	NCT06295094	PIPAC	cisplatin + doxorubicin	gastric cancer	II	264	Odense U. Hosp., Denmark	09-2024–01-2028 [c]
18	NCT05303714	PIPAC	cisplatin + doxorubicin	peritoneal carcinomatosis	III	98	Azienda Ospedaliera U. Integrata Verona, Italy	03-2022–09-2028 [c]
19	NCT04779385	PIPAC	cisplatin + doxorubicin	peritoneal carcinomatosis	n/s	50	U. Hosp., Grenoble, France	11-2020–03-2021 [b]
20	NCT01809379	PIPAC	cisplatin + doxorubicin	recurrent ovarian cancer	II	69	Ruhr U. of Bochum, Germany	02-2013–09-2014 [b]
21	NCT01854255	PIPAC	cisplatin + doxorubicin	peritoneal cancer and gastric cancer	II	35	Ruhr U. of Bochum, Germany	11-2013–11-2016 [b]
22	NCT04391049	ITI	carboplatin and paclitaxel	esophageal and gastroesophageal cancer	I	16	NRG Oncology, US	06-2020–10-2025 [c]
23	NCT04541108	ITI	carboplatin (various formulations)	development of master protocol for intratumoral microdosing	n/s	36	Presage Biosciences (various locations in USA)	07- 2021–12-2031 [c]
24	NCT01317212	CED	carboplatin	glioblastoma	I	0	North Bristol NHS Trust, UK	05-2015–05-2018 [b]
25	NCT01644955	CED	carboplatin	glioblastoma	I	10	Ohio St. U. Comprehensive Canc. Cntr.	06-2012–12-2017 [b]
26	NCT01644955	CED	carboplatin	recurrent high-grade gliomas	I	10	Ohio St. U. Med. Ctr., Columbus, OH, USA	06-2012–12-2017 [b]

27	NCT03294252	PIPAC	oxaliplatin + L-Foliniac acid	nonresectable peritoneal metastases of digestive cancers	II	50	Centre Hospitalier Lon Sud, Puerre-Bénite, France	05-2017–06-2021 [b]
28	NCT04913662	PIPAC	oxaliplatin	peritoneal metastases	I	18	Seoul Natl. U. Bundang Hosp., Korea	04-2021–06- 2022 [b]
39	NCT06091683	PIPAC	oxaliplatin	Peritoneal metastases from colorectal cancer		10	Fondazione IRCCS Inst. Natl. dei Tumori, Milano	04-2022–12- 2025 [c]
30	NCT03280511	PIPAC	oxaliplatin	colon cancer	II	60	Odense U. Hosp., Denmark	12-2017–03 2027 [c]
31	NCT03172416	PIPAC	oxaliplatin	gastric cancer	I	21	Natl. U. Hospital, Singapore	04-2017–12-2024 [c]
32	NCT02604784	PIPAC	oxaliplatin	peritoneal cancer, ovarian cancer, gastric cancer, colorectal cancer	I, II	105	Fondazione del Piemonte per l'Oncologia	10-2015–05 2020 [b]
33	NCT04122885	PIPAC	oxaliplatin	ovarian, gastric, and colorectal cancers	n/s	60	U. Hosp. Tuebingen, Germany	10-2019–09-2020 [b]
34	NCT03246321	PIPAC	oxaliplatin	colorectal cancer	II	20	Catharina Ziekenhuis Eindhoven, Netherlands	10-2017–10-2019 [b]
35	NCT06048367	ITI	carbon nanoparticle-loaded iron [CNSI-Fe(II)]	pancreatic cancer	I	24	West China Hosp., Sichuan U.	10-2022–02-2024 [b]
36	NCT04316091	ITI	superparamagnetic Fe oxide nanoparticles (SPIONs)/spinning magnetic field (SMF)	osteosarcoma	I	60	Dept. of Ortho. Surg., Xiaoshan Chinese Med. Hosp., Hangzhou, China	09-2020–08-2023 [b]

[a] Abbreviations: ITI is intratumoral injection; PIPAC is pressurized intraperitoneal aerosolized chemotherapy (a technique of direct delivery of cytotoxic drugs into tumors of digestive systems); and CED is convention enhanced delivery (injections into the skull to overcome the blood-brain barrier for the treatment of brain tumors). [b] Trial completed. [c] Trial

ongoing. [d] NCT Number: National Clinical Trial number is an identifier assigned to registered studies by ClinicalTrials.gov (accessed July 19, 2024).

III. Log P values estimated with Chemicalize

Table S2. Structures of catechol ligands used in this study with calculated log P.

Catechol ligand	Log P
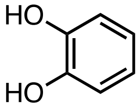 **pyrocatechol (CAT)**	**1.37**
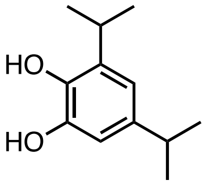 **3,5-di-*iso*-propylcatechol (DTB)**	**3.86**
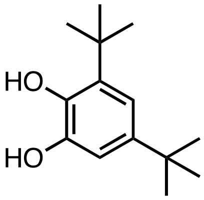 **3,5-di-*tert*-butylcatechol (DIPCAT)**	**4.46**
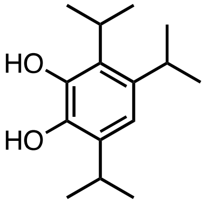 **3,4,6-tri-*iso*-propylcatechol (TIPCAT)**	**5.10**

Table S3. Log P values of the [VO(3-tBuHSHED)] and [VO(HSHED)] series of catechol complexes as estimated using Chemicalize software.

Complex 1	Log P
**[VO(HSHED)(CAT)]**	2.2
**[VO(HSHED)(DTB)]**	5.5
**[VO(HSHED)(DIPCAT)]**	4.6
**[VO(HSHED)(TIPCAT)]**	5.8
**[VO(3-tBuHSHED)(CAT)]**	3.8
**[VO(3-tBuHSHED)(DTB)]**	7.1
**[VO(3-tBuHSHED)(TIPCAT)]**	7.4
**[VO(3-tBuHSHED)(DIPCAT)]**	6.2
*Calculations required the removal of coordinate bonds between nitrogen atoms and V to generate log P values.	

Lipophilicity and *P* Values. The lipophilicity and partition coefficient (log P) of compounds is a critical indicator of membrane permeability and absorption and distribution of a drug. Due to the poor aqueous solubility of the reported compounds, Chemicalize software by ChemAxon was used to estimate the hydrophobicity of the complexes. It is important to note that this software is designed for organic molecules and not for metal complexes with coordinate bonds. Given that all complexes share similar coordinate bonds, this approach reflects their relative comparative hydrophobic differences as shown in Table S2 yielding very similar log P values between the complex containing the trisubstituted catecholate [VO(3-tBuHSHED)(TIPCAT)] with the complex containing the disubstituted catecholate [VO(3-tBuHSHED)(DTB)].

IV. 1D NMR Characterization

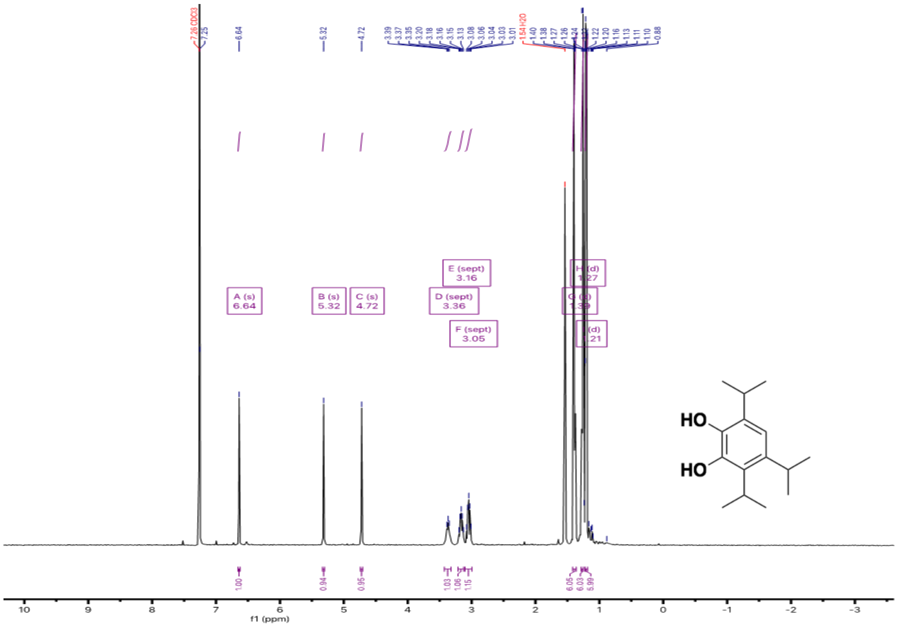

Figure S1 ^{1}H NMR of 3,4,6-tri-*iso*-propyl catechol in $CDCl_3$.

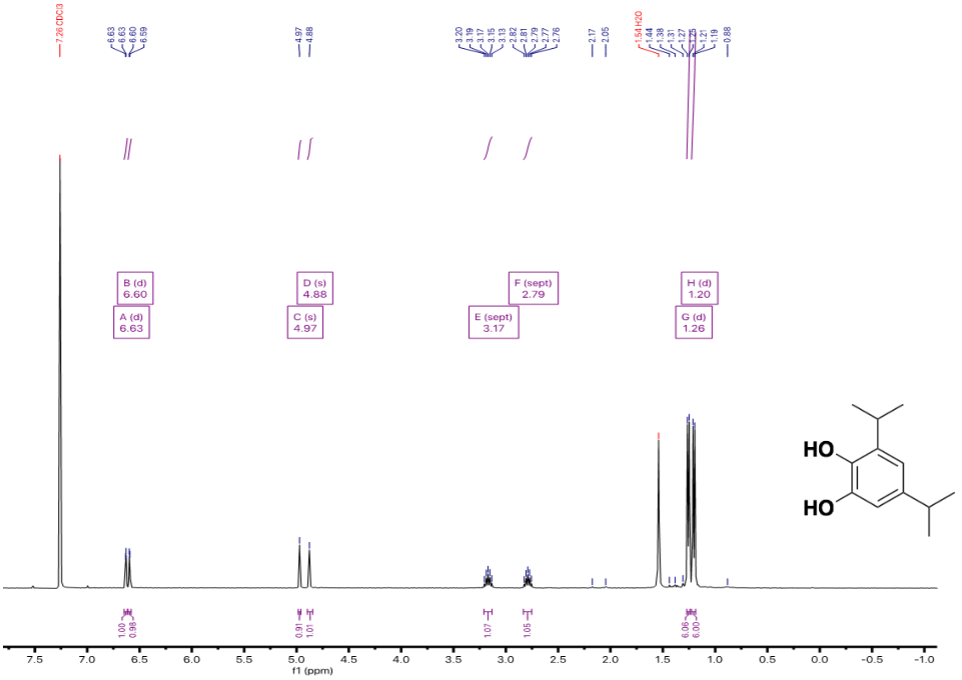

Figure S2 ^{1}H NMR of 3,5-di-*iso*-propyl catechol in $CDCl_3$.

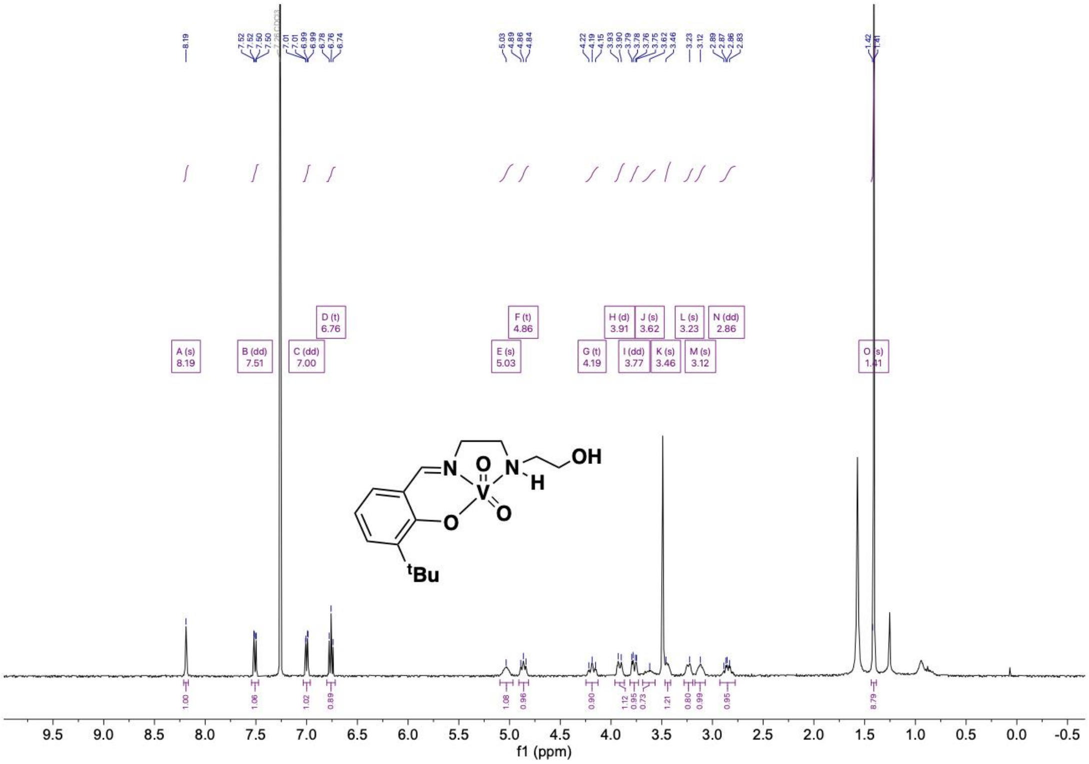

Figure S3 ^{1}H NMR of [VO_2(3-tBuHSHED)] in $CDCl_3$.

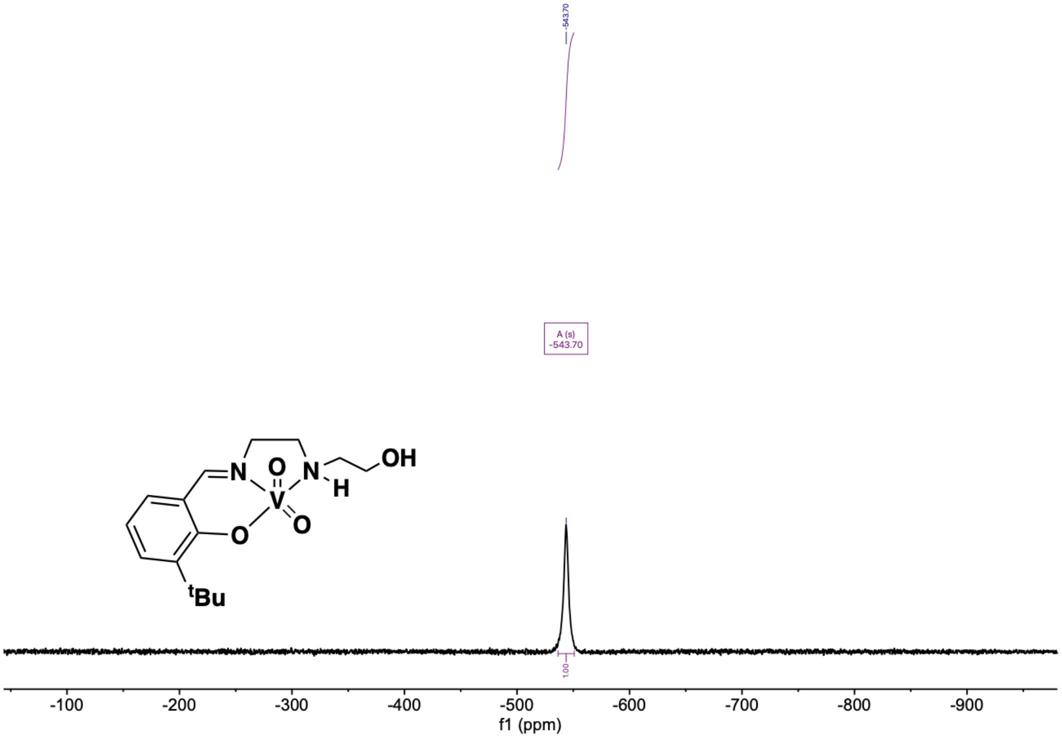

Figure S4 ^{51}V NMR of [VO_2(3-tBuHSHED)] in $CDCl_3$.

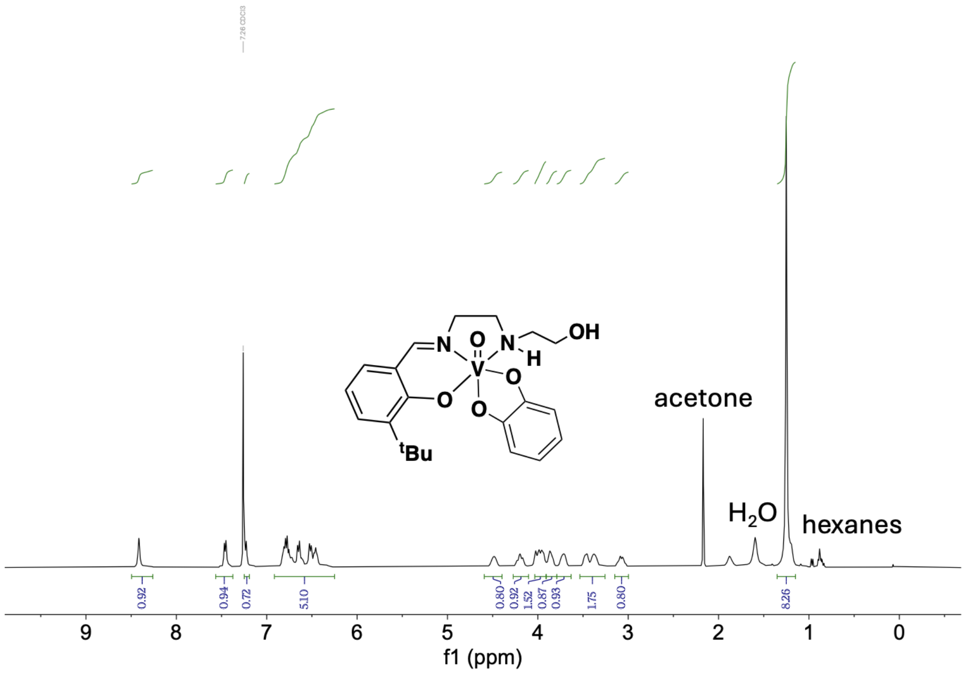

Figure S5 ^{1}H NMR of [VO(3-tBuHSHED)(CAT)] in $CDCl_3$.

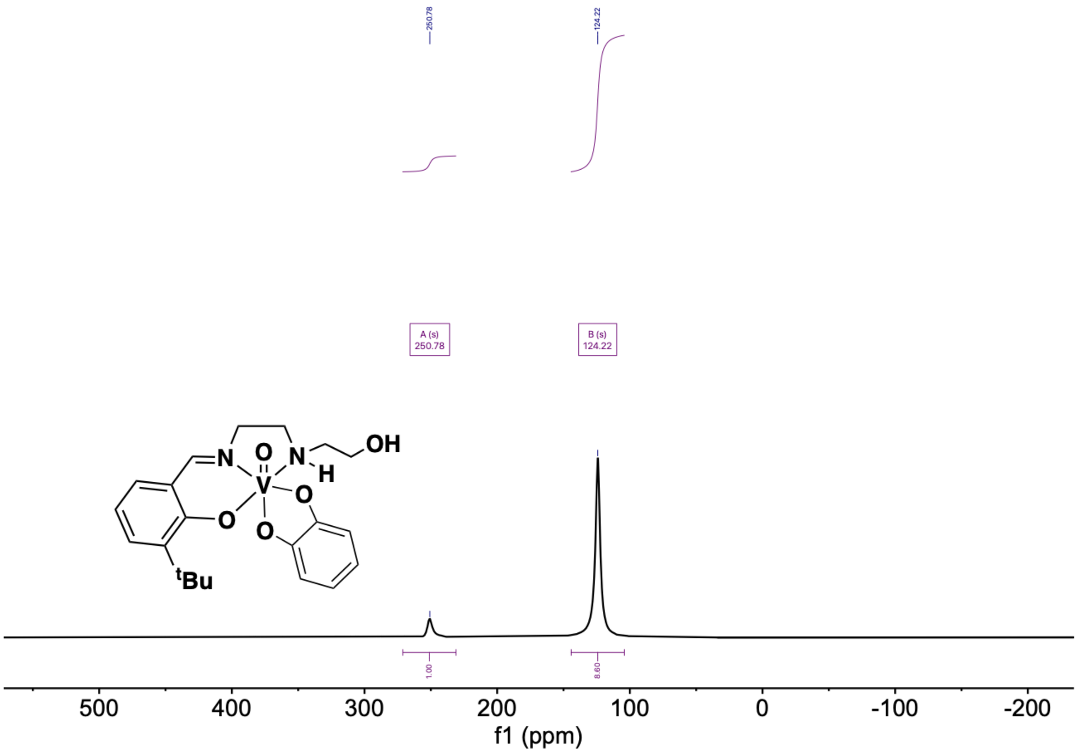

Figure S6 ^{51}V NMR of [VO(3-tBuHSHED)(CAT)] in $CDCl_3$.

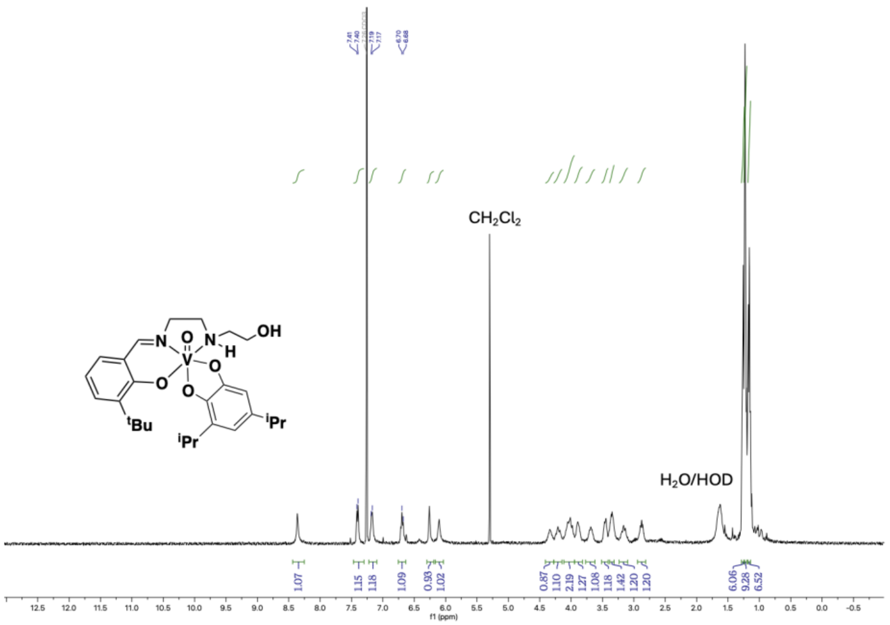

Figure S7 ^{1}H NMR of [VO(3-tBuHSHED)(DIPCAT)] in $CDCl_3$. Proton labeling scheme can be found in main text.

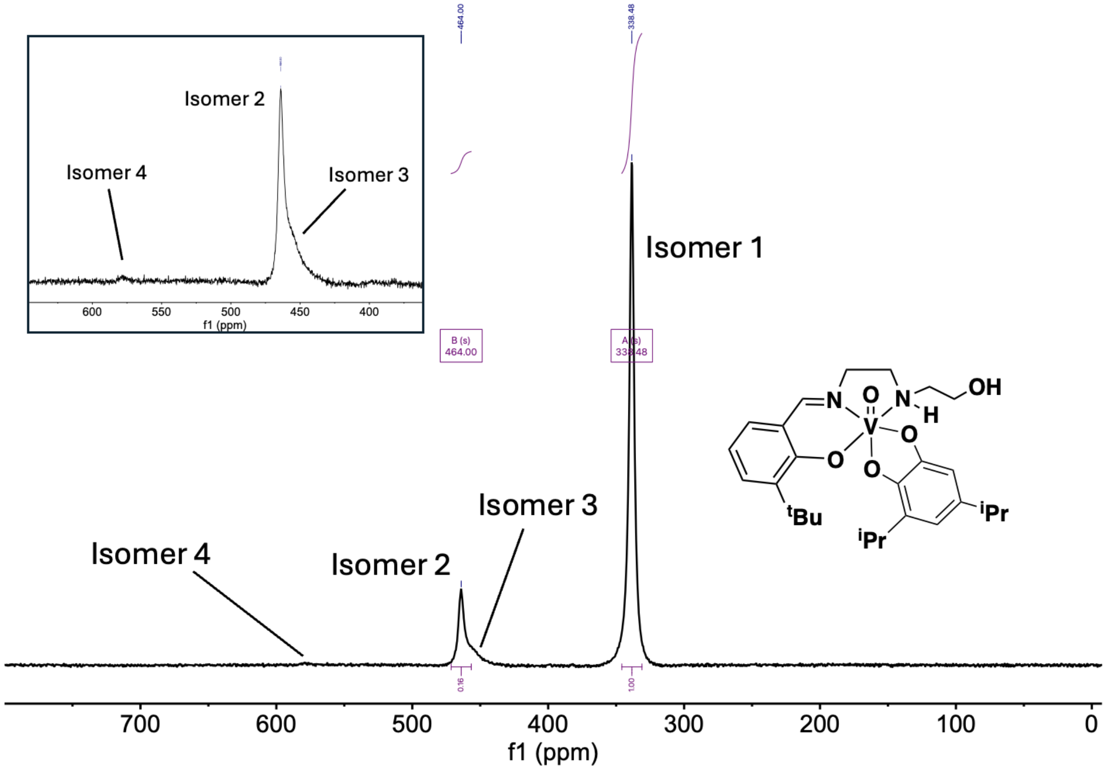

Figure S8 [51]V NMR of [VO(3-tBuHSHED)(DIPCAT)] in $CDCl_3$ with inset zoom of minor isomer peaks.

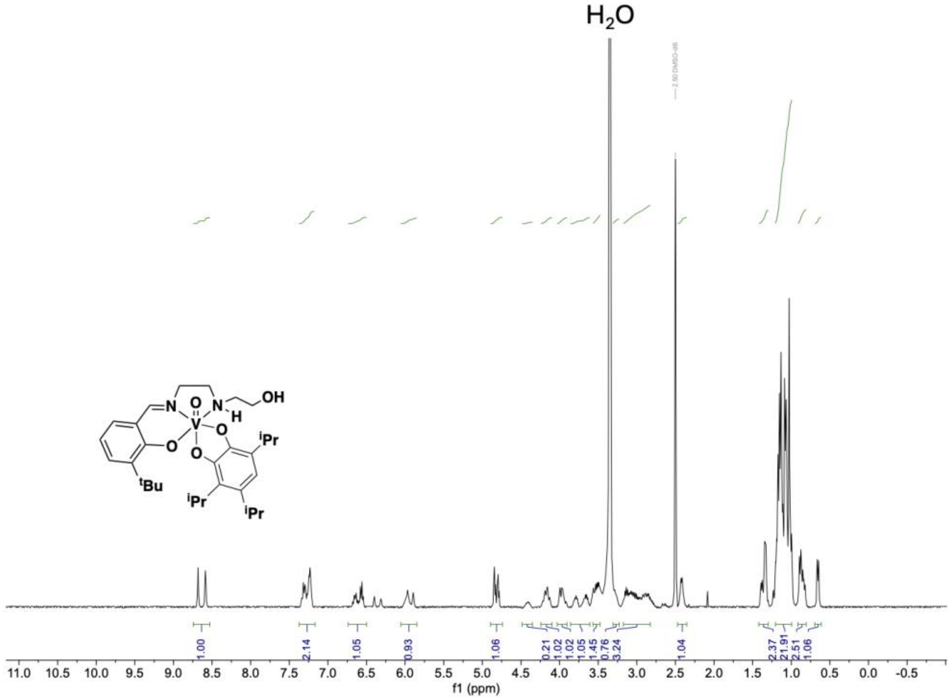

Figure S9 ^{1}H NMR of [VO(3-tBuHSHED)(TIPCAT)] in DMSO.

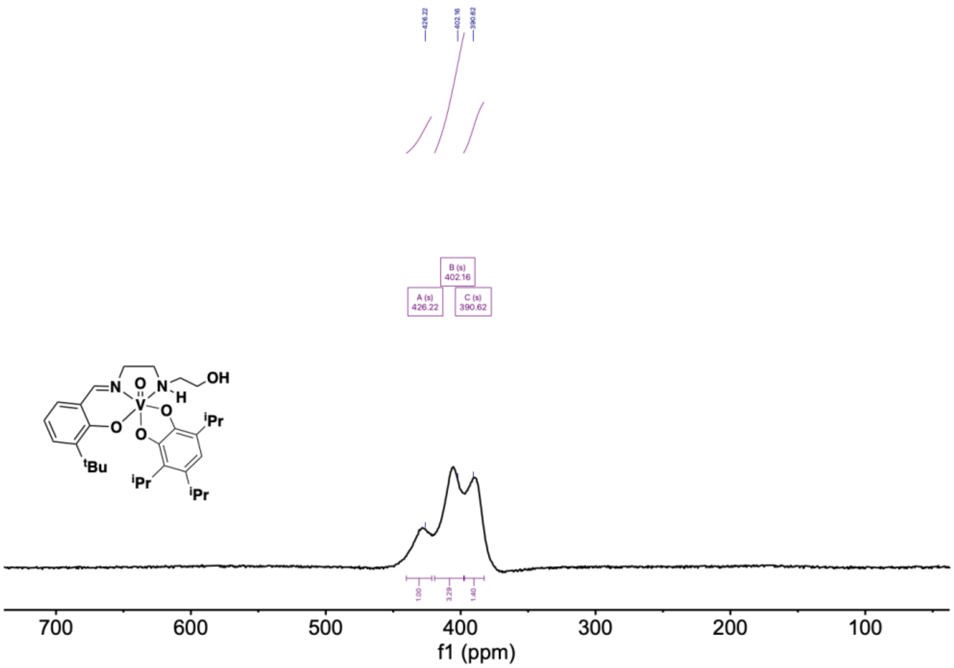

Figure S10 ^{51}V NMR of [VO(3-tBuHSHED)(TIPCAT)] in DMSO.

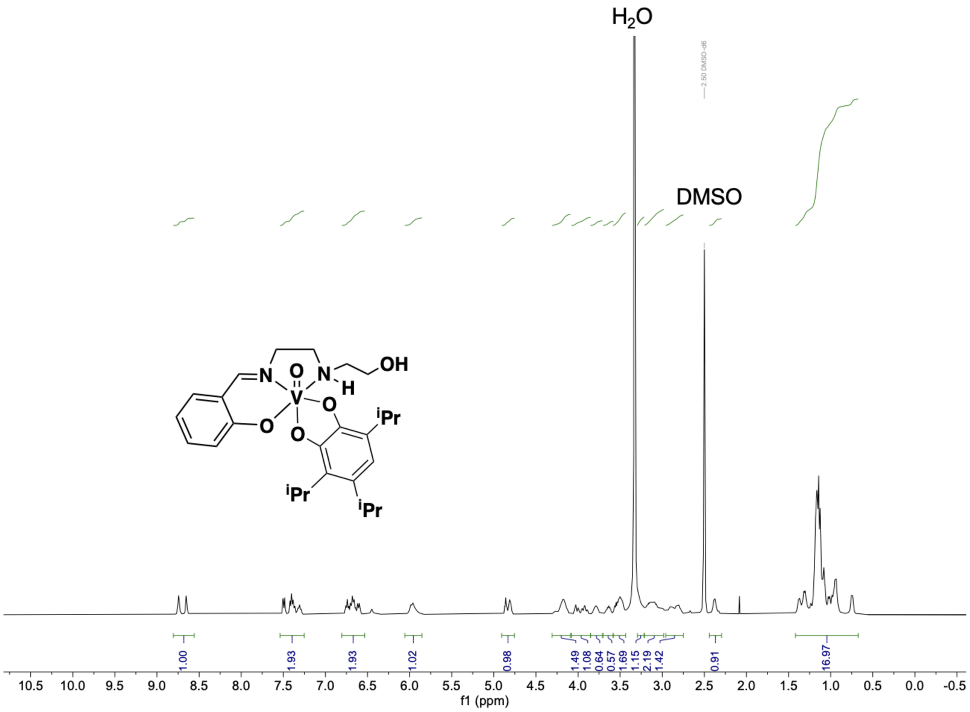

Figure S11 ^{1}H NMR of [VO(HSHED)(TIPCAT)] in DMSO.

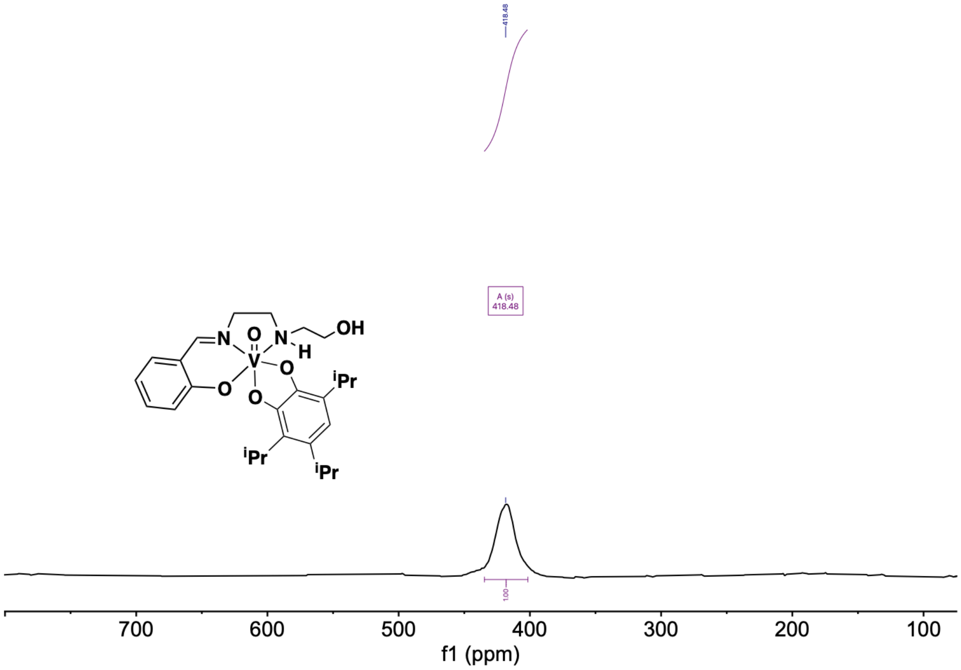

Figure S12 ^{51}V NMR of [VO(HSHED)(TIPCAT)] in DMSO

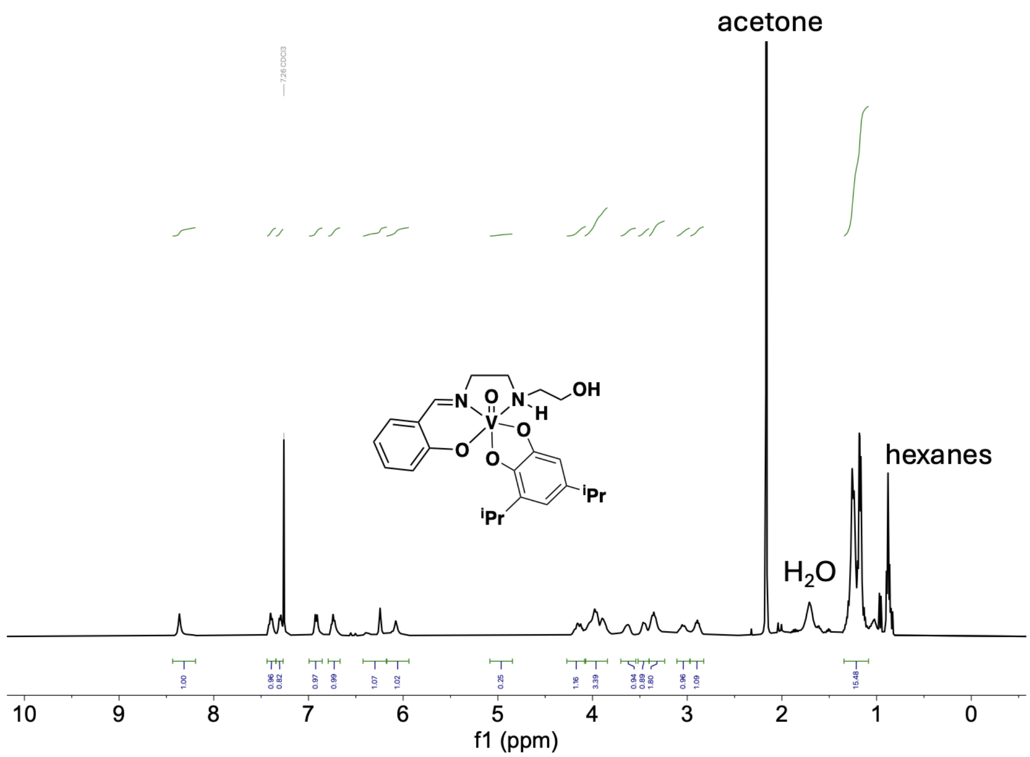

Figure S13 ^{1}H NMR of [VO(HSHED)(DIPCAT)] in $CDCl_3$.

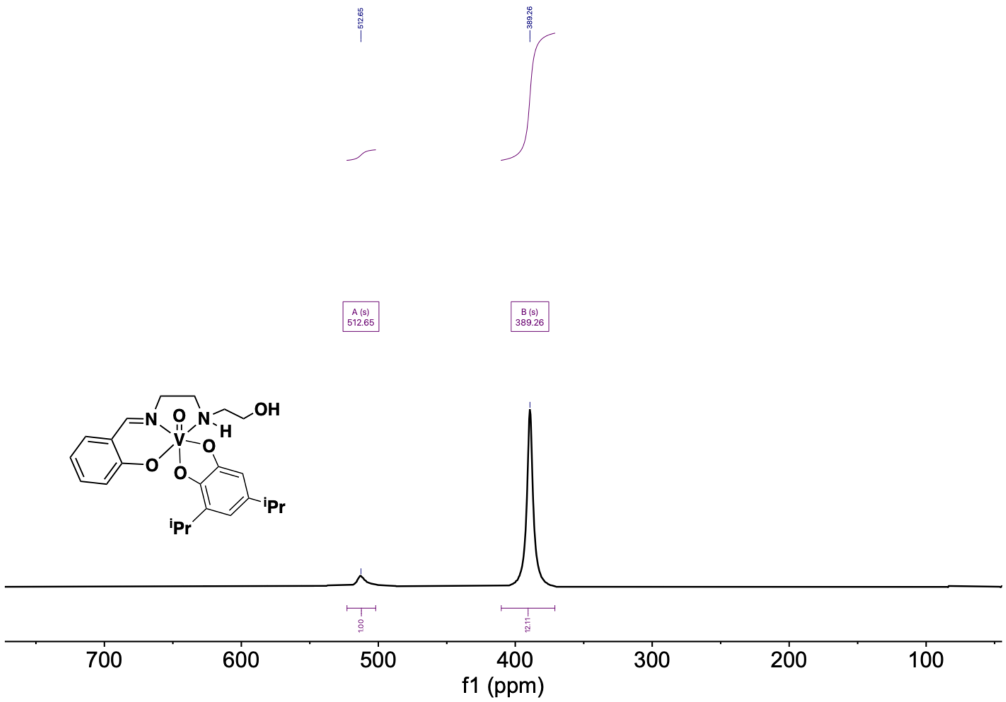

Figure S14 [51]V NMR of [VO(HSHED)(DIPCAT)] in $CDCl_3$.

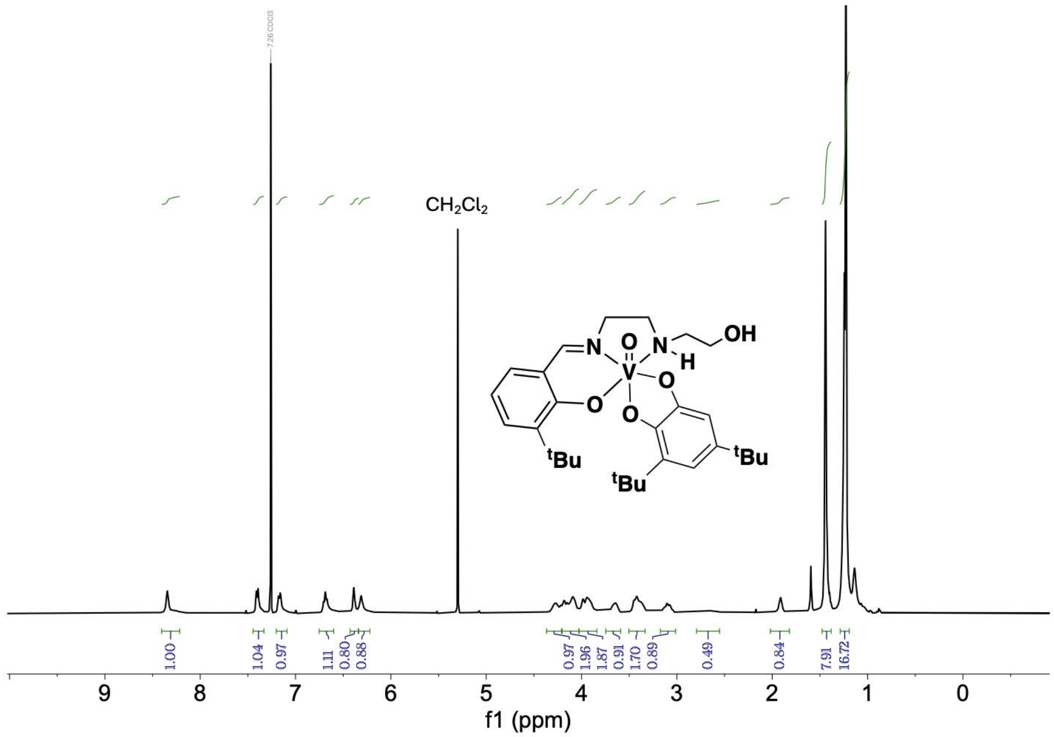

Figure S15 ^{1}H NMR of [VO(3-tBuHSHED)(DTB)] in $CDCl_3$.

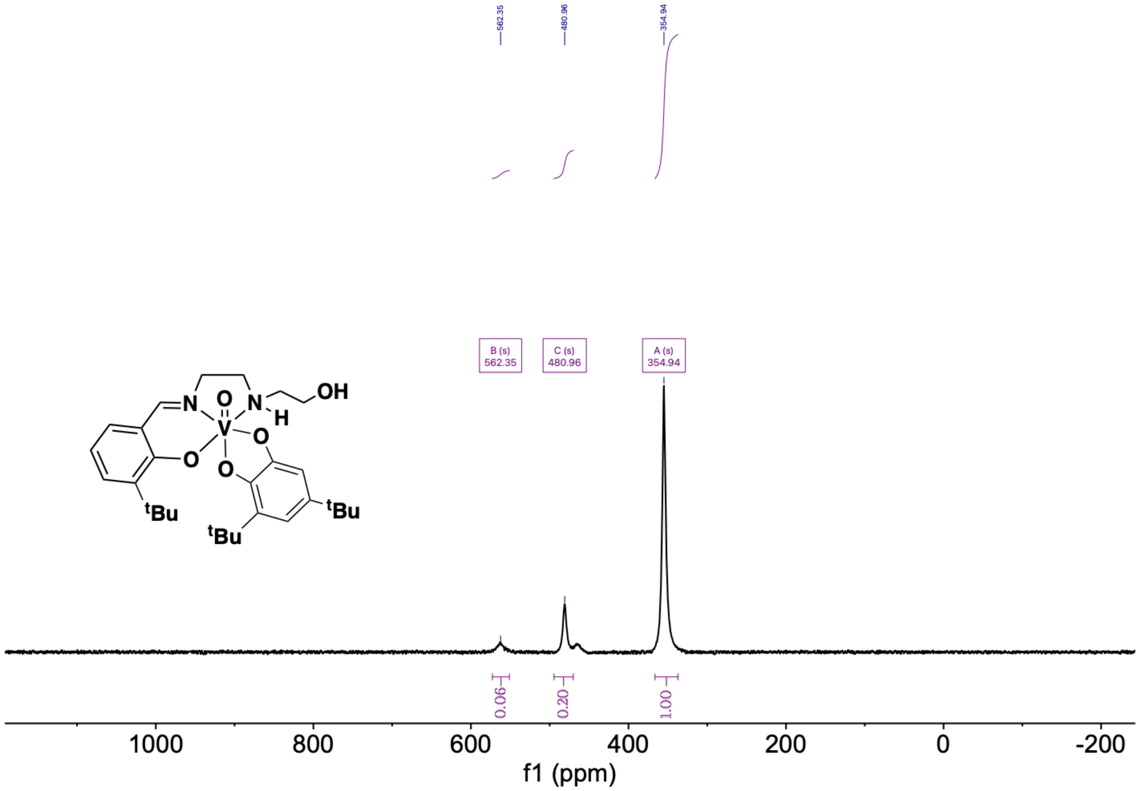

Figure S16 ^{51}V NMR of [VO(3-tBuHSHED)(DTB)] in $CDCl_3$

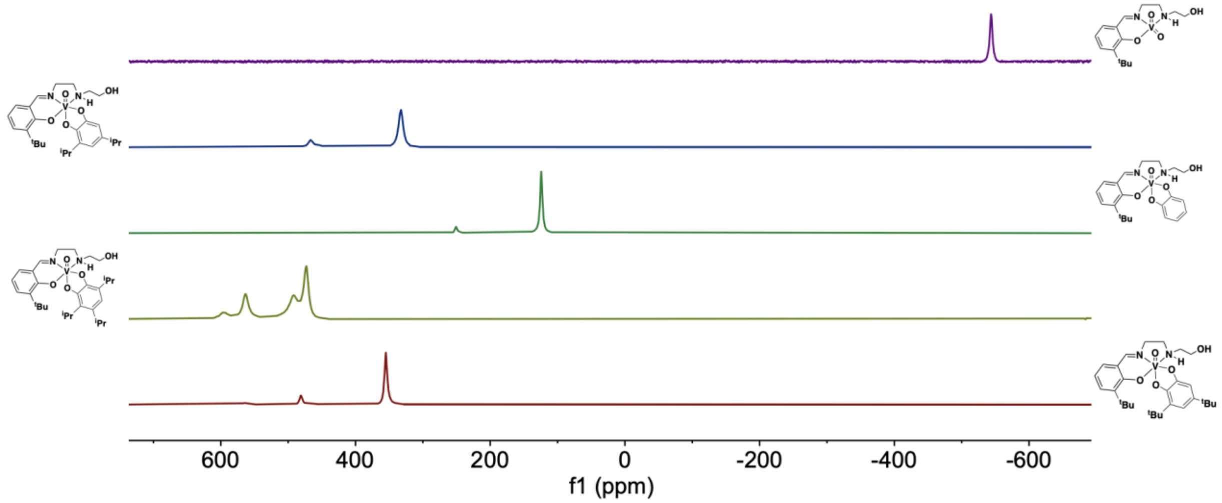

Figure S17 Stacked ^{51}V NMR plot of the [VO(3-tBuHSHED)] series in $CDCl_3$.

V. 2D NMR characterization

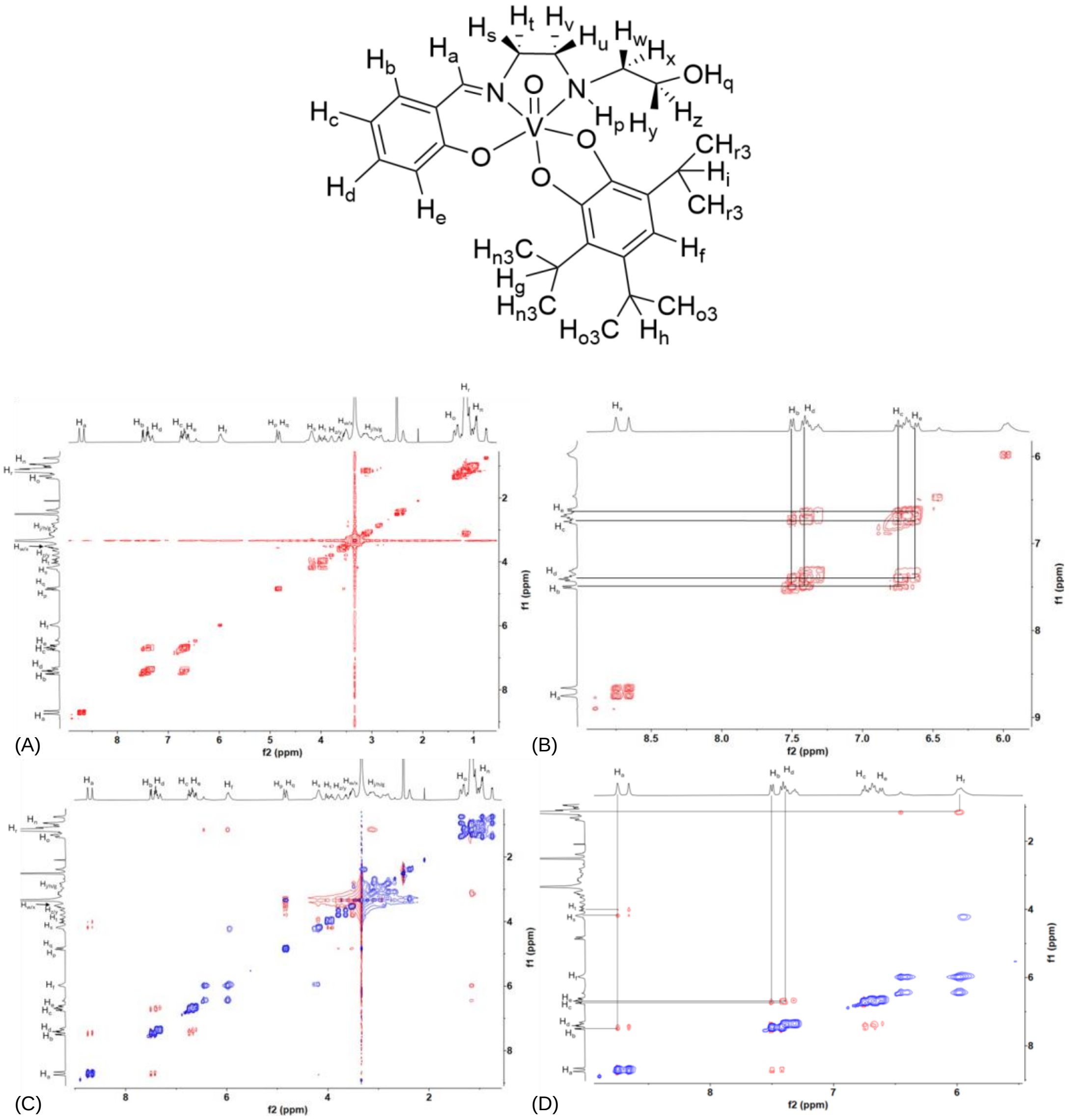

(A) (B) (C) (D)

Figure S18 Structure of [VO(HSHED)(TIPCAT)] is shown with a proton-labeling scheme. ^1H-^1H 2D COSY and ^1H-^1H 2D NOESY NMR (400 MHz) spectra were run at 10 mM in DMSO-d_6 at ambient temperature. (A) Full ^1H-^1H COSY spectrum of [VO(HSHED)(TIPCAT)]. (B) Zoom in of the aromatic region of ^1H-^1H COSY spectrum of [VO(HSHED)(TIPCAT)] showing crosstalk between aromatic protons on the Schiff base. (C) Full ^1H-^1H NOESY spectrum of [VO(HSHED)(TIPCAT)]. (D) Zoom in of aromatic region of ^1H-^1H NOESY spectrum showing cross peaks between imine proton (H_a) and ethylene protons ($H_{s/t}$). Red intensity contours

The chemical environment and energy of both isomer conformations for [VO(HSHED)(TIPCAT)] are very similar, therefore major isomer distinction is nontrivial and of small significance. The coordination of the catechol ligand to the vanadium does not lead to noticeable changes in the 2D analysis for this TIPCAT complex. Additionally, isomer distribution seen in ^{51}V NMR is confirmed

in the ^{1}H NMR spectra and the 2D work, since similar intensity peaks of each signal are present (confirming two major isomers of relatively equal ratios). This further complicates 2D work and proton assignments.

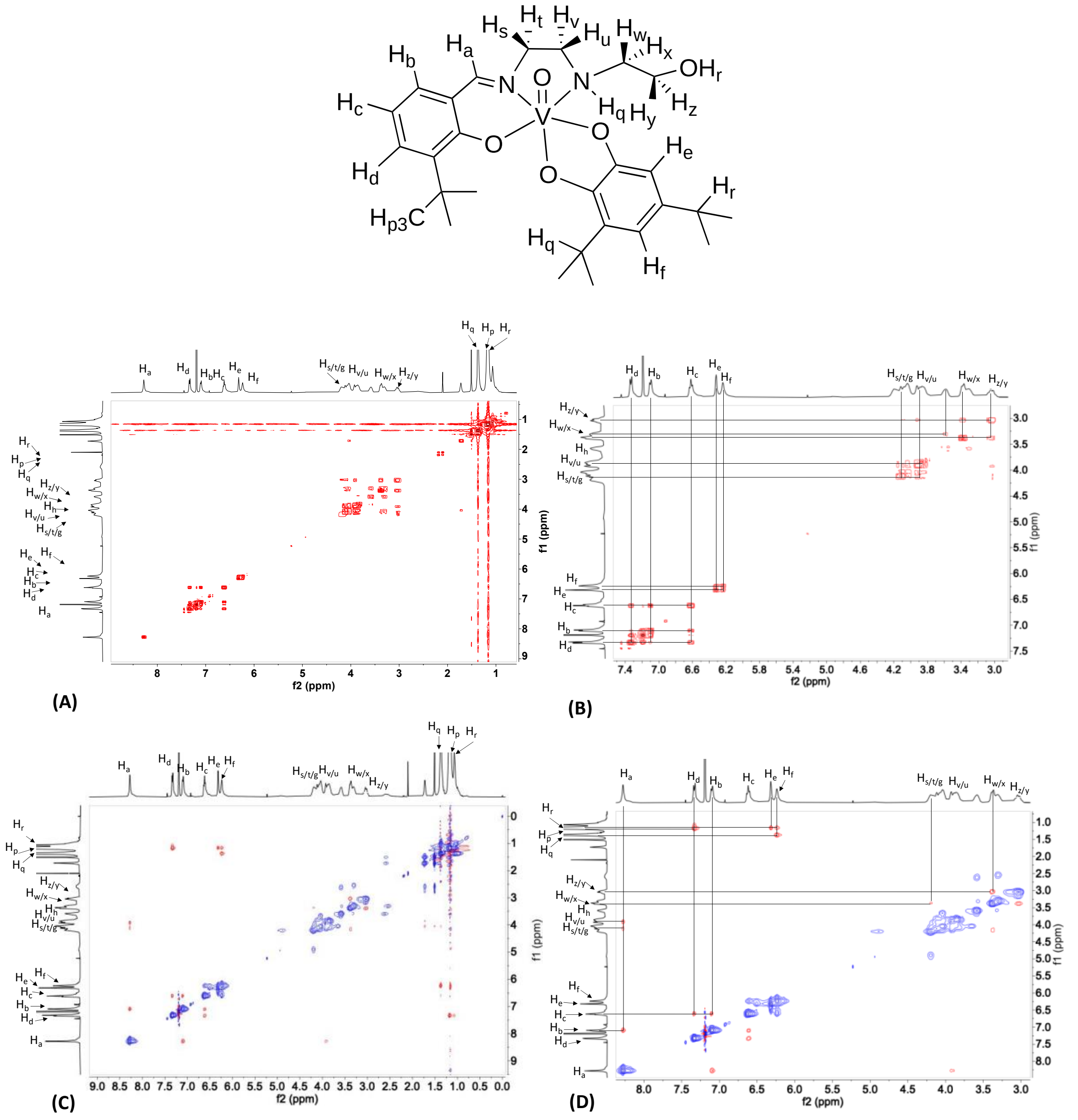

Figure S19 Structure of [VO(3-tBuHSHED)(DTB)] is shown with a proton-labeling scheme and major isomer structure. ^{1}H-^{1}H 2D COSY and ^{1}H-^{1}H 2D NOESY NMR (400 MHz) spectra were run at 10 mM in $CDCl_3$ at ambient temperature. (A) Full ^{1}H-^{1}H COSY spectrum (B) Zoom in of ^{1}H-^{1}H COSY spectrum of showing crosstalk between aromatic protons and select ethylene protons. (C) Full ^{1}H-^{1}H NOESY spectrum (D) Zoom in of ^{1}H-^{1}H NOESY spectrum showing cross peaks to determine *tert*-butyl assignments and select ethylene protons. Red intensity contours represent positive NOEs and blue intensity contours

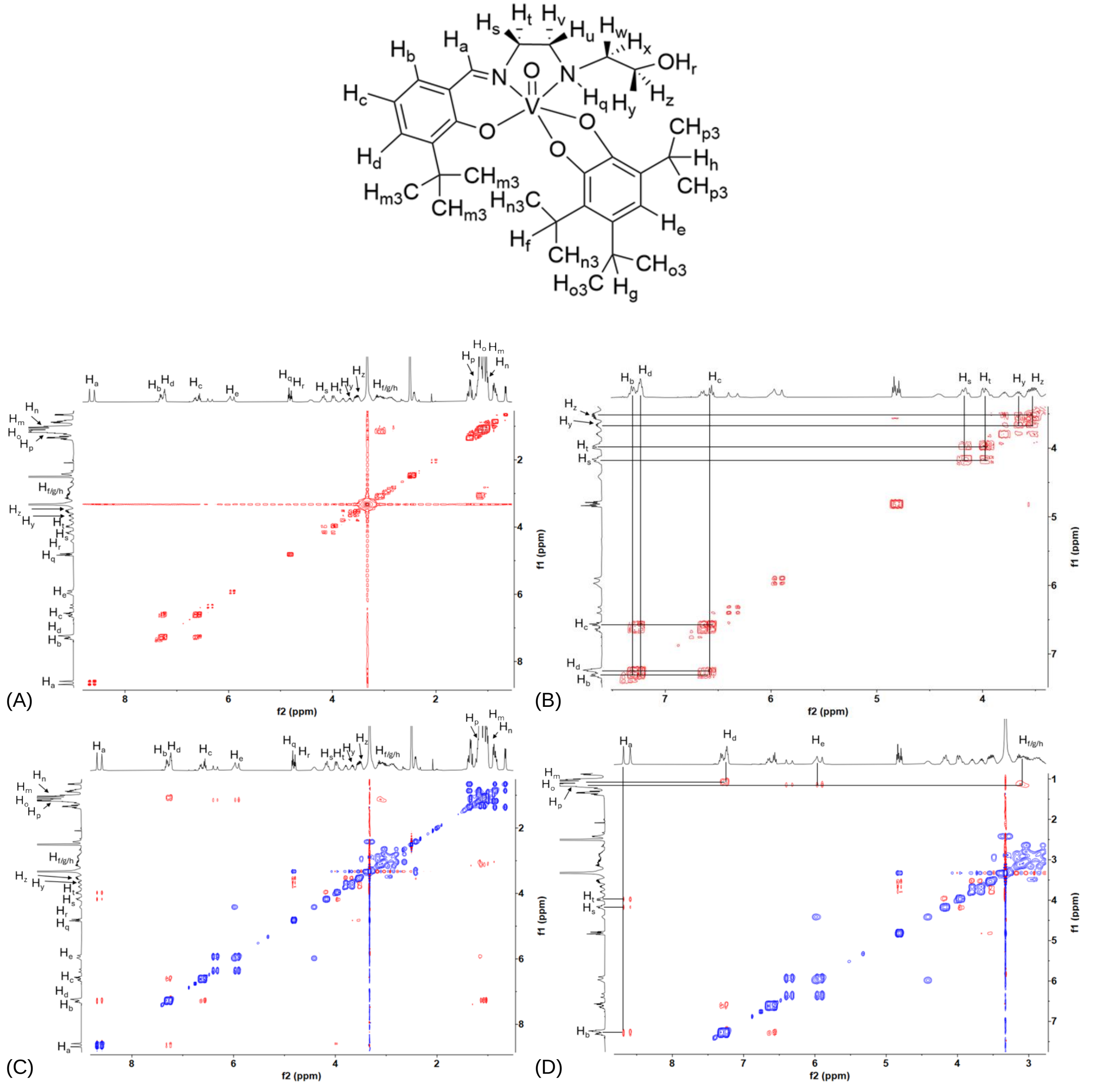

Figure S20 Structure of [VO(3-tBuHSHED)(TIPCAT)] is shown with a proton-labeling scheme. ^{1}H-^{1}H 2D COSY and ^{1}H-^{1}H 2D NOESY NMR (400MHz) spectra were run at 10 mM in DMSO-d_6 at ambient temperature. (A) Full ^{1}H-^{1}H COSY spectrum of [VO(3-tBuHSHED)(TIPCAT)]. (B) Zoom in of the aromatic and portion of the ethylene region ^{1}H-^{1}H COSY spectrum of [VO(3-tBuHSHED)(TIPCAT)] showing correlations between aromatic Schiff base protons and select ethylene protons. (C) Full ^{1}H-^{1}H NOESY spectrum of [VO(3-tBuHSHED)(TIPCAT)]. (D) Zoom in of ^{1}H-^{1}H NOESY spectrum showing cross peaks resolving aromatic systems. Red intensity contours represent positive NOEs and blue intensity contours

Overlapping peaks from 0.8 ppm – 1.5 ppm resulting from isomers and similar chemical environments of tert-butyl and *iso*-propyl protons complicate the NOE correlations. Clear correlations between the *iso*-propyl groups on the catechol ring and the tert-butyl group on Schiff base were not observed. Furthermore, no argument is presented identifying the major isomer for

this complex. Additionally, the chemical environment and energy of both isomer orientations are very similar, making major isomer distinction insignificant.

VI. FTIR Characterization

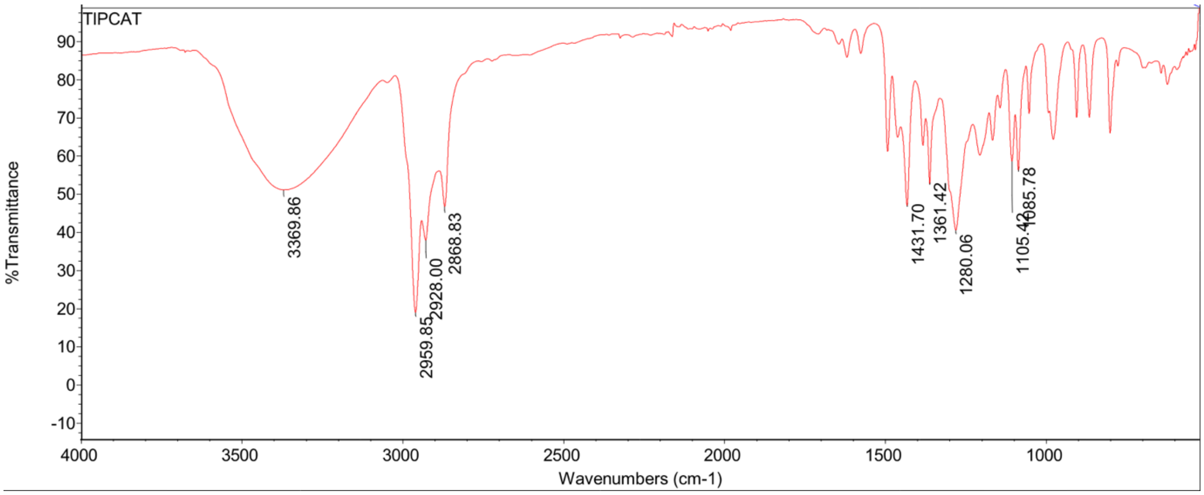

Figure S21 FTIR of 3,4,6-tri-*iso*-propyl catechol

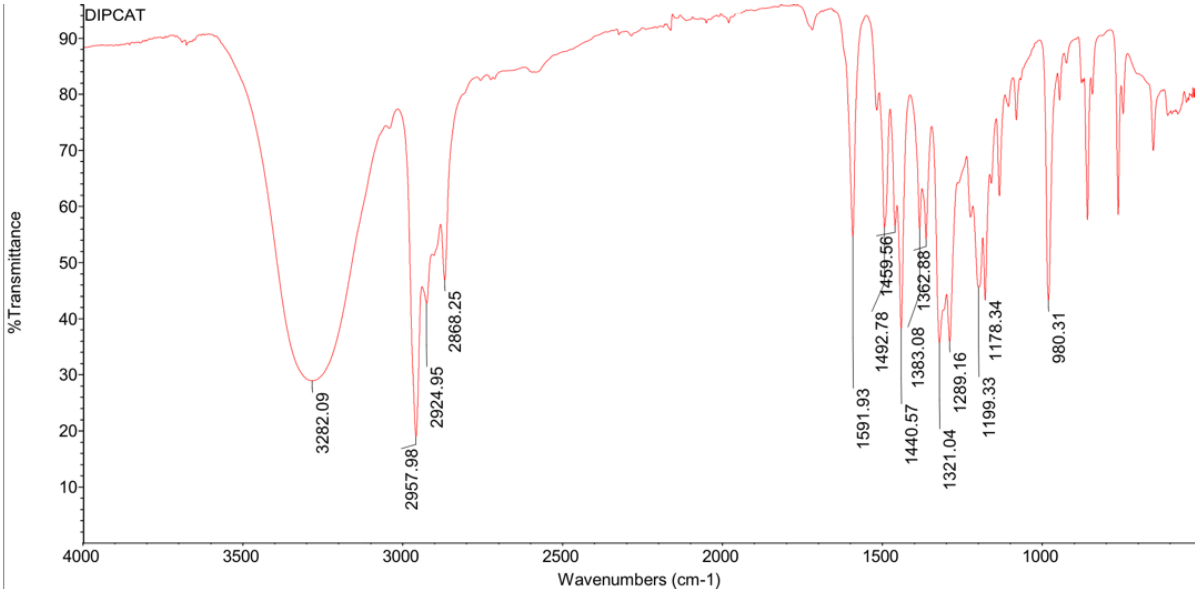

Figure S22 FTIR of 3,5-di-*iso*-propyl catechol

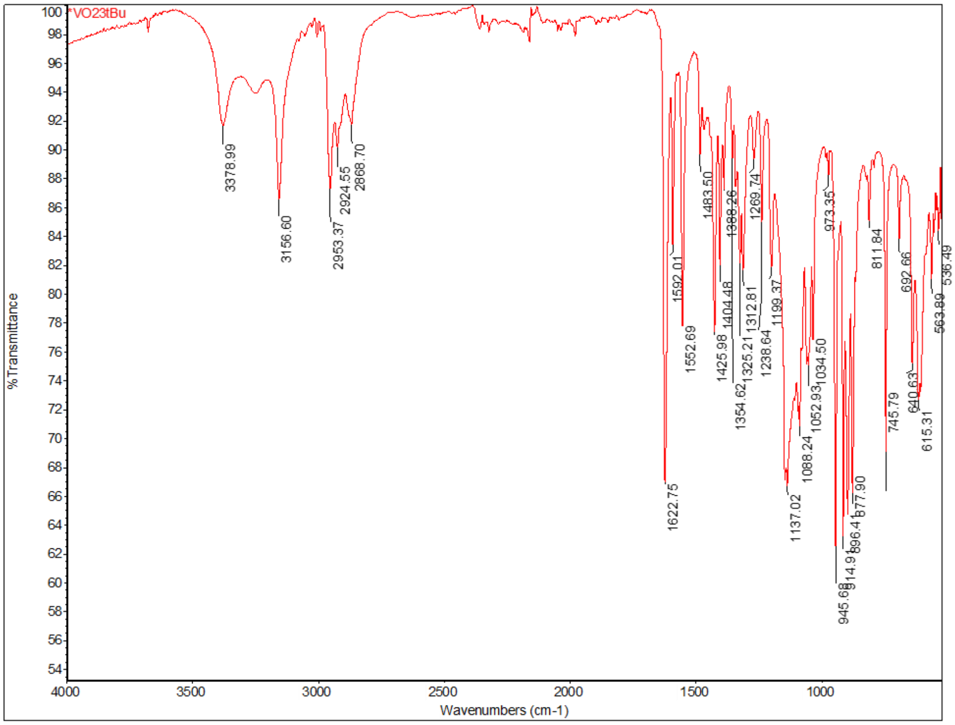

Figure S23 FTIR of [VO_2(3-tBuHSHED)]

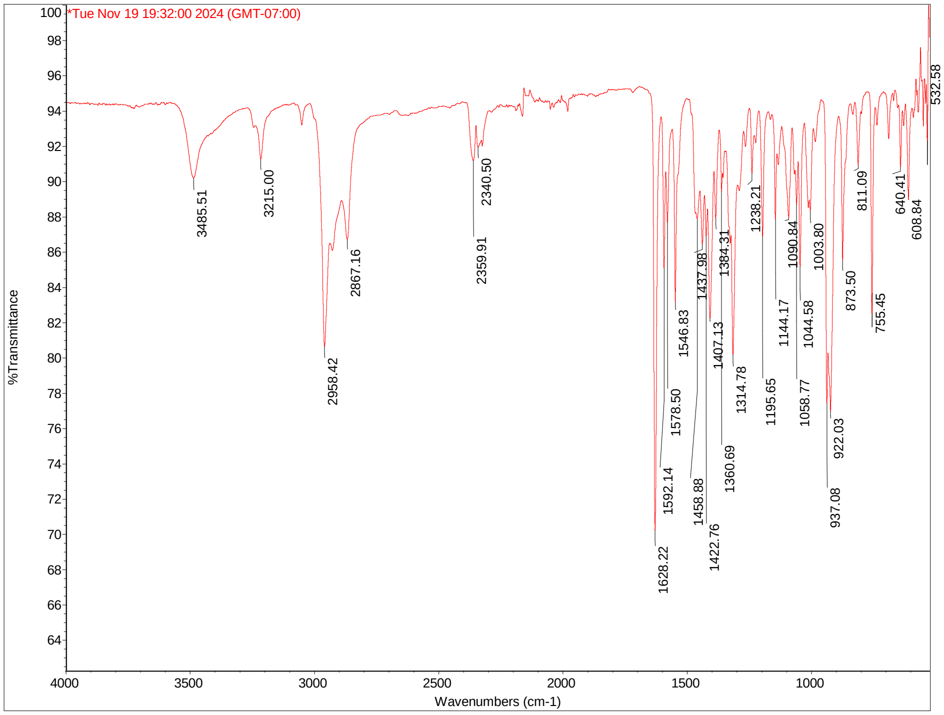

Figure S24 FTIR of [VO(3-tBuHSHED)(TIPCAT)]

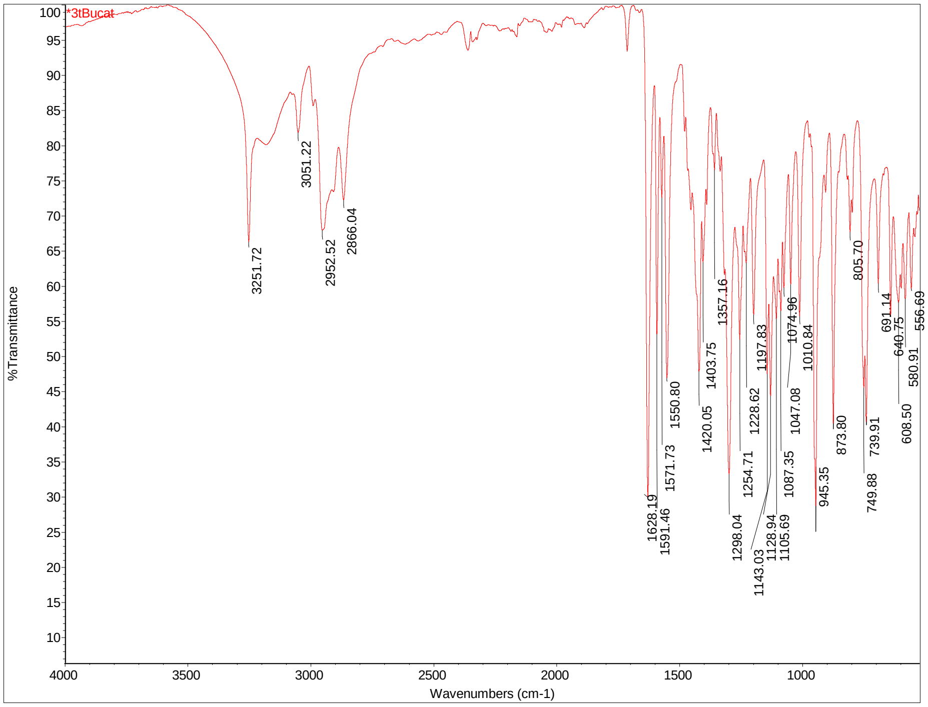

Figure S25 FTIR of [VO(3-tBuHSHED)(CAT)]

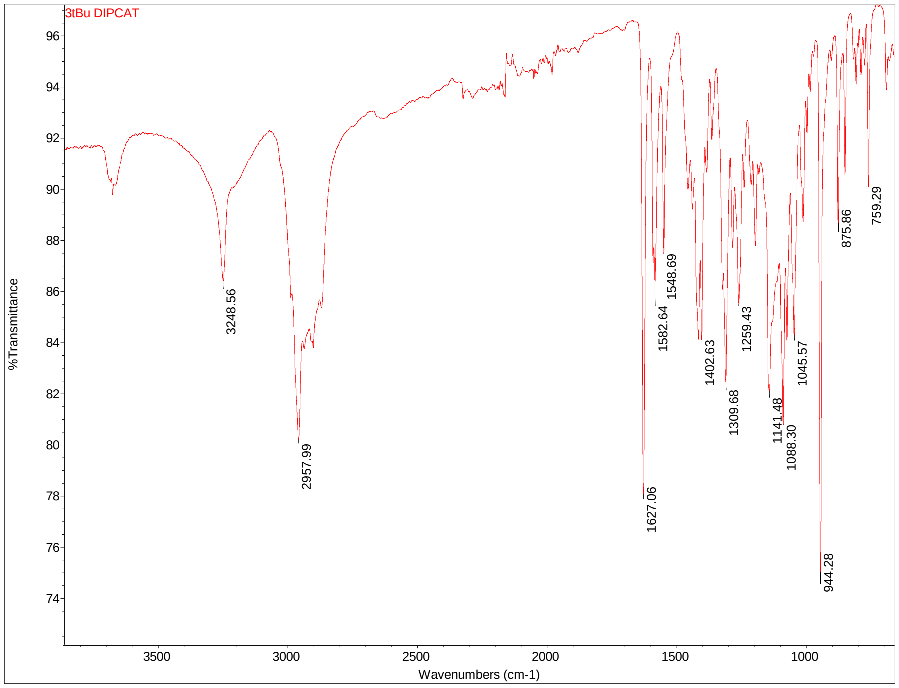

Figure S26 FTIR of [VO(3-tBuHSHED)(DIPCAT)]

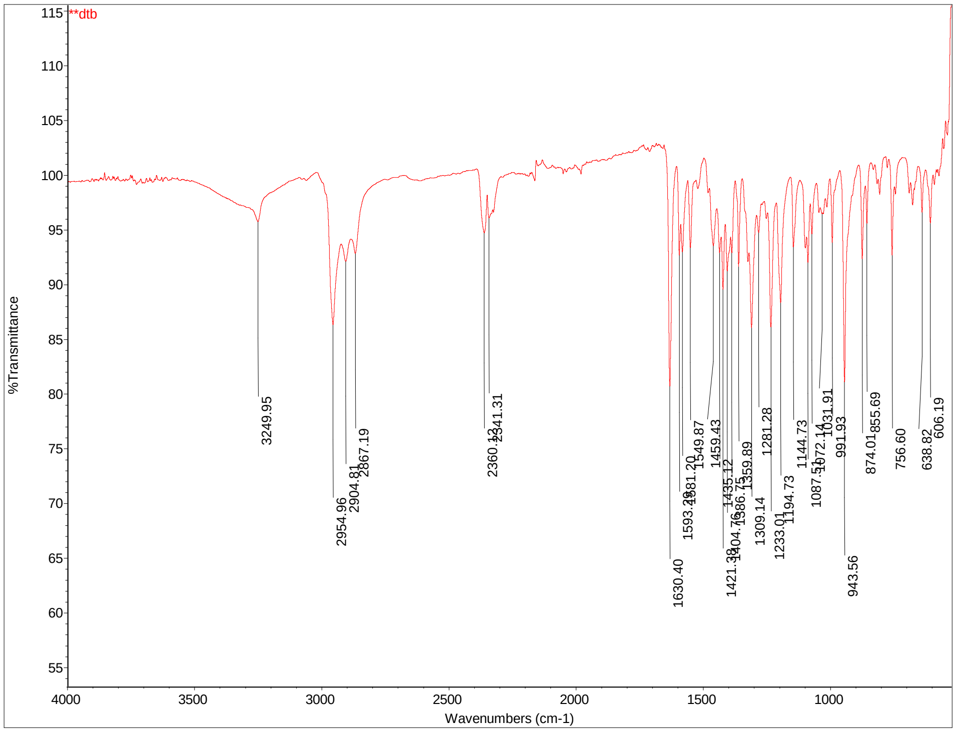

Figure S27 FTIR of [VO(3-tBuHSHED)(DTB)].

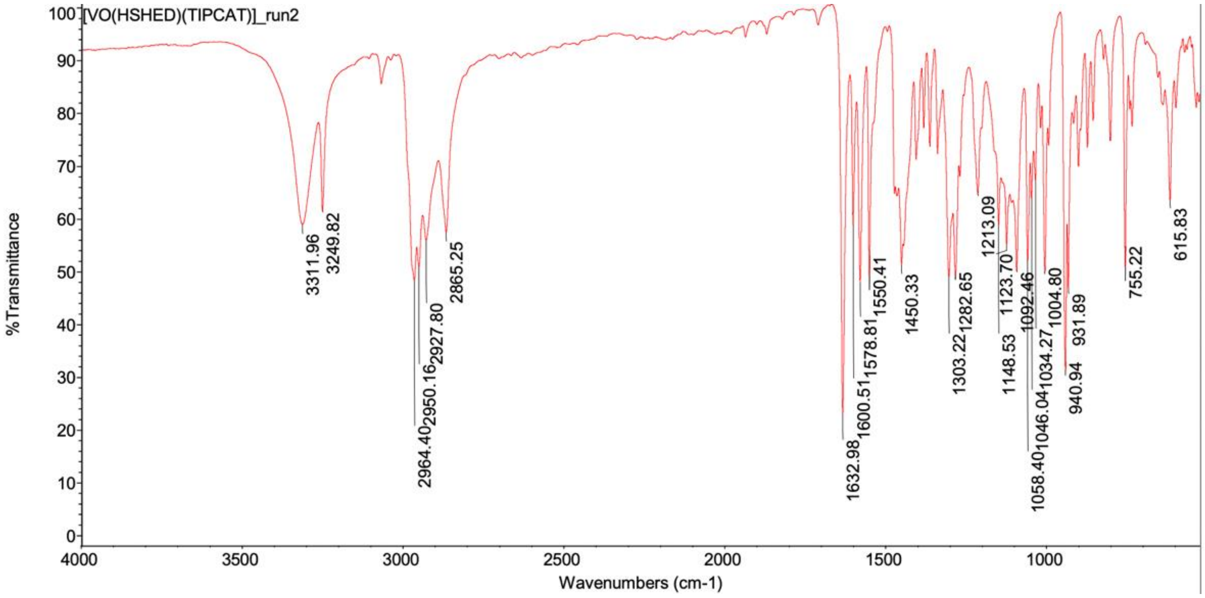

Figure S28 FTIR of [VO(HSHED)(TIPCAT)].

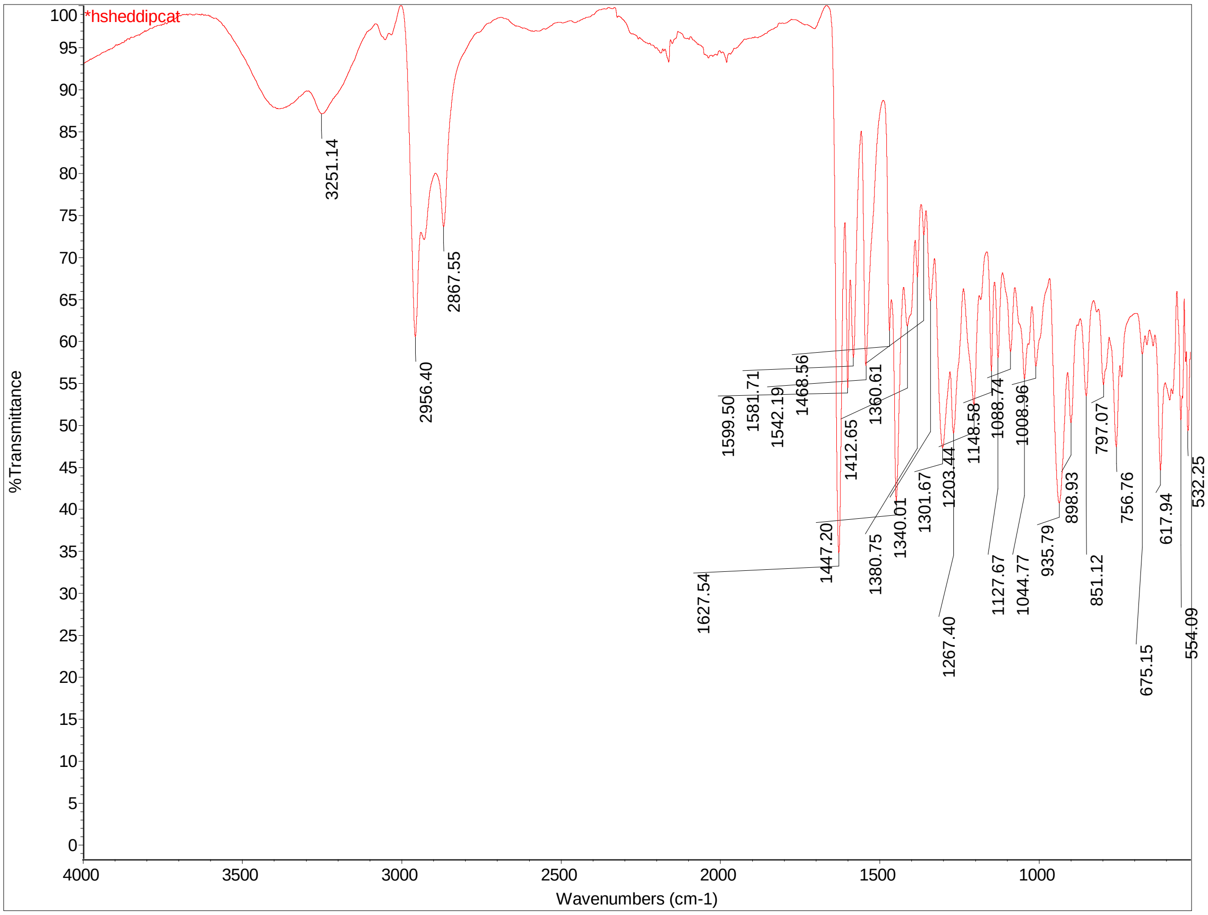

Figure S29 FTIR of [VO(HSHED)(DIPCAT)]

VII. Mass Spectrometry

Generic Display Report

Analysis Info

Analysis Name	D:\OneDrive - The University of Sydney (Staff)\Instrument_Data\BrukerSolarix2XR\Data\MS Facility\Chianna Dane\20240723\20240723_Service_HiRes_ESI_000014.d	Acquisition Date	7/23/2024 5:05:47 PM
Method	20240723 ESI Pos Neg 150-3000 4M-updatedshim AMP	Operator	Admin
Sample Name	V99	Instrument	solariX 2xR
Comment	MeCN		

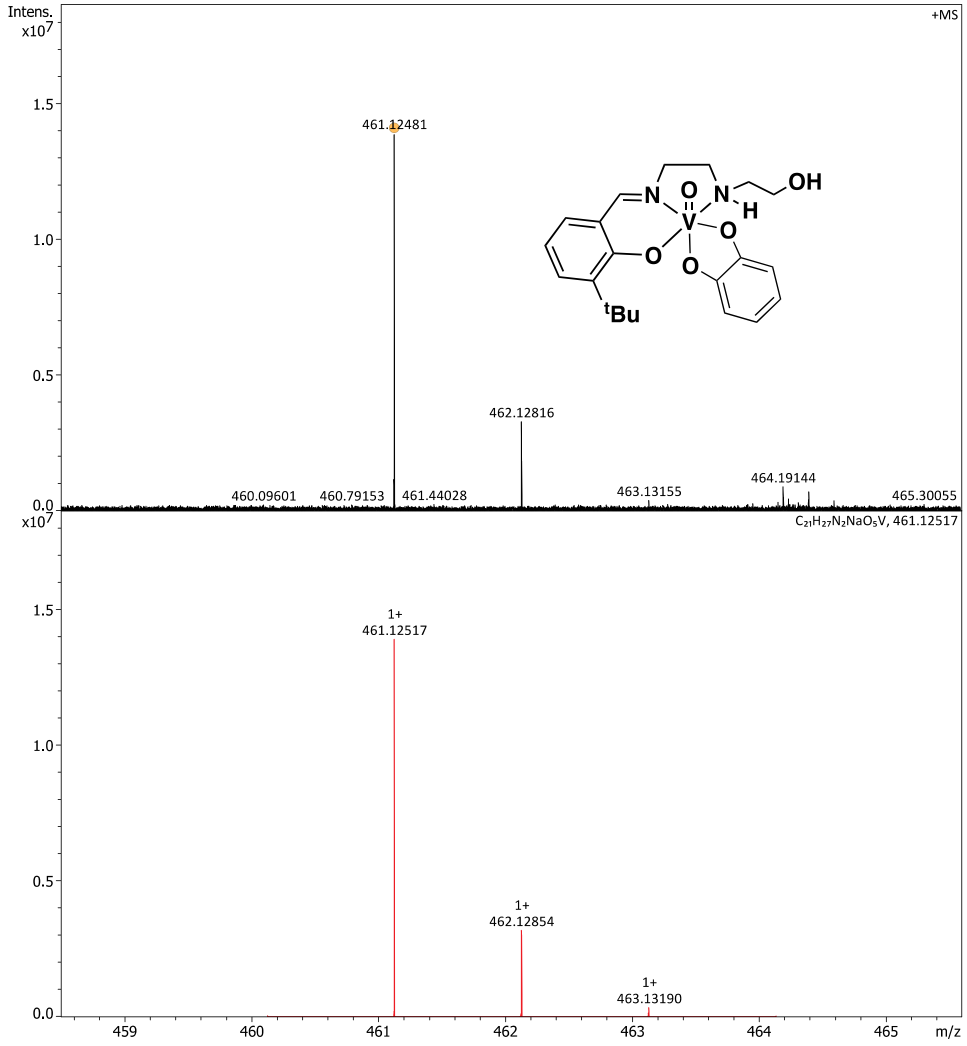

Bruker Compass DataAnalysis 6.0 printed: 23-Jul-24 5:09:26 PM by: demo Page 1 of 1

Figure S30 Experimental (top) and simulated (bottom) HRMS spectra of [VO(3-tBuHSHED)(CAT)]

Generic Display Report

Analysis Info

Analysis Name	D:\OneDrive - The University of Sydney (Staff)\Instrument_Data\BrukerSolarix2XR\Data\MS Facility\Chianna Dane\20240723\20240723_Service_HiRes_ESI_000012.d	Acquisition Date	7/23/2024 4:59:16 PM
Method	20240723 ESI Pos Neg 150-3000 4M-updatedshim AMP	Operator	Admin
Sample Name	V97	Instrument	solariX 2xR
Comment	MeCN		

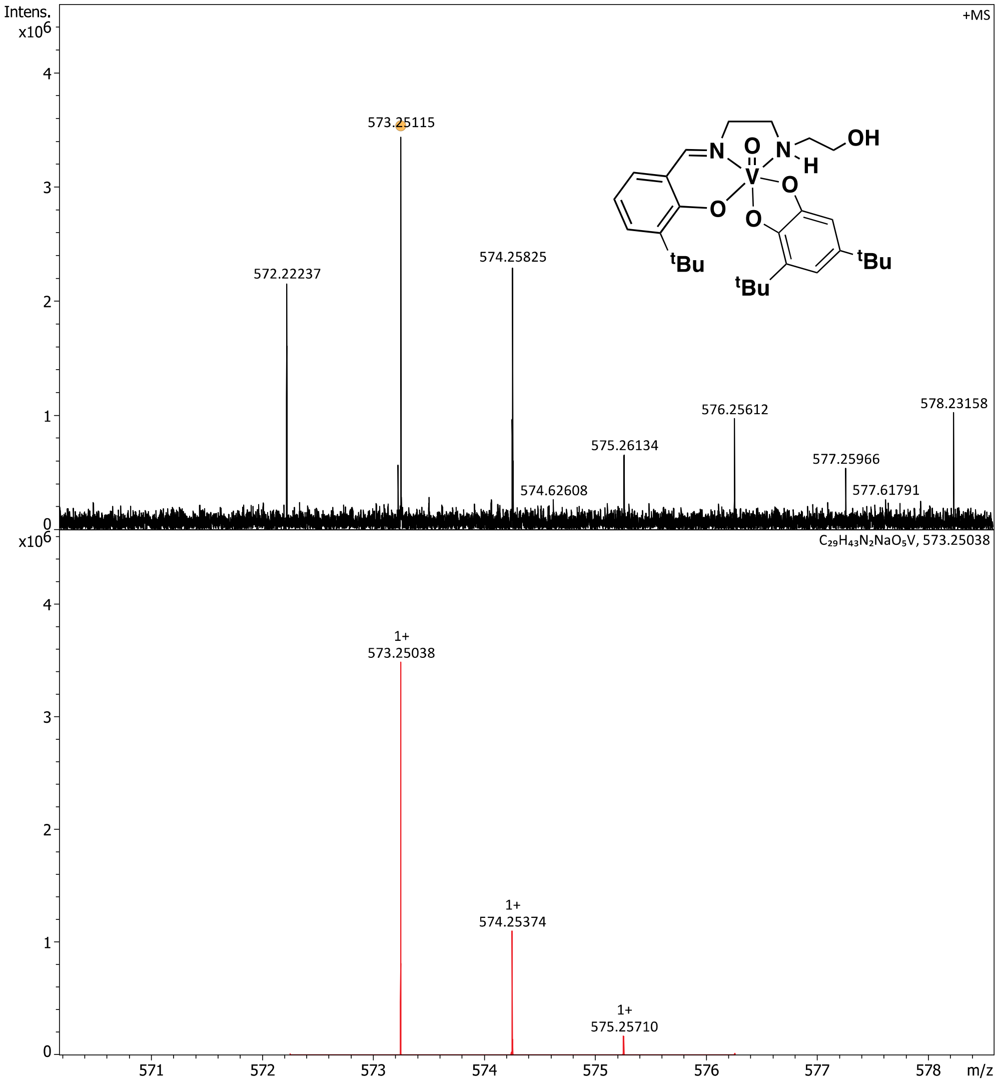

Bruker Compass DataAnalysis 6.0 printed: 23-Jul-24 5:01:24 PM by: demo Page 1 of 1

Figure S31 Experimental (top) and simulated (bottom) HRMS spectra of [VO(3-tBuHSHED)(DTB)]

Generic Display Report

Analysis Info

		Acquisition Date	7/23/2024 5:30:45 PM
Analysis Name	D:\OneDrive - The University of Sydney (Staff)\Instrument_Data\BrukerSolarix2XR\Data\MS Facility\Chianna Dane\20240723\20240723_Service_HiRes_ESI_000019.d		
Method	20240723 ESI Pos Neg 150-3000 4M-updatedshim AMP	Operator	Admin
Sample Name	V175	Instrument	solariX 2xR
Comment	MeCN		

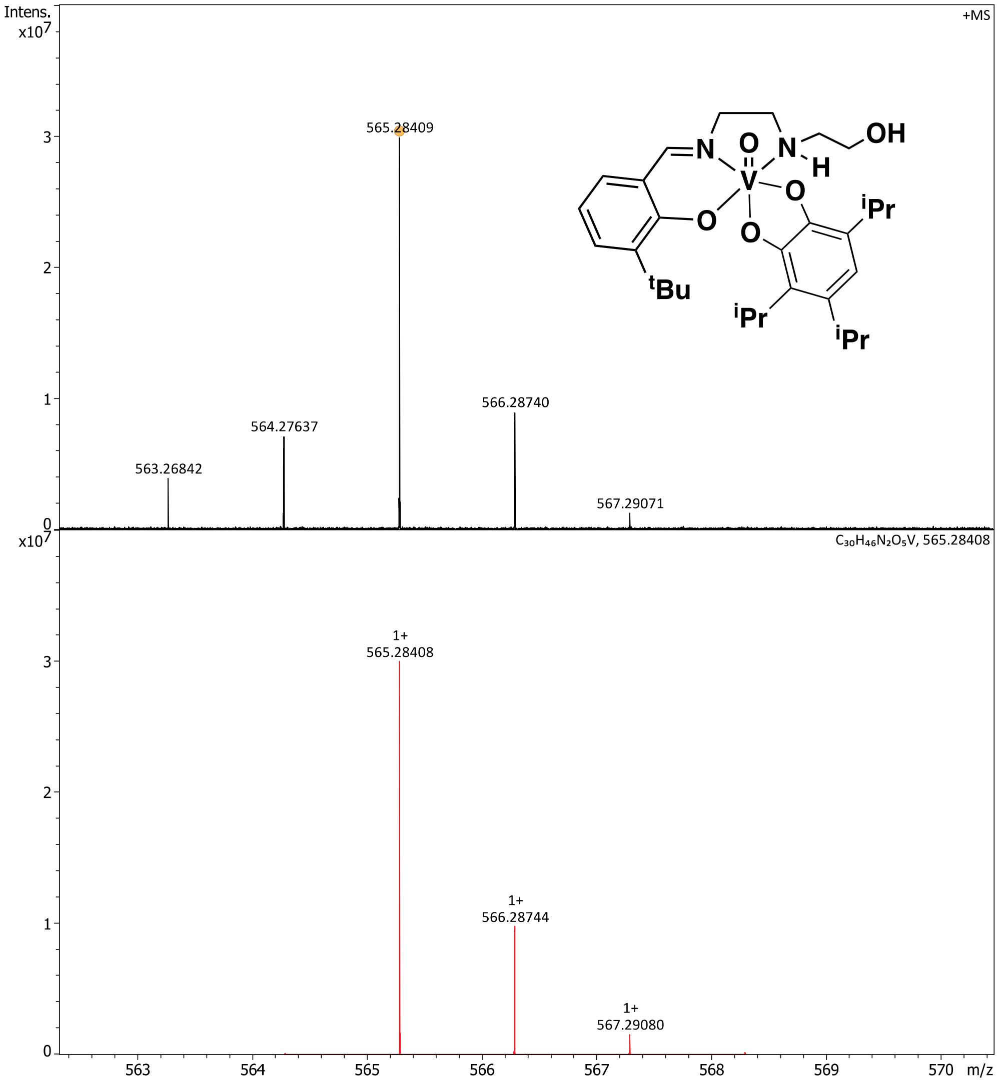

Figure S32 Experimental (top) and simulated (bottom) HRMS spectra of [VO(3-tBuHSHED)(TIPCAT)]

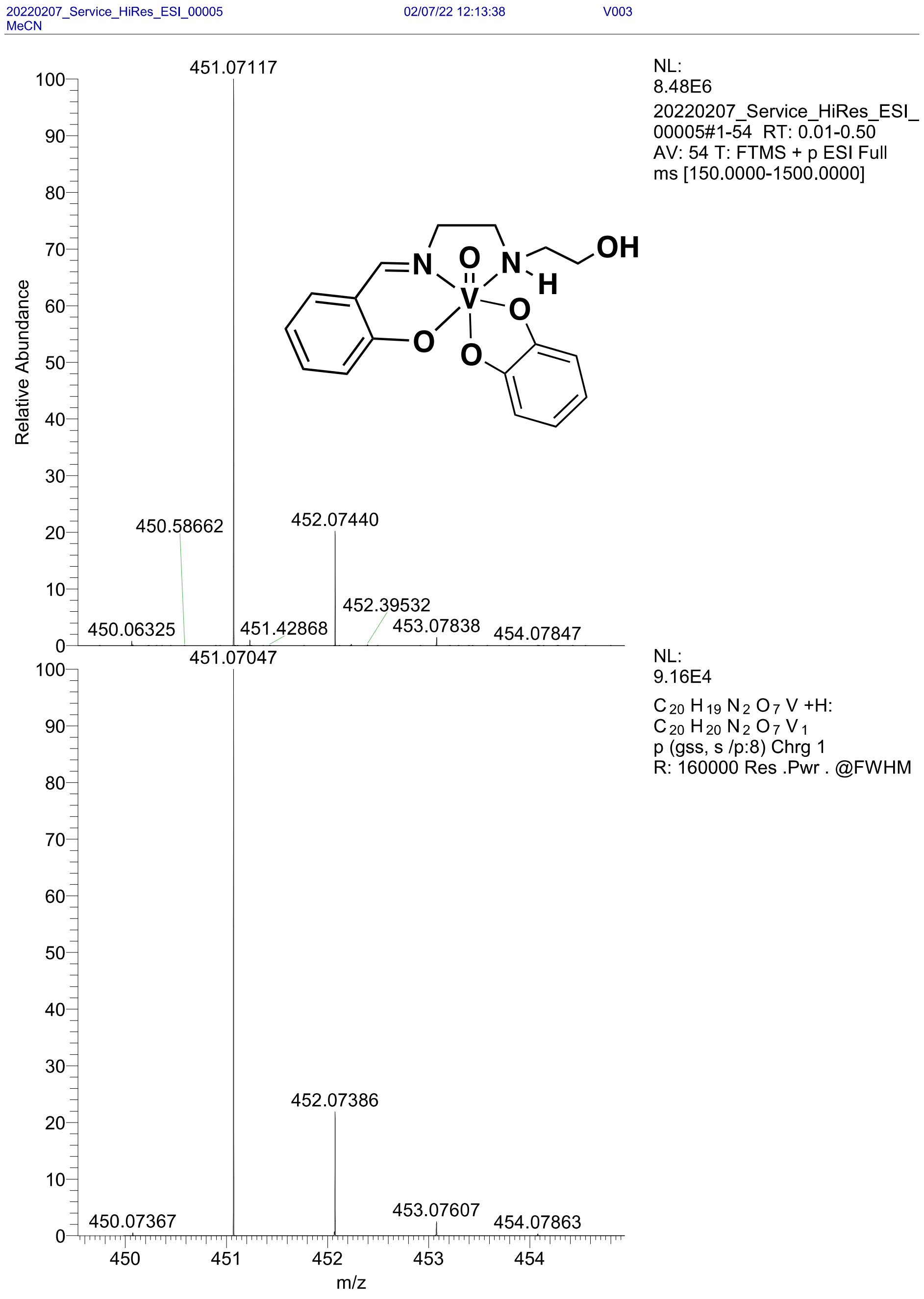

Figure S33 Experimental (top) and simulated (bottom) HRMS spectra of [VO(HSHED)(CAT)]

Display Report

Analysis Info

Analysis Name	D:\OneDrive - The University of Sydney (Staff)\Instrument_Data\BrukerSolarix2XR\Data\MS Facility\Chianna Dane\20230802\20230802_Service_HiRes_ESI_000004.d	Acquisition Date	8/2/2023 5:01:43 PM
Method	20230802 ESI Pos Neg 150-3000 4M-updatedshim	Operator	Admin
Sample Name	V1	Instrument	solariX 2xR
Comment	MeCN		

Acquisition Parameter

Acquisition Mode	Single MS	Acquired Scans	16	Calibration Date	Wed Aug 2 04:58:52 2023
		No. of Cell Fills	1	Data Acquisition Size	4194304
Broadband Low Mass	150.5 m/z			Data Processing Size (SI)	8388608
Broadband High Mass	3000.0 m/z	Laser Power	100.0 lp	Apodization	Full-Sine
Source Accumulation	0.000 sec				
Ion Accumulation Time	0.010 sec				

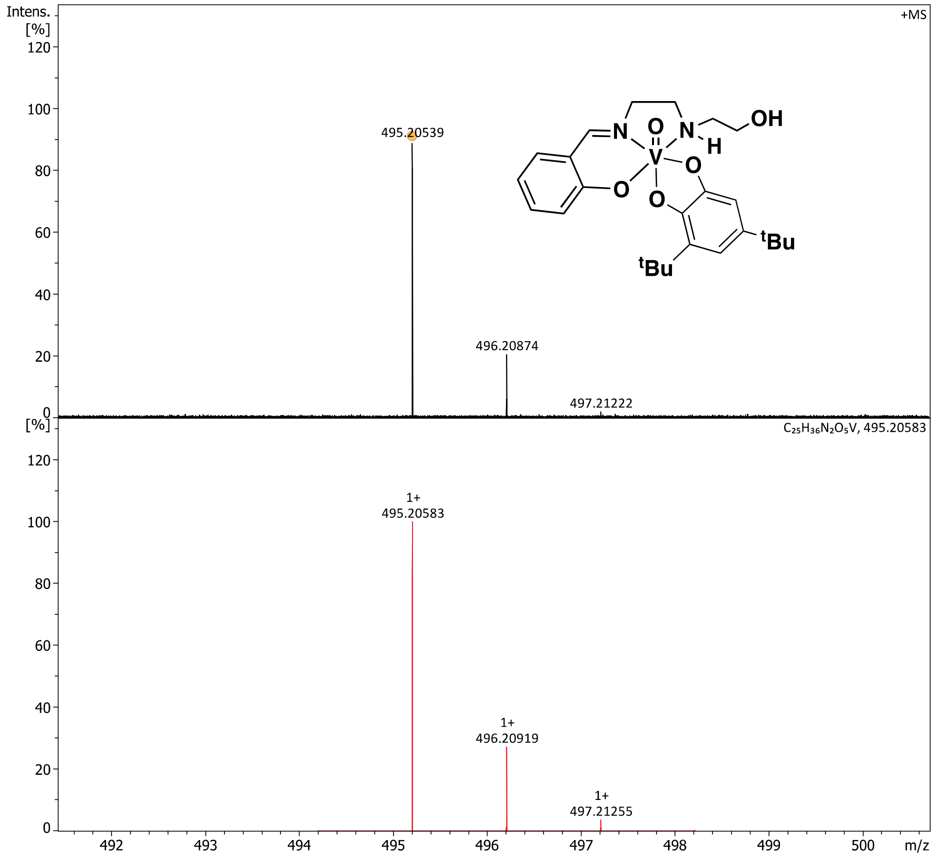

Figure S34 Experimental (top) and simulated (bottom) HRMS spectra of [VO(HSHED)(DTB)]

Generic Display Report

Analysis Info

Analysis Name	D:\OneDrive - The University of Sydney (Staff)\Instrument_Data\BrukerSolarix2XR\Data\MS Facility\Chianna Dane\20240723\20240723_Service_HiRes_ESI_000020.d	Acquisition Date	7/23/2024 5:34:13 PM
Method	20240723 ESI Pos Neg 150-3000 4M-updatedshim AMP	Operator	Admin
Sample Name	V195	Instrument	solariX 2xR
Comment	MeCN		

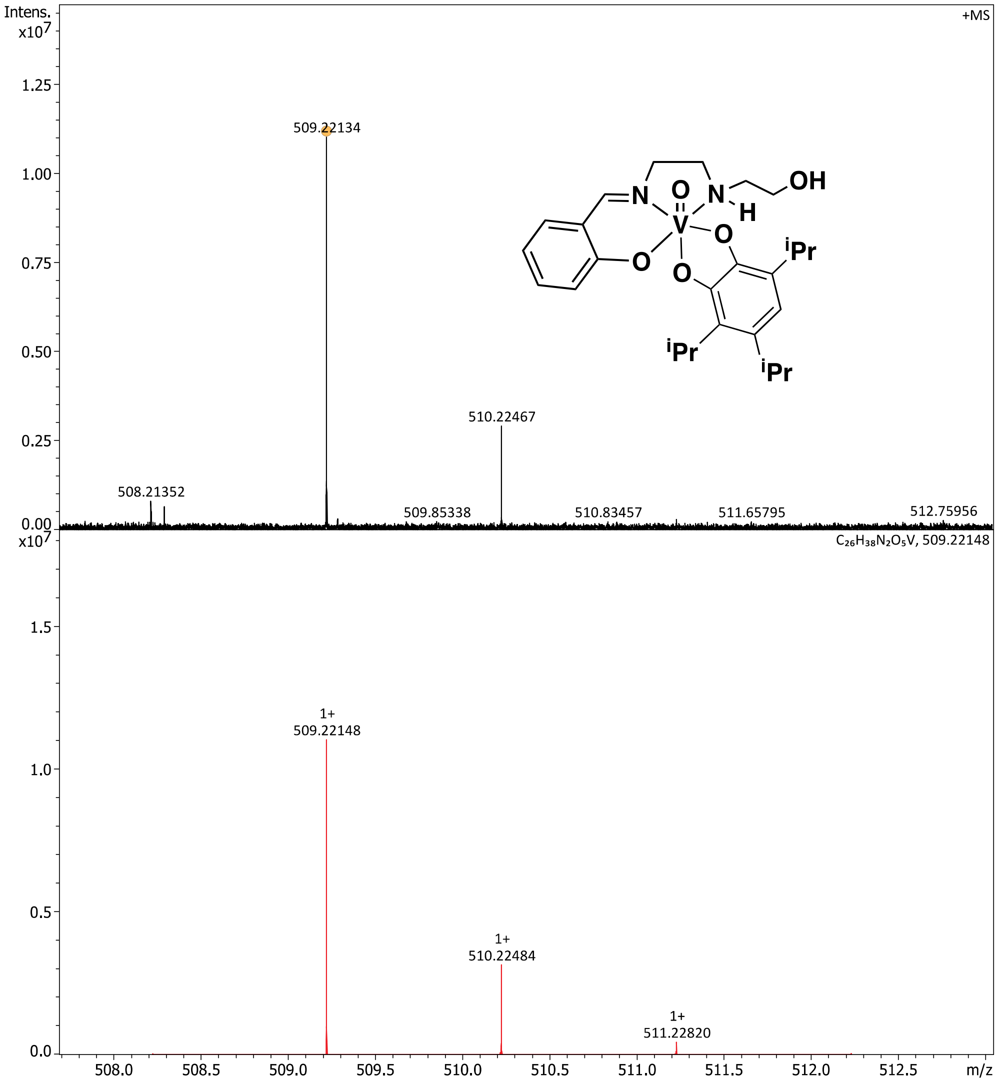

Bruker Compass DataAnalysis 6.0 printed: 23-Jul-24 5:37:32 PM by: demo Page 1 of 1

Figure S35 Experimental (top) and simulated (bottom) HRMS spectra of [VO(HSHED)(TIPCAT)]

VIII. Global Kinetic Analysis

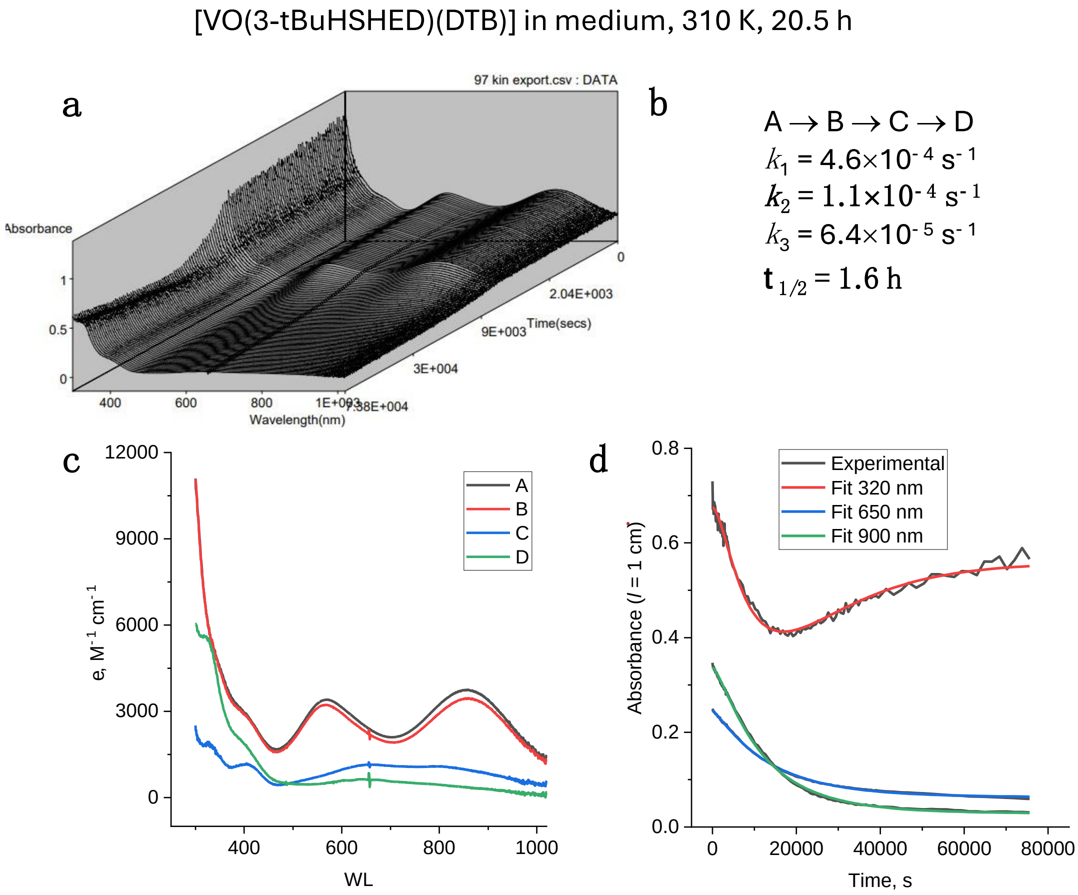

Figure S36. Global kinetic analysis of decomposition of [VO(3-tBuHSHED)(DTB)] (0.10 mM) in cell culture medium at 310 K: (**a**) three-dimensional plot of time-dependent spectra; (**b**) applied kinetic scheme and calculated parameters; (**c**) calculated spectra of initial, intermediate and final products; and (**d**) typical experimental and calculated kinetic curves. The first step (A → B) led to minor spectral changes and was likely due to the dissolution of the V(V) complex in the medium. The second step (B → C) led to disappearance of the characteristic peaks of [VO(3-tBuHSHED)(DTB)] at ~550 nm and ~850 nm and was regarded as the main decomposition step of the initial complex ($t_{1/2}$ = 1.6 h), likely leading to the release of $DTBH_2$ and the formation of $[V(O)_2(3\text{-tBuHSHED})]$ [4]. The third step (C → D) was due to secondary reactions: a decrease of absorbance at 300-400 nm was likely due to the dissociation of $[V(O)_2(3\text{-tBuHSHED})]$ and the release of V(V) species [4], and an increase of absorbance at 600-800 nm was likely due to the formation of $[V(DTB)_3]^-$ and $[VO(DTB)_2]^-$ [5].

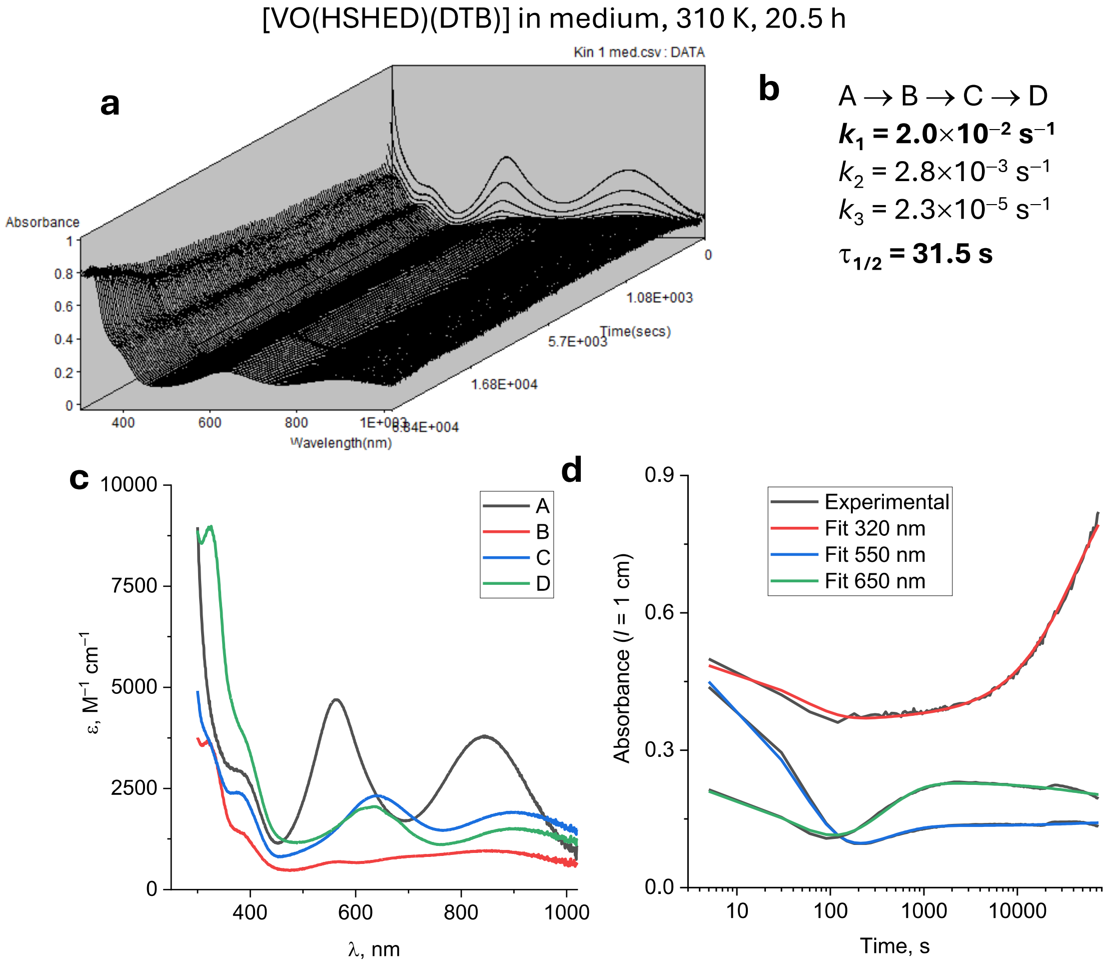

Figure S37. Global kinetic analysis of decomposition of [VO(HSHED)(DTB)] (0.10 mM) in cell culture medium at 310 K: (**a**) three-dimensional plot of time-dependent spectra; (**b**) applied kinetic scheme and calculated parameters; (**c**) calculated spectra of initial, intermediate and final products; and (**d**) typical experimental and calculated kinetic curves. The first step (A → B) led to disappearance of the characteristic peaks of [VO(HSHED)(DTB)] at ~550 nm and ~850 nm and was regarded as the main decomposition step of the initial complex ($t_{1/2}$ = 31.5 s), likely producing $[V(O)_2(HSHED)]$ and $DTBH_2$ [4]. The second step (B → C) led to an increase of absorbance at 600-800 nm due to the formation of $[V(DTB)_3]^-$ and $[VO(DTB)_2]^-$. The third step (C → D) led to partial decrease of absorbance at 600-800 nm and an increase of absorbance at 300-400 nm, likely due to catechol oxidation products (semiquinone and quinone) [4].

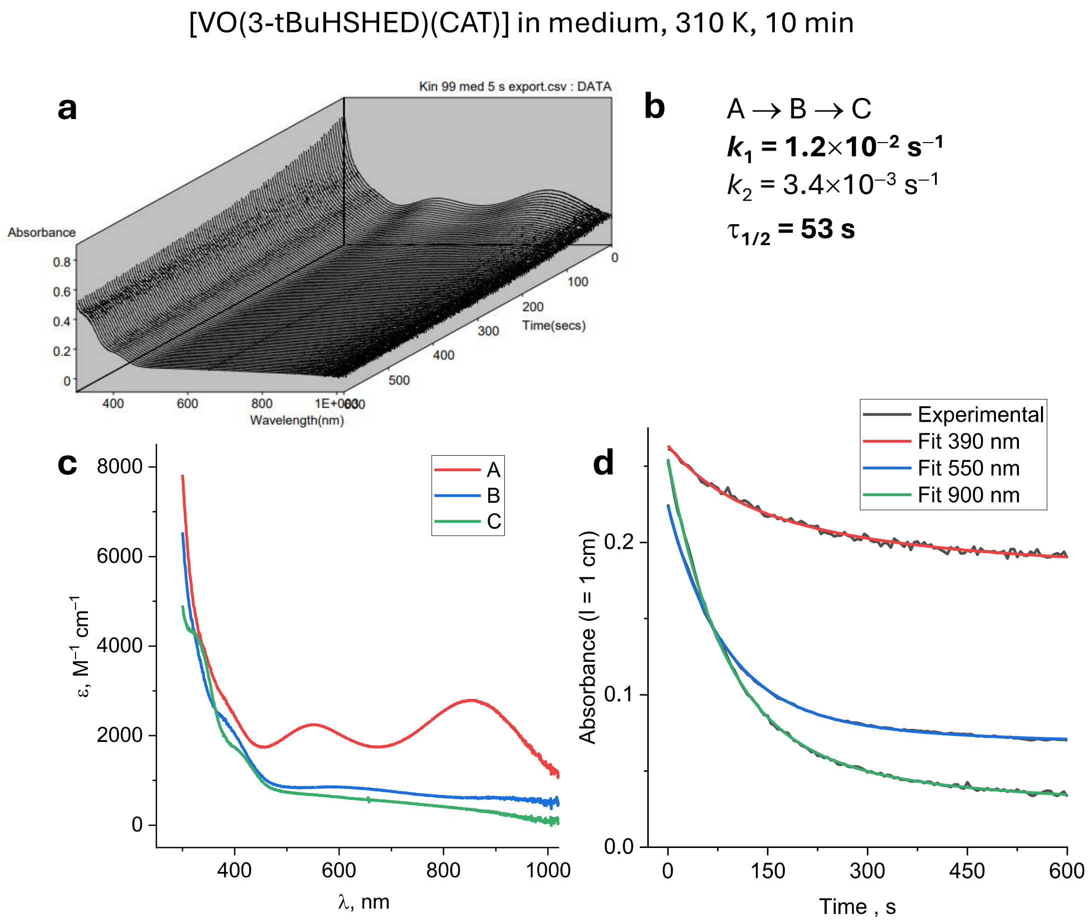

Figure S38. Global kinetic analysis of decomposition of [VO(3-tBuHSHED)(CAT)] (0.10 mM) in cell culture medium at 310 K: (**a**) three-dimensional plot of time-dependent spectra; (**b**) applied kinetic scheme and calculated parameters; (**c**) calculated spectra of initial, intermediate and final products; and (**d**) typical experimental and calculated kinetic curves. The first step (A → B) led to disappearance of the characteristic peaks of [VO(3-tBuHSHED)(CAT)] at ~550 nm and ~850 nm and was regarded as the main decomposition step of the initial complex ($t_{1/2}$ = 53 s), likely producing [V(O)$_2$(3-tBuHSHED)] and CATH$_2$ [4]. The second step (B → C) led to minor spectral changes due to secondary reactions of the decomposition products. Unlike for the complexes containing sterically hindered catechol ligands, no formation of V(V)-catecholato complexes was observed [4].

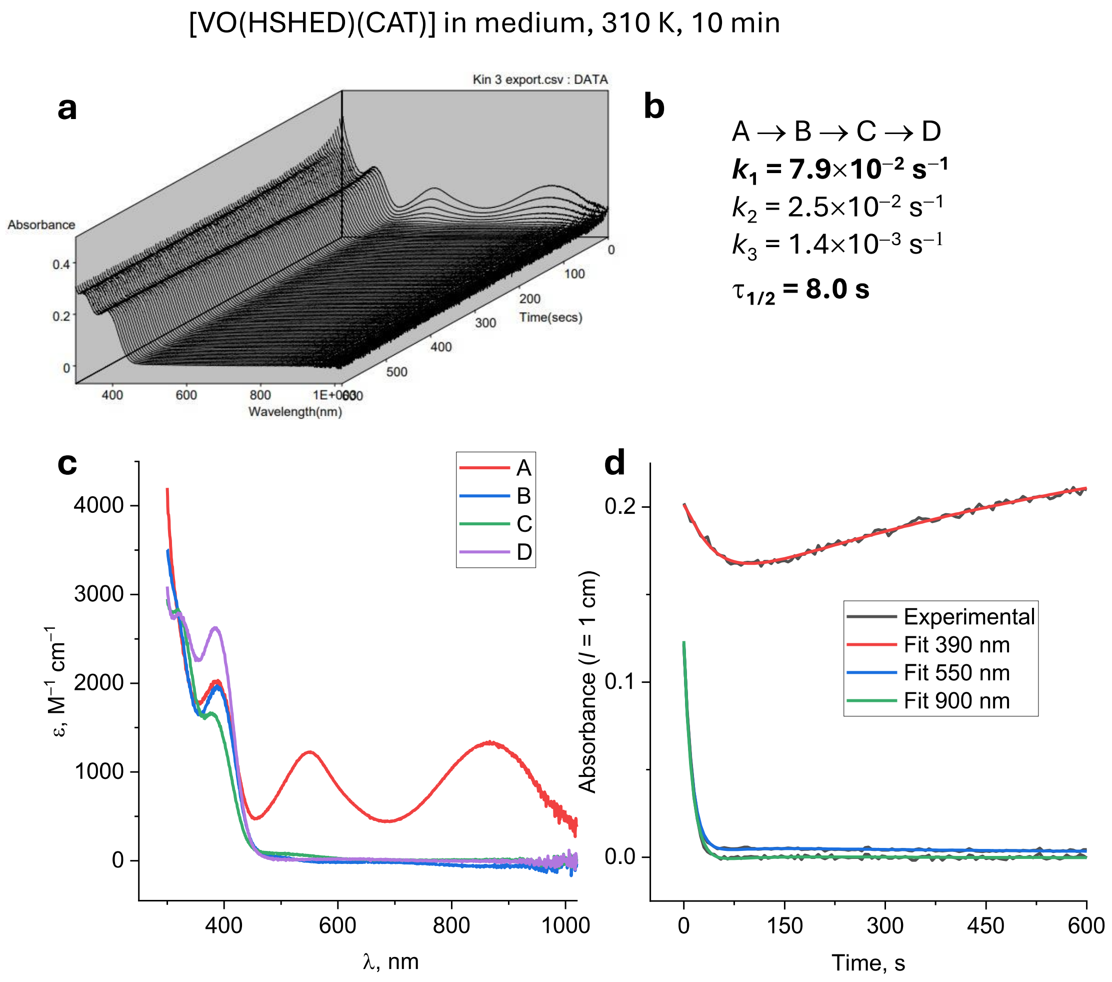

Figure S39. Global kinetic analysis of decomposition of [VO(HSHED)(CAT)] (0.10 mM) in cell culture medium at 310 K: (**a**) three-dimensional plot of time-dependent spectra; (**b**) applied kinetic scheme and calculated parameters; (**c**) calculated spectra of initial, intermediate and final products; and (**d**) typical experimental and calculated kinetic curves. The first step (A → B) led to disappearance of the characteristic peaks of [VO(HSHED)(CAT)] at ~550 nm and ~850 nm and was regarded as the main decomposition step of the initial complex ($t_{1/2}$ = 8.0 s), likely producing $[V(O)_2(HSHED)]$ and $CATH_2$ [4]. The second and third steps (B → C → D) were mostly due to spectral changes at ~400 nm, which were likely caused by the dissociation of $[V(O)_2(HSHED)]$ and the reactions of released V(V) species with free catechol, leading to colored oxidation products. Unlike for the complexes containing sterically hindered catechol ligands, no formation of V(V)-catecholato complexes was observed [4].

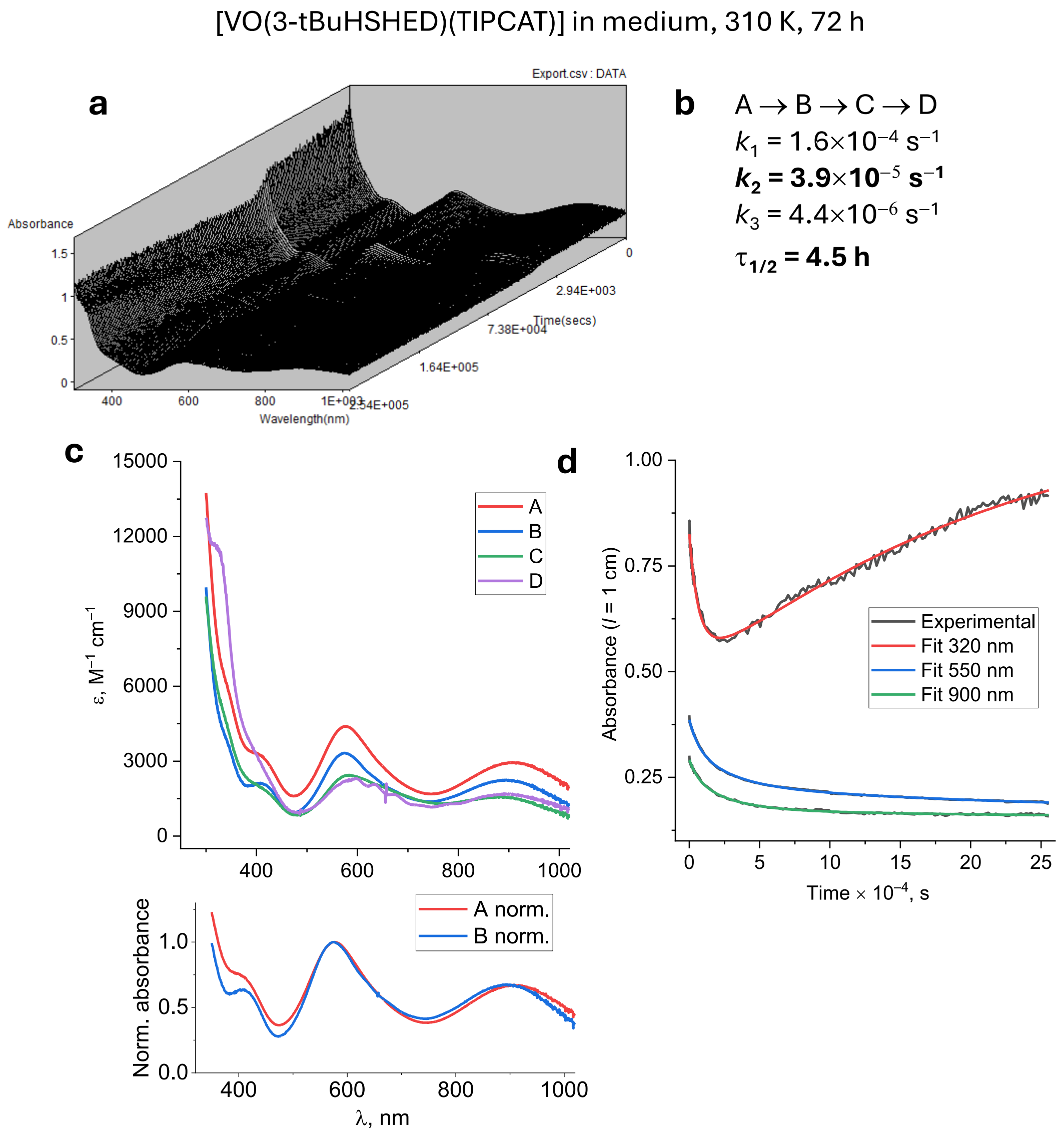

Figure S40. Global kinetic analysis of decomposition of [VO(3-tBuHSHED)(TIPCAT)] (0.10 mM) in cell culture medium at 310 K: (**a**) three-dimensional plot of time-dependent spectra; (**b**) applied kinetic scheme and calculated parameters; (**c**) calculated spectra of initial, intermediate and final products; and (**d**) typical experimental and calculated kinetic curves. The first step (A → B) led to a partial decrease in a characteristic absorbance of [VO(3-tBuHSHED)(TIPCAT)] at ~580 nm, but no shift in the absorbance maximum, as shown by comparison of normalized spectra in the lower part of (**c**). The second step (B → C) led to further decrease of absorbance in the visible range and a shift of absorbance maxima from ~580 nm to ~600 nm and from ~910 nm to ~880 nm, which was likely due to the formation of V(V)-TIPCAT complexes [4,5]. This step was regarded as the main decomposition step of the initial complex ($t_{1/2}$ = 4.5 h). However, this is likely an overestimation of the complex lifetime, as it partially decomposed in the first step. No significant absorbance changes in the visible range occurred after ~12 h of reaction, which is consistent with full decomposition of the initial complex within this time. The third step (C → D, 72 h timescale) led

to an absorbance increase in the 350-450 nm range, likely due to the formation of catechol oxidation products [4].

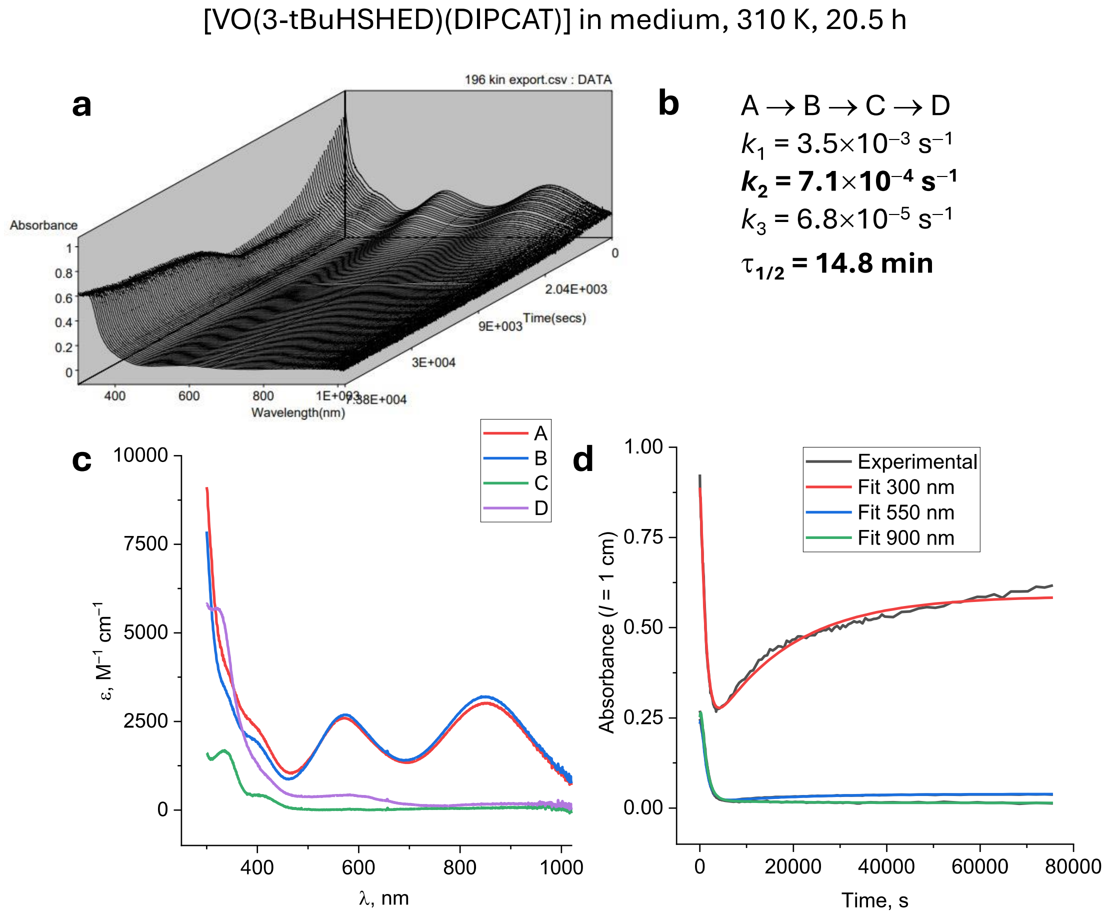

Figure S41. Global kinetic analysis of decomposition of [VO(3-tBuHSHED)(DIPCAT)] (0.10 mM) in cell culture medium at 310 K: (**a**) three-dimensional plot of time-dependent spectra; (**b**) applied kinetic scheme and calculated parameters; (**c**) calculated spectra of initial, intermediate and final products; and (**d**) typical experimental and calculated kinetic curves. The first step (A → B) caused minor spectral changes, likely due to dissolution of the complex in the medium. The second step (B → C) led to disappearance of characteristic absorbance maxima of [VO(3-tBuHSHED)(DIPCAT)] at ~550 nm and ~850 nm and was regarded as the main decomposition step of the initial complex ($t_{1/2}$ = 14.8 min), likely producing [V(O)$_2$(3-tBuHSHED)] and DIPCATH$_2$ [4]. The third step (C → D) led to an increase of absorbance at 300-400 nm, likely due to catechol oxidation products and the appearance of a shoulder at 450-650 nm, likely due to V(V)-DIPCAT complexes [5].

[VO(HSHED)(TIPCAT)] in medium, 310 K, 20.5 h

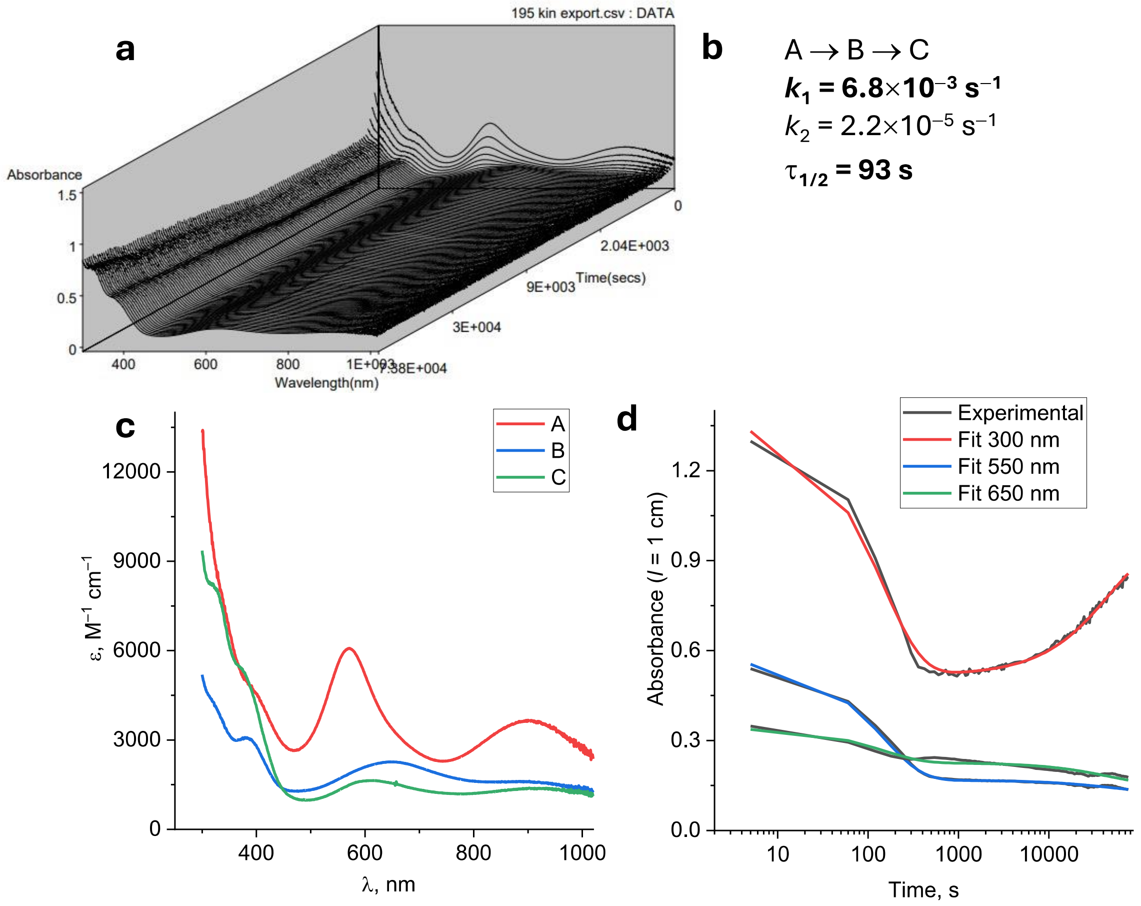

Figure S42. Global kinetic analysis of decomposition of [VO(HSHED)(TIPCAT)] (0.10 mM) in cell culture medium at 310 K: (**a**) three-dimensional plot of time-dependent spectra; (**b**) applied kinetic scheme and calculated parameters; (**c**) calculated spectra of initial, intermediate and final products; and (**d**) typical experimental and calculated kinetic curves. The first step (A → B) led to disappearance of characteristic absorbance maxima of [VO(3-tBuHSHED)(DIPCAT)] at ~550 nm and ~900 nm and was regarded as the main decomposition step of the initial complex ($t_{1/2}$ = 93 s), likely producing [V(O)$_2$(HSHED)] and TIPCATH$_2$ [4]. This step also led to absorbance maximum at ~630 nm, likely due to the parallel formation of [V^V(TIPCAT)$_3$]$^-$ complex [6]. Reactions of this complex in the second step (B → C) led new absorbance maxima at ~600 nm and ~900 nm (likely due to [V^{V}O(TIPCAT)$_2$]$^-$) and to shoulders at ~320 nm and ~380 nm (likely due to H$_2$SHED and catechol oxidation products) [4].

IX. Cell Viability

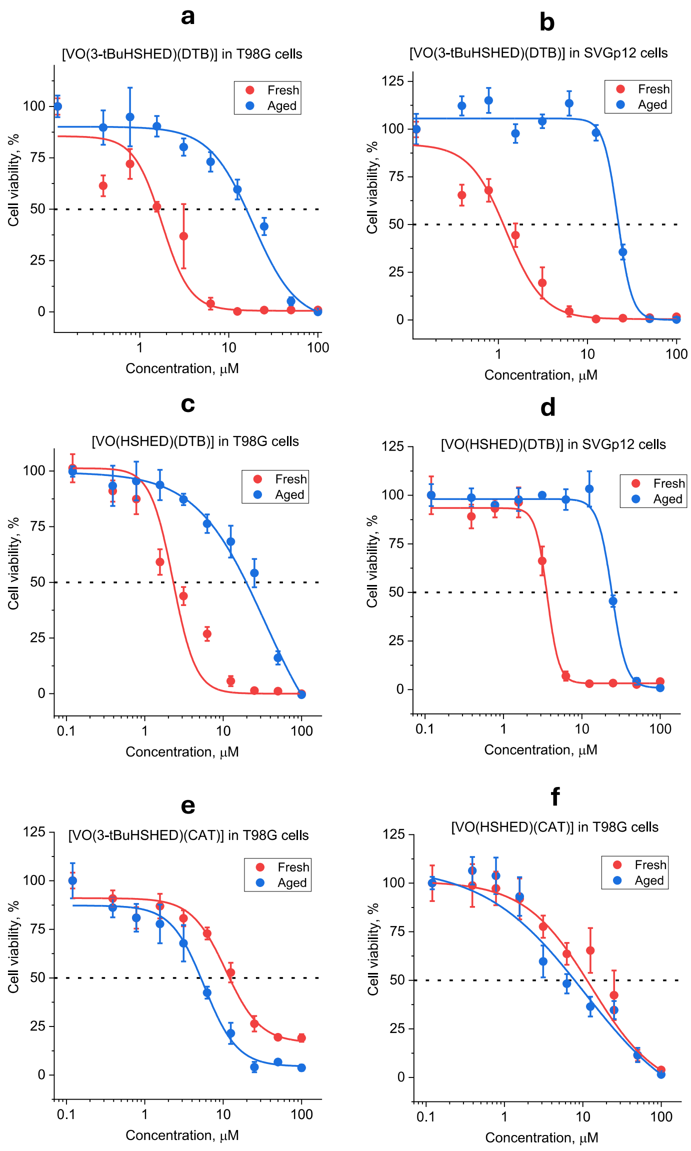

Figure S43. Typical concentration-viability plots for the anti-proliferative activities (72 h assays) of fresh (pre-incubated with the medium for < 1 min) and aged (pre-incubate with the medium for 24 h at 310 K) V(V) complexes: (**a**) [VO(3-tBuHSHED)(DTB)] in T98G cells; (**b**) [VO(3-tBuHSHED)(DTB)] in SVG p12 cells; (**c**) [VO(HSHED)(DTB)] in T98G cells; (**d**) [VO(HSHED)(DTB)] in SVG p12 cells; (**e**) [VO(3-tBuHSHED)(CAT)] in T98G cells; and (**f**) [VO(HSHED)(CAT)] in T98G cells. Dots and error bars represent the mean values and standard deviations of six replicate wells, and lines are the sigmoidal fits of experimental data, used for calculations of the IC_{50} values (Table 1 in the main text). Note that the complexes with CAT ligands

(**e** and **f**) were more active in the aged state, unlike for the complexes with DTB ligands (**a**-**d**), consistent with previous results [4].

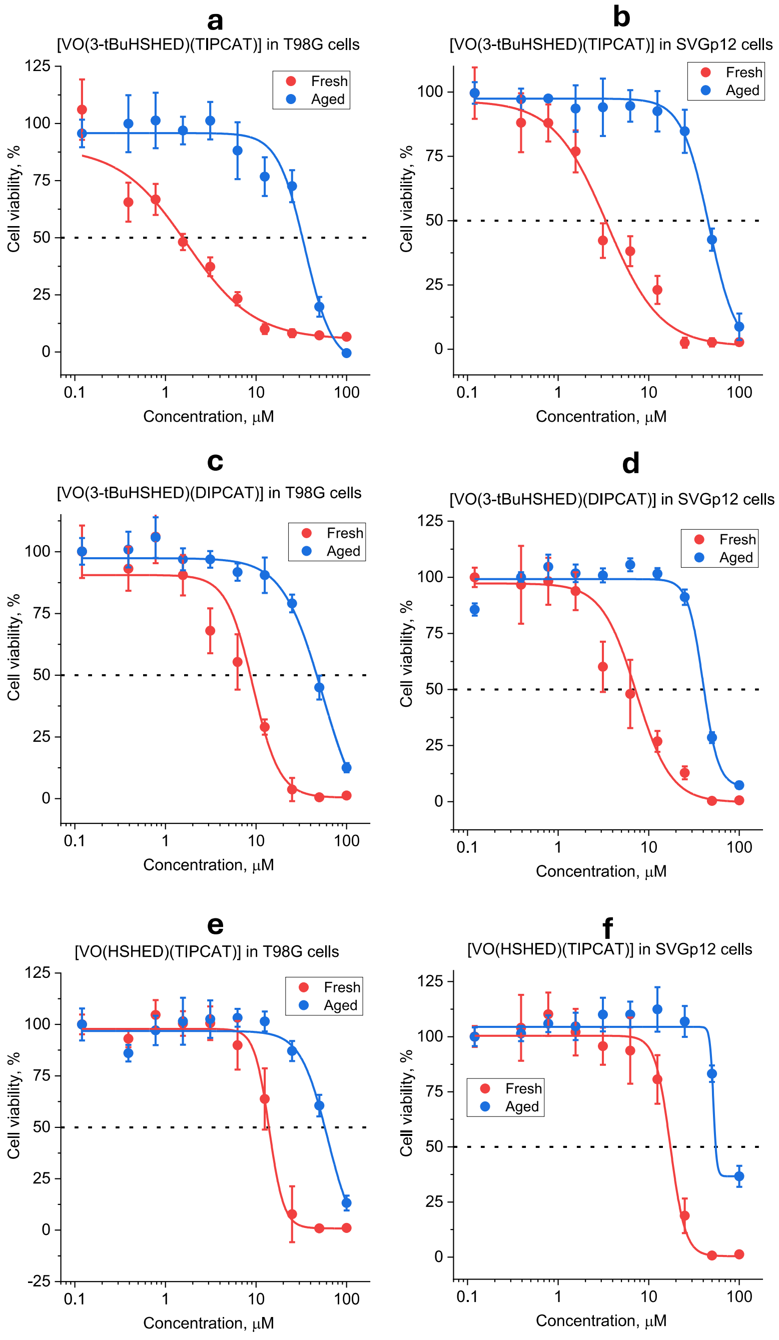

Figure S44. Typical concentration-viability plots for the anti-proliferative activities (72 h assays) of fresh (pre-incubated with the medium for < 1 min) and aged (pre-incubate with the medium for 24 h at 310 K) V(V) complexes: (**a**) [VO(3-tBuHSHED)(TIPCAT)] in T98G cells; (**b**) [VO(3-tBuHSHED)(TIPCAT)] in SVG p12 cells; (**c**) [VO(3-tBuHSHED)(DIPCAT)] in T98G cells; (**d**) [VO(3-tBuHSHED)(DIPCAT)] in SVG p12 cells; (**e**) [VO(HSHED)(TIPCAT)] in T98G cells; and (**f**)

[VO(HSHED)(TIPCAT)] in SVG p12 cells. Dots and error bars represent the mean values and standard deviations of six replicate wells, and lines are the sigmoidal fits of experimental data, used for calculations of the IC_{50} values (Table 1 in the main text).

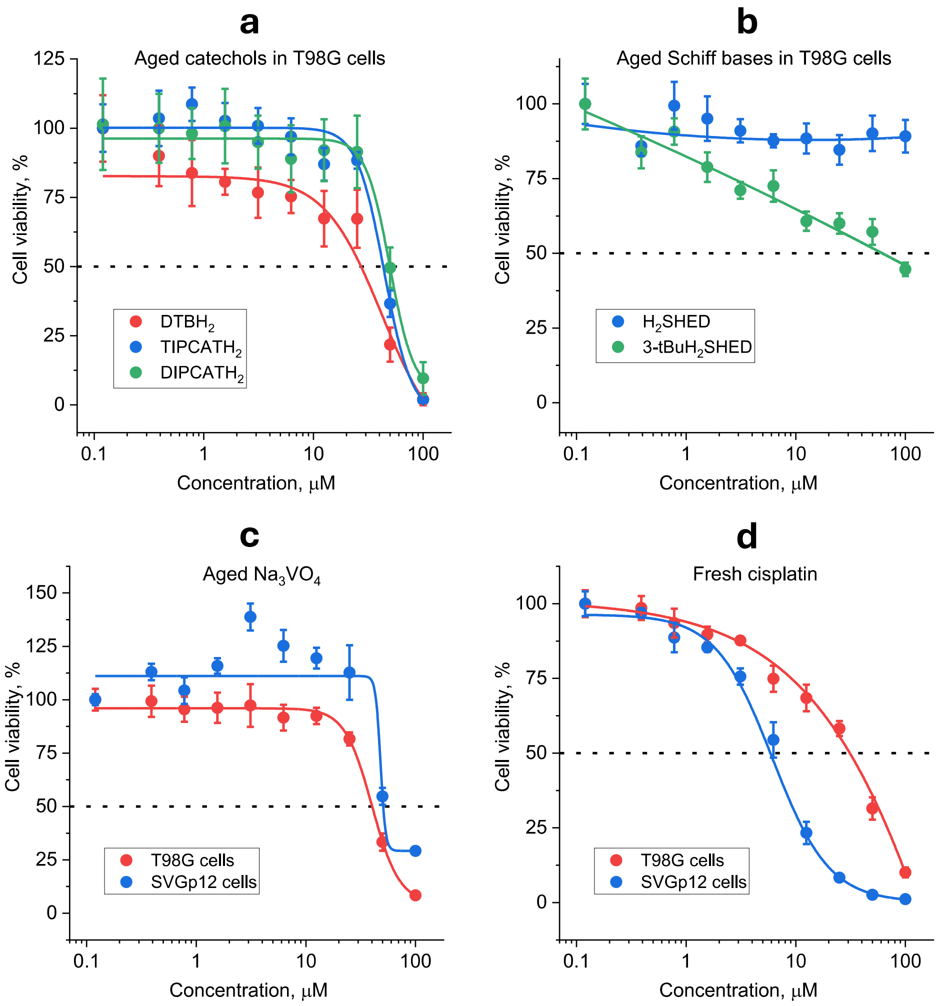

Figure S45. Typical concentration-viability plots for the anti-proliferative activities (72 h assays) of likely decomposition products (free ligands or vanadate aged in the medium for 24 h) [4] or a refence compound (cisplatin, freshly added to the medium) [6]: (**a**) catechol ligands in T98G cells; (**b**) Schiff base ligands in T98G cells; (**c**) Na_3VO_4 in T98G or SVG p12 cells; and (**d**) cisplatin in T98G or SVG p12 cells. Dots and error bars represent the mean values and standard deviations of six replicate wells, and lines are the sigmoidal or polynomial fits of experimental data, used for calculations of the IC_{50} values (Table 1 in the main text). In agreement with previous results [1,7], an increase in cell viability caused by subtoxic V(V) concentrations was observed in a non-cancer cell line, SVG p12, but not in a cancer cell line, T98G (**c**).

[1] H.A. Murakami, C. Uslan, A.A. Haase, J.T. Koehn, A.P. Vieira, D.J. Gaebler, J. Hagan, C.N. Beuning, N. Proschogo, A. Levina, P.A. Lay, D.C. Crans, Vanadium Chloro-Substituted Schiff Base Catecholate Complexes are Reducible, Lipophilic, Water Stable, and Have Anticancer Activities, Inorg Chem 61 (2022) 20757–20773. https://doi.org/10.1021/acs.inorgchem.2c02557.

[2] T.N. Kocherova, N.O. Druzhkov, A.S. Shavyrin, M. V. Arsenyev, E. V. Baranov, V.A. Kuropatov, V.K. Cherkasov, Isopropyl-substituted o-benzoquinones and oxanthrenequinones. Effect of steric shielding of alkyl substituents on reactivity, Russian Chemical Bulletin 70 (2021) 916–924. https://doi.org/10.1007/s11172-021-3167-6.

[3] Yu.A. Sayapin, N.B. Duong, E.A. Gusakov, I. V. Dorogan, V. V. Tkachev, V.S. Gorkovets, V.N. Komissarov, N.T. Duong, D.D. Nguyen, G. V. Shilov, S.M. Aldoshin, V.I. Minkin, Synthesis and structure of 5,7-diisopropyl-2-(quinolin-2-yl)-1,3-tropolone derivatives, Russian Chemical Bulletin 65 (2016) 2461–2468. https://doi.org/10.1007/s11172-016-1607-5.

[4] A. Levina, C. Uslan, H. Murakami, D.C. Crans, P.A. Lay, Substitution Kinetics, Albumin and Transferrin Affinities, and Hypoxia All Affect the Biological Activities of Anticancer Vanadium(V) Complexes, Inorg Chem 62 (2023) 17804–17817. https://doi.org/10.1021/acs.inorgchem.3c02561.

[5] E. Griffin, A. Levina, P.A. Lay, Vanadium(V) tris-3,5-di-tert-butylcatecholato complex: Links between speciation and anti-proliferative activity in human pancreatic cancer cells, J Inorg Biochem 201 (2019) 110815. https://doi.org/10.1016/j.jinorgbio.2019.110815.

[6] A. Levina, D. Crans, P. Lay, Advantageous Reactivity of Unstable Metal Complexes: Potential Applications of Metal-Based Anticancer Drugs for Intratumoral Injections, Pharmaceutics 14 (2022) 790. https://doi.org/10.3390/pharmaceutics14040790.

[7] K. Kostenkova, A. Levina, D.A. Walters, H.A. Murakami, P.A. Lay, D.C. Crans, Vanadium(V) Pyridine-Containing Schiff Base Catecholate Complexes are Lipophilic, Redox-Active and Selectively Cytotoxic in Glioblastoma (T98G) Cells, Chemistry – A European Journal 29 (2023). https://doi.org/10.1002/chem.202302271.
